# Intranasal delivery of the NMDA receptor antagonist MK-801 attenuates ultra-acute excitotoxic neurochemical responses after concussion in rats: comparative pharmacological evaluation against ketamine

**DOI:** 10.3389/fphar.2026.1764201

**Published:** 2026-03-16

**Authors:** Ian Masse, Luc Moquin, Caroline Bouchard, Laurianne Legroux, Alain Gratton, Louis De Beaumont

**Affiliations:** 1 Research Center, Hôpital du Sacré-Cœur de Montréal, Montreal, QC, Canada; 2 Research Center, Douglas Institute, Montreal, QC, Canada

**Keywords:** concussion, excitotoxicity, intranasal drug delivery, ketamine, microdialysis, MK-801, NMDA receptor antagonists

## Abstract

**Introduction:**

Concussion triggers rapid and transient surges in extracellular amino acids, largely driven by pathological activation of N-methyl-D-aspartate (NMDA) receptors. Targeting this ultra-acute excitotoxic window remains challenging, in part because many NMDA receptor antagonists exhibit slow delivery kinetics or limited brain penetrance when administered systemically. Intranasal drug delivery may help overcome these limitations by enabling faster central nervous system access. Here, we compared the capacity of intranasally administered MK-801, a high-affinity non-competitive NMDA receptor antagonist, and intranasal ketamine, a clinically used antagonist with distinct pharmacokinetic and receptor-binding properties, to modulate early neurochemical responses following experimental concussion.

**Materials and methods:**

Adult rats underwent a validated weight-drop concussion model followed by continuous hippocampal microdialysis. Extracellular glutamate, taurine, glycine, gamma-aminobutyric acid, glutamine, and serine were quantified every 10 min for 60 min before and after injury using high-performance liquid chromatography. Animals received intranasal MK-801 (10 mg/kg), ketamine (10 mg/kg), or vehicle immediately after concussion or sham procedures. Righting time was recorded as an early post-treatment behavioral outcome. MK-801 concentrations in plasma and brain were quantified by liquid chromatography-tandem mass spectrometry to assess central exposure. A composite excitotoxic index was calculated to summarize excitatory-inhibitory neurochemical imbalance.

**Results:**

Vehicle-treated concussed animals exhibited marked elevations in glutamate, taurine, and glycine within the first 10 min post-injury, accompanied by prolonged righting times. Intranasal MK-801 attenuated these ultra-acute neurochemical surges, reduced the composite excitotoxic index, and was associated with shorter righting times comparable to those observed in sham animals. In contrast, intranasal ketamine did not significantly alter amino acid dynamics or righting time under the conditions tested. MK-801 achieved measurable brain exposure following intranasal administration, with comparable brain-to-plasma ratios in sham and concussed animals.

**Discussion:**

Intranasal delivery of MK-801 modulates ultra-acute excitotoxic neurochemical disturbances following concussion in a rodent model. The lack of comparable effects with ketamine under the tested conditions highlights the importance of pharmacokinetic properties and receptor-binding kinetics when targeting the brief post-injury excitotoxic window. These findings provide proof-of-concept evidence supporting further investigation of rapid intranasal NMDA receptor antagonism as a strategy to influence early glutamatergic dysregulation after concussion.

## 1 Introduction

Concussions, or mild traumatic brain injuries, affect millions of individuals annually and remain a major public health challenge (Langlois et al., 2006). Although classified as “*mild*”, these injuries can produce immediate neurological impairment and are increasingly associated with persistent cognitive, affective, and functional symptoms, as well as elevated long-term risk of neurodegenerative conditions (McKee et al., 2013). Despite their prevalence and clinical burden, no pharmacological therapy has demonstrated consistent neuroprotective benefit in the acute phase of concussion (Maas et al., 2017), underscoring the need for mechanistically targeted interventions capable of acting within the earliest post-injury period (Giza and Hovda, 2014).

One of the earliest and most transient neurochemical disturbances following concussion is a rapid surge in extracellular glutamate, accompanied by alterations in taurine, glycine, gamma-aminobutyric acid (GABA), and other amino acids (Katayama et al., 1990). These changes reflect abrupt loss of ionic homeostasis and pathological activation of N-methyl-D-aspartate (NMDA) receptors (Giza and Hovda, 2014), leading to calcium, sodium, and potassium imbalances that initiate an excitotoxic cascade capable of contributing to acute neuronal dysfunction (Obrenovitch and Urenjak, 1997). Importantly, this glutamatergic dysregulation is extremely short-lived, with peak elevations occurring within minutes and typically normalizing within approximately 10 min in rodent concussion models (Nilsson et al., 1990). As a result, the feasibility of pharmacologically targeting this process depends not only on receptor antagonism but also on achieving sufficiently rapid central nervous system (CNS) exposure (Dhuria et al., 2010).

Several NMDA receptor antagonists have shown promise in preclinical models, including MK-801 (dizocilpine), a high-affinity, non-competitive channel blocker that stabilizes the receptor in a closed state and can additionally reduce presynaptic glutamate release (Wong et al., 1986). Despite this pharmacological potential, clinical translation has been limited, as systemically administered NMDA antagonists often fail to achieve effective brain concentrations within the narrow therapeutic window imposed by ultra-acute excitotoxicity (Ikonomidou and Turski, 2002). In previous work, prophylactic systemic administration of MK-801 effectively attenuated acute post-concussive amino acid disturbances, whereas therapeutic intraperitoneal administration after injury failed to produce comparable effects, underscoring the critical importance of delivery kinetics relative to the rapid onset of glutamatergic dysregulation (Masse et al., 2021). These findings suggested that the lack of efficacy observed with post-injury systemic dosing was not due to intrinsic pharmacological insufficiency, but rather to delayed CNS exposure. Building directly on this observation, the present study tested the hypothesis that intranasal (NAS) administration, by enabling more rapid brain access, could overcome this limitation and permit effective modulation of ultra-acute neurochemical responses when administered immediately after concussion.

NAS administration has emerged as a promising strategy for bypassing the blood-brain barrier and rapidly delivering drugs to central targets via olfactory and trigeminal pathways (Dhuria et al., 2010). In multiple seizure and neuroprotection models, NAS formulations achieve central penetration within 5–10 min, a timeframe that closely aligns with the transient excitotoxic peak observed after concussion (Chapman et al., 2013). These features make NAS delivery particularly well suited for mechanistic interventions aimed at the earliest post-injury phase (Hanson and Frey, 2008). Consistent with this rationale, recent work has highlighted growing interest in nose-to-brain delivery as a strategy to improve CNS exposure in acute neurological conditions, including traumatic brain injury (Qiu et al., 2025; Yoo et al., 2025).

Building on these observations, we developed and tested a novel NAS formulation of MK-801 optimized for rapid absorption and tolerability. Using a validated closed-skull weight-drop concussion model combined with continuous hippocampal microdialysis (MD), we quantified high-temporal-resolution changes in extracellular amino acids within the cornu ammonis 1 (CA1) region, a glutamate-rich structure with high NMDA receptor density (Masse et al., 2019). This approach enabled resolution of the immediate neurochemical trajectory following impact and allowed evaluation of the capacity of intranasally delivered MK-801 to modulate ultra-acute post-concussive neurochemical responses.

To determine whether modulation of ultra-acute post-concussive neurochemical responses was compound-specific or dependent on particular pharmacological properties, we conducted a parallel evaluation of intranasally administered ketamine at the same nominal dose (10 mg/kg). Although ketamine is also a non-competitive NMDA receptor antagonist, it differs markedly from MK-801 in channel affinity, dissociation kinetics, and breadth of pharmacological activity, including actions at opioid, cholinergic, hyperpolarization-activated cyclic nucleotide-gated (HCN), and monoaminergic systems (Zanos et al., 2018). Ketamine’s established clinical use and regulatory approval further make it a relevant comparator for examining how pharmacological profile and delivery kinetics interact during the ultra-acute post-injury window (Kohtala, 2021). Accordingly, this comparison was designed to contrast distinct NMDA antagonist properties under identical NAS delivery conditions, rather than to establish dose- or efficacy-equivalent effects, and to identify features required for rapid modulation of early post-concussive neurochemical disturbances.

This study incorporates three key advances: 1. use of a clinically relevant concussion model producing combined linear and rotational forces; 2. continuous, uninterrupted monitoring of amino acid dynamics during the first hour following impact; and 3. comparative evaluation of two intranasally delivered NMDA receptor antagonists with divergent pharmacological profiles. We hypothesized that NAS MK-801 would attenuate ultra-acute excitotoxic amino acid surges, normalize early post-injury physiological recovery, and reduce composite excitotoxic indices relative to vehicle-treated concussion animals. We further hypothesized that ketamine, although mechanistically related, would exhibit less robust modulation of these rapid neurochemical responses under the conditions tested, reflecting its lower NMDA receptor affinity and broader pharmacodynamic profile.

By integrating high-resolution neurochemical monitoring with rapid NAS drug delivery, this study aimed to determine whether NMDA receptor antagonism can meaningfully influence the earliest excitotoxic events following concussion and to identify the pharmacological and delivery features most relevant for future therapeutic development. To assess whether suppression of ultra-acute post-concussive excitotoxicity represents a generalizable property of NMDA receptor antagonism or depends on specific pharmacological characteristics, we performed a parallel evaluation of intranasally administered ketamine under identical dosing and temporal conditions as MK-801. Although both compounds are non-competitive NMDA receptor antagonists, they differ markedly in receptor affinity, inhibition constants, dissociation kinetics, and broader pharmacodynamic profiles. Accordingly, this comparison was explicitly designed to contrast distinct NMDA antagonist properties under rapid NAS delivery conditions, rather than to establish dose- or efficacy-equivalent effects. Within this framework, MK-801 served as a high-affinity pharmacological probe, while ketamine was included as a clinically relevant comparator to clarify how receptor-binding properties and delivery kinetics interact during the ultra-acute post-concussive window. These findings may help guide the rational design of rapid-onset, mechanism-based interventions for acute concussion management in both civilian and military contexts.

## 2 Materials and Methods

### 2.1 Animals, group allocation, and housing conditions

**FIGURE 1:**
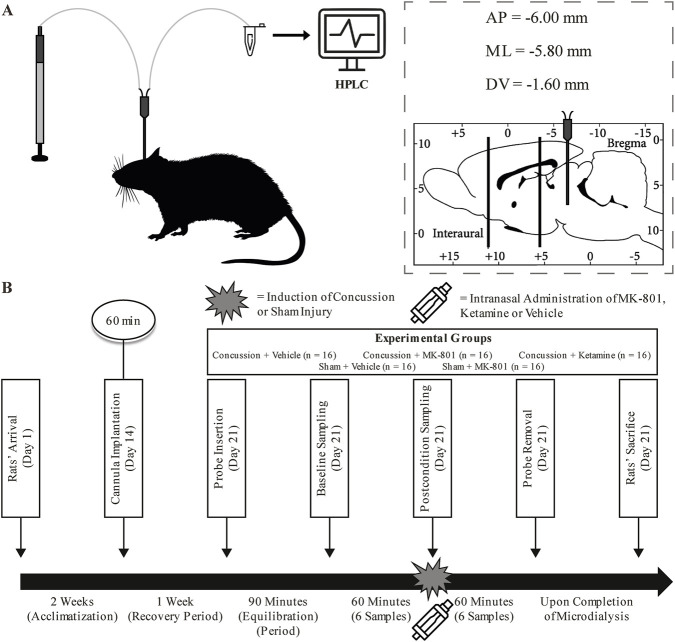
Experimental design. **(A)** Schematic representation of the *in vivo* cerebral microdialysis (MD) protocol performed on unanesthetized adult male Sprague Dawley rats, demonstrating the real-time monitoring of amino acid concentrations. **(B)** Schematic outline of the research protocol, illustrating the experimental groups, treatment administration, and injury induction procedures for the study.

All experimental procedures complied with the Canadian Council on Animal Care guidelines and were approved by the Hôpital du Sacré-Cœur de Montréal Animal Care Committee. Eighty adult male Sprague-Dawley rats (295–351 g; Charles River Laboratories) were housed under a 12-h light/dark cycle with *ad libitum* access to food and water and were acclimatized for 2 weeks prior to experimentation. Animals were randomly assigned to five experimental conditions (n = 16 per group): sham + vehicle, sham + MK-801, concussion + vehicle, concussion + MK-801, and concussion + ketamine. A sham + ketamine group was not included, as available pharmacokinetic (PK) data indicate that NAS ketamine achieves relatively modest brain concentrations at sub-anesthetic doses, raising concern about its capacity to meaningfully alter baseline amino-acid homeostasis or behavior in uninjured animals (Naidoo et al., 2019). Accordingly, experimental resources were focused on injured cohorts, which were central to the mechanistic aims of the study. The overall study design and experimental timeline are presented in Figure 1, and detailed group characteristics are provided in Supplementary Table S1.

### 2.2 Intranasal drug formulation and administration

MK-801 (10 mg/kg; Sigma-Aldrich) and ketamine (10 mg/kg; Bioniche Pharma) were dissolved in 40% polyethylene glycol 400 in acetate buffer (pH 4.5), and the same vehicle was administered to control animals. NAS administration was performed immediately after concussion or sham procedures, during the immediate post-impact period and prior to full resumption of spontaneous righting. Drugs were delivered using a custom NAS aerosolization device designed to generate fine droplets and promote nasal cavity deposition while minimizing oropharyngeal loss. The total aerosolized output delivered per animal ranged from 184 to 219 μL, administered as alternating aliquots of 92–110 µL per nostril over multiple brief pulses rather than as a single bolus. During dosing, animals were manually restrained in a supine position with the head slightly elevated to facilitate nasal delivery and limit runoff. Administration was completed within approximately 60 s of impact, after which animals were released and allowed to recover freely, aligning drug delivery with the rapid onset of the post-concussive glutamate surge.

### 2.3 Stereotaxic guide cannula implantation

Rats were anesthetized with 2.5% isoflurane and positioned in a stereotaxic frame. A 26-gauge guide cannula (P1 Technologies) was implanted targeting the right hippocampal CA1 region using standard Paxinos and Watson coordinates (AP: −6.0 mm; ML: −5.8 mm; DV: −1.6 mm) (Paxinos and Watson, 1998). The cannula assembly was secured with skull screws and dental cement, and patency was maintained using a stainless-steel obturator. Postoperative analgesia consisted of buprenorphine (0.05 mg/kg, subcutaneous, once daily for 2 days). Animals were allowed to recover for 1 week prior to MD procedures.

### 2.4 Microdialysis probe setup and *in vitro* recovery

Linear MD probes (13 kDa cutoff; 2.5 mm membrane; Spectrum Labs) were perfused with artificial cerebrospinal fluid (147 mM NaCl, 2.7 mM KCl, 1.2 mM CaCl_2_, 0.85 mM MgCl_2_; pH 7.4; 290–310 mOsm/kg) at a flow rate of 1 µL/min. All probes were tested *in vitro* prior to implantation to verify membrane integrity and relative recovery, which ranged from 14% to 19%. Probes were inserted into awake animals through the guide cannula, and a 90-min equilibration period preceded baseline sampling. Following completion of each experiment, probes were visually inspected upon explantation to confirm correct positioning and absence of gross damage. Datasets were screened for indicators of probe malfunction or sampling artifacts, including abrupt loss of signal across all analytes, persistent non-physiological baseline drift, or uniformly non-detectable concentrations suggestive of membrane failure or blockage. No experiments met these criteria, and no animals were excluded due to probe malfunction.

### 2.5 Baseline sampling, injury induction, and post-injury collection

Baseline dialysate samples were collected every 10 min for 60 min following probe equilibration. Concussion was induced using a closed-skull weight-drop model with unrestrained head and torso, producing combined linear and rotational forces, a protocol previously validated to produce a reproducible mild injury without skull fracture or hemorrhage (Masse et al., 2019). Sham animals underwent all procedures except the weight drop. Animals were fully awake and not anesthetized during sampling to preserve physiologically relevant neurochemical responses. Immediately after injury (or sham), animals received NAS MK-801, ketamine, or vehicle, and dialysate collection continued every 10 min for an additional 60 min. Each 10-µL dialysate sample was stabilized with 1 µL of 0.25 mol/L perchloric acid and stored at 4 °C until analysis. Righting time (loss of righting reflex duration) was recorded as an early post-treatment behavioral outcome reflecting the acute physiological state following concussion and NAS intervention. This measure was not used as an index of injury severity, neuroprotection, or long-term functional outcome. Because animals were briefly handled during NAS administration and because MK-801 and ketamine can acutely influence arousal and motor reflexes, righting time reflects a composite of injury-related, pharmacological, and handling-related effects and should be interpreted accordingly. Any bleeding, fractures, or mortality were documented. Although baseline sampling was conducted over 60 min to minimize early instability, gradual baseline variability and drift are inherent to *in vivo* MD in awake animals. Accordingly, baseline normalization was applied within animals to facilitate comparison of relative post-injury changes rather than assuming a static steady-state baseline.

### 2.6 Brain and plasma collection for MK-801 quantification

Following completion of MD, rats were anesthetized with intraperitoneal ketamine/xylazine (100/10 mg/kg) and perfused transcardially with 0.9% saline. Brains were rapidly harvested, flash-frozen, and stored at −80 °C until analysis. Approximately 2 mL of cardiac blood was collected into ethylenediaminetetraacetic acid-containing tubes, after which plasma was isolated by centrifugation and stored at −80 °C.

### 2.7 Liquid chromatography-tandem mass spectrometry quantification of MK-801

Plasma and brain samples were processed by protein precipitation using ice-cold acetonitrile. Plasma samples were mixed 1: 1 with acetonitrile, vortexed, incubated on ice for 10 min, and centrifuged at 14,000 rpm for 10 min at 4 °C. Brain tissue (0.5 g) was homogenized in 1 mL of acetonitrile and processed using the same procedure. Resulting supernatants were dried using a centrifugal evaporator and reconstituted in the mobile phase prior to analysis. MK-801 concentrations were quantified by liquid chromatography-tandem mass spectrometry using positive-ion electrospray ionization and multiple reaction monitoring. Calibration curves were generated using matrix-matched standards spiked with internal standards (clozapine and MK-801).

### 2.8 High-performance liquid chromatography quantification of amino acids

Dialysate samples were analyzed by high-performance liquid chromatography (HPLC) with precolumn derivatization using o-phthaldehyde and 3-mercaptopropionic acid, followed by fluorescence detection (excitation: 322 nm; emission: 455 nm) (Lupinsky et al., 2010). Samples were maintained at 4 °C following collection and derivatized immediately prior to HPLC injection. The analytical system consisted of a Dionex Ultimate 3,000 pump and autosampler coupled to a Waters Xterra MS C18 column (3.0 mm × 50 mm, 5 µm). The mobile phase comprised 3.5% acetonitrile, 20% methanol, and 100 mmol/L sodium phosphate dibasic (pH 6.7), delivered at a flow rate of 0.5 mL/min. Amino acids were identified based on retention time, with typical values of 1.8 min for glutamate, 1.6 min for glutamine, 2.2 min for glycine, 4.2 min for taurine, 5.8 min for serine, and 6.5 min for GABA. Quantification was performed using external calibration curves generated from amino acid standards prepared and derivatized under identical conditions as experimental samples, with standard curves spanning 7–500 ng/mL and demonstrating linearity (R^2^ = 0.9775–0.9974). No internal standard was used for amino acid quantification, consistent with established MD-HPLC protocols, and analyte concentrations were determined based on fluorescence peak area. Extracellular amino acid concentrations are reported as measured dialysate values and were not corrected for *in vitro* probe recovery, as analyses focused on relative within-animal and between-group changes over time rather than absolute extracellular concentrations. *In vitro* probe recovery was assessed prior to implantation to verify probe integrity and consistency across experiments but was not applied as a correction factor during quantification. Peaks were integrated and quantified using Chromeleon software.

### 2.9 Excitotoxic index

To capture the net excitatory-inhibitory neurochemical shift following concussion, a composite excitotoxic index was calculated as previously described (Globus et al., 1991):

$$\text{Excitotoxic Index}=\left(\text{Glutamate }x\text{ Glycine}\right)/\text{GABA}$$

This index integrates extracellular concentrations of glutamate, glycine, and GABA to provide a physiologically informed summary of ultra-acute neurochemical imbalance. Glutamate represents the primary excitatory ligand driving NMDA receptor activation, glycine functions as an obligatory NMDA receptor co-agonist that modulates receptor gating, and GABA provides a principal inhibitory counterbalance within local circuits. Accordingly, the index was designed to reflect the net balance of excitatory and inhibitory neurochemical perturbation during the ultra-acute post-injury period rather than to serve as a direct measure of excitotoxic injury or neuronal damage. Because the excitotoxic index is calculated as a ratio incorporating GABA concentrations, values approaching zero can increase numerical variability and inflate dispersion. The index was therefore used as a descriptive, heuristic measure of overall excitatory-inhibitory imbalance, with interpretation focused on group-level patterns rather than absolute values.

### 2.10 Statistical analysis

Statistical analyses were conducted using Jamovi (v2.3.28). Data distributions were assessed using the Shapiro-Wilk test, and homogeneity of variance was evaluated with Levene’s test. Depending on whether these assumptions were met, between-group comparisons were performed using independent-samples t-tests or Mann-Whitney U tests for two-group analyses, and one-way analysis of variance or Kruskal-Wallis tests for comparisons involving more than two groups. When nonparametric omnibus tests indicated significant effects, Dwass-Steel-Critchlow-Fligner (DSCF) *post hoc* tests were applied for multiple comparisons. Statistical significance was defined as p < 0.05 (two-tailed). The sample size (n = 16 per group) was selected based on prior studies using the same MD platform and concussion model, providing sufficient power to detect changes of approximately 20%–30% in extracellular amino acid concentrations. MD data consisted of repeated measurements collected from the same animals across time. Accordingly, statistical analyses were conducted with the primary aim of identifying ultra-acute group-level differences at discrete post-injury time points, rather than modeling longitudinal trajectories. Between-group comparisons at individual time points were performed using nonparametric tests to accommodate non-normal distributions, heteroscedasticity, and the presence of extreme values. No *a priori* or *post hoc* exclusion criteria were applied to extreme values, as large excursions in extracellular amino acid concentrations may reflect genuine biological variability following concussion rather than technical artifacts. Given the repeated-measures structure and multiple comparisons across time points, statistical findings should be interpreted cautiously, particularly at later intervals, with emphasis placed on early post-injury effects, consistency across analytes, and convergence with prior literature rather than reliance on isolated p-values.

## 3 Results

### 3.1 Histological examination and injury integrity

Macroscopic examination revealed no visible tissue damage attributable to either MD probe implantation or the concussion procedure. Hippocampal tissue integrity was preserved across all experimental groups, and no differences in gross pathology were observed between sham and concussed animals.

### 3.2 Righting time and early neurological recovery

**FIGURE 2:**
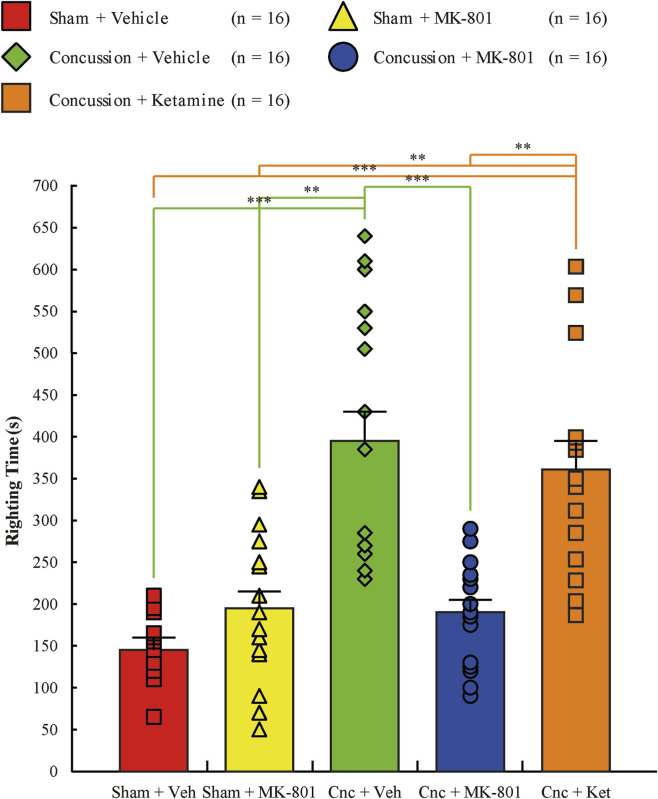
Histogram of righting time for the five treatment groups: sham injury with vehicle treatment (red squares, n = 16), sham injury with MK-801 treatment (yellow triangles, n = 16), concussion with vehicle treatment (green diamonds, n = 16), concussion with MK-801 treatment (blue circles, n = 16), and concussion with ketamine treatment (orange squares, n = 16). Error bars represent the standard error of the mean (SEM). *p < 0.05, **p < 0.01, ***p < 0.001.

Righting time, an early post-treatment behavioral measure reflecting acute physiological state, differed significantly among experimental groups (Kruskal-Wallis χ^2^ (4) = 41.60, p < 0.001; Figure 2; Supplementary Table S2). Values are reported as mean ± standard deviation. Vehicle- and ketamine-treated concussion animals exhibited the longest righting latencies (393 ± 156 s and 365 ± 145 s, respectively). In contrast, concussion animals receiving NAS MK-801 exhibited shorter righting times (190 ± 62 s), with values comparable to those observed in sham animals (147 ± 43 s for sham + vehicle; 196 ± 89 s for sham + MK-801). Post hoc DSCF analyses confirmed that both the concussion + vehicle and concussion + ketamine groups differed significantly from all other groups (all p < 0.01), whereas no significant differences were detected between the concussion + MK-801 group and either sham group. Righting time was analyzed solely as a post-treatment behavioral outcome and not as a measure of injury severity or neuroprotection. Accordingly, group differences reflect net acute physiological effects following concussion and NAS drug administration rather than injury magnitude alone. Full Kruskal-Wallis statistics and DSCF pairwise comparisons are provided in Table 1.

**TABLE 1:** Results of Kruskal-Wallis tests for between-group comparisons of righting times, extracellular concentrations of glutamate, GABA, taurine, glycine, glutamine, and serine, as well as excitotoxic indices. Significant pairwise differences identified by Dwass-Steel-Critchlow-Fligner (DSCF) *post hoc* comparisons are also reported.

Experimental groups	Time Point
Righting time	10
Kruskal-Wallis	<0.001***
Sham + Vehicle vs. Sham + MK-801	0.412
Sham + Vehicle vs. Concussion + Vehicle	<0.001***
Sham + Vehicle vs. Concussion + MK-801	0.248
Sham + Vehicle vs. Concussion + Ketamine	<0.001***
Sham + MK-801 vs. Concussion + Vehicle	0.005**
Sham + MK-801 vs. Concussion + MK-801	1.000
Sham + MK-801 vs. Concussion + Ketamine	0.007**
Concussion + Vehicle vs. Concussion + MK-801	<0.001***
Concussion + Vehicle vs. Concussion + Ketamine	0.985
Concussion + MK-801 vs. Concussion + Ketamine	0.003**

Experimental groups	Time points					
Glutamate	10	20	30	40	50	60
Kruskal-Wallis	<0.001***	0.021*	0.060	0.004**	0.033*	0.012*
Sham + Vehicle vs. Sham + MK-801	0.982	0.671	0.612	0.010*	0.095	0.118
Sham + Vehicle vs. Concussion + Vehicle	<0.001***	0.163	0.646	0.978	0.997	0.971
Sham + Vehicle vs. Concussion + MK-801	0.973	0.999	0.985	0.788	0.764	0.966
Sham + Vehicle vs. Concussion + Ketamine	0.002**	0.772	0.817	0.934	0.772	0.837
Sham + MK-801 vs. Concussion + Vehicle	<0.001***	0.026*	0.007**	<0.001***	0.005**	<0.001***
Sham + MK-801 vs. Concussion + MK-801	0.891	0.685	0.993	0.833	0.973	0.545
Sham + MK-801 vs. Concussion + Ketamine	<0.001***	0.136	1.000	0.067	0.565	0.406
Concussion + Vehicle vs. Concussion + MK-801	0.002**	0.536	0.737	0.713	0.782	0.934
Concussion + Vehicle vs. Concussion + Ketamine	0.145	0.933	0.216	0.880	0.607	0.200
Concussion + MK-801 vs. Concussion + Ketamine	0.032*	0.760	0.986	0.982	0.970	0.999

Experimental groups	Time points					
GABA	10	20	30	40	50	60
Kruskal-Wallis	0.053	0.380	0.011*	0.069	0.168	0.183
Sham + Vehicle vs. Sham + MK-801	1.000	1.000	0.994	0.899	0.434	0.954
Sham + Vehicle vs. Concussion + Vehicle	1.000	0.578	1.000	0.950	0.698	0.907
Sham + Vehicle vs. Concussion + MK-801	0.204	0.739	0.115	0.980	1.000	0.891
Sham + Vehicle vs. Concussion + Ketamine	0.992	0.405	0.995	0.560	0.684	0.448
Sham + MK-801 vs. Concussion + Vehicle	0.971	0.891	0.817	0.405	0.866	0.443
Sham + MK-801 vs. Concussion + MK-801	0.187	0.864	0.195	0.999	0.545	0.644
Sham + MK-801 vs. Concussion + Ketamine	0.998	0.744	0.950	0.045*	0.559	0.194
Concussion + Vehicle vs. Concussion + MK-801	0.072	0.944	0.012*	0.737	0.434	0.999
Concussion + Vehicle vs. Concussion + Ketamine	0.999	0.979	0.969	0.735	1.000	0.997
Concussion + MK-801 vs. Concussion + Ketamine	0.099	0.997	0.025*	0.242	0.564	0.791

Experimental groups	Time points					
Taurine	10	20	30	40	50	60
Kruskal-Wallis	<0.001***	<0.001***	0.008**	0.008**	0.017*	0.240
Sham + Vehicle vs. Sham + MK-801	0.989	0.516	0.685	0.995	0.739	0.980
Sham + Vehicle vs. Concussion + Vehicle	<0.001***	0.163	0.333	0.136	0.724	0.748
Sham + Vehicle vs. Concussion + MK-801	0.187	1.000	0.907	0.764	0.957	0.999
Sham + Vehicle vs. Concussion + Ketamine	0.002**	0.457	0.955	0.837	1.000	0.875
Sham + MK-801 vs. Concussion + Vehicle	<0.001***	0.002**	0.008**	0.032*	0.022*	0.240
Sham + MK-801 vs. Concussion + MK-801	0.371	0.098	0.131	0.188	0.205	0.738
Sham + MK-801 vs. Concussion + Ketamine	<0.001***	0.014*	0.102	0.205	0.319	0.484
Concussion + Vehicle vs. Concussion + MK-801	<0.001***	0.135	0.613	0.298	0.459	0.803
Concussion + Vehicle vs. Concussion + Ketamine	0.770	0.726	0.413	0.145	0.077	0.810
Concussion + MK-801 vs. Concussion + Ketamine	0.018*	0.510	1.000	1.000	0.976	0.997

Experimental groups	Time points					
Glycine	10	20	30	40	50	60
Kruskal-Wallis	<0.001***	0.013*	0.022*	0.010*	0.024*	0.044*
Sham + Vehicle vs. Sham + MK-801	0.347	0.034*	0.070	0.216	0.085	0.267
Sham + Vehicle vs. Concussion + Vehicle	<0.001***	0.968	0.533	0.953	0.922	0.052
Sham + Vehicle vs. Concussion + MK-801	0.997	0.865	0.999	0.791	0.999	0.909
Sham + Vehicle vs. Concussion + Ketamine	0.010**	0.985	0.994	0.944	0.830	0.391
Sham + MK-801 vs. Concussion + Vehicle	<0.001***	0.019*	0.326	0.034*	0.058	1.000
Sham + MK-801 vs. Concussion + MK-801	0.347	0.232	0.102	0.017*	0.102	0.484
Sham + MK-801 vs. Concussion + Ketamine	0.001**	0.038*	0.145	0.042*	0.200	0.909
Concussion + Vehicle vs. Concussion + MK-801	<0.001***	1.000	0.436	0.999	0.848	0.347
Concussion + Vehicle vs. Concussion + Ketamine	0.953	0.999	0.909	1.000	0.953	0.830
Concussion + MK-801 vs. Concussion + Ketamine	0.024*	0.998	0.909	1.000	0.880	0.810

Experimental groups	Time points					
Glutamine	10	20	30	40	50	60
Kruskal-Wallis	0.209	0.164	0.291	0.573	0.153	0.186
Sham + Vehicle vs. Sham + MK-801	0.997	0.991	0.991	0.999	1.000	0.922
Sham + Vehicle vs. Concussion + Vehicle	0.436	0.369	0.413	0.726	1.000	1.000
Sham + Vehicle vs. Concussion + MK-801	0.484	0.369	0.200	0.656	0.200	0.508
Sham + Vehicle vs. Concussion + Ketamine	0.112	0.880	0.865	0.988	0.968	0.999
Sham + MK-801 vs. Concussion + Vehicle	0.558	0.391	0.968	0.933	1.000	0.933
Sham + MK-801 vs. Concussion + MK-801	0.922	0.391	0.895	0.830	0.558	0.249
Sham + MK-801 vs. Concussion + Ketamine	0.968	0.865	0.996	1.000	0.999	0.656
Concussion + Vehicle vs. Concussion + MK-801	0.994	1.000	1.000	1.000	0.145	0.347
Concussion + Vehicle vs. Concussion + Ketamine	0.980	0.953	0.922	0.830	0.985	0.980
Concussion + MK-801 vs. Concussion + Ketamine	0.998	0.909	0.679	0.922	0.267	0.656

Experimental groups	Time points					
Serine	10	20	30	40	50	60
Kruskal-Wallis	0.072	0.746	0.559	0.123	0.329	0.644
Sham + Vehicle vs. Sham + MK-801	0.112	0.954	1.000	0.963	0.995	0.995
Sham + Vehicle vs. Concussion + Vehicle	0.988	0.996	0.922	0.988	0.726	0.988
Sham + Vehicle vs. Concussion + MK-801	0.631	0.997	0.991	0.770	0.996	0.922
Sham + Vehicle vs. Concussion + Ketamine	0.998	0.810	0.968	0.865	0.679	0.991
Sham + MK-801 vs. Concussion + Vehicle	0.240	0.997	0.944	0.986	1.000	1.000
Sham + MK-801 vs. Concussion + MK-801	0.638	0.954	0.999	0.638	0.997	0.782
Sham + MK-801 vs. Concussion + Ketamine	0.161	1.000	0.993	0.997	0.993	1.000
Concussion + Vehicle vs. Concussion + MK-801	0.865	0.999	0.558	0.038*	0.232	0.810
Concussion + Vehicle vs. Concussion + Ketamine	1.000	0.865	0.991	0.999	0.999	0.991
Concussion + MK-801 vs. Concussion + Ketamine	0.830	0.748	0.369	0.158	0.286	0.582

Experimental groups	Time points					
Excitotoxic index	10	20	30	40	50	60
Kruskal-Wallis	<0.001***	<0.001***	<0.001***	<0.001***	<0.001***	0.172
Sham + Vehicle vs. Sham + MK-801	0.823	0.989	0.950	0.970	0.989	0.995
Sham + Vehicle vs. Concussion + Vehicle	<0.001***	0.008**	0.003**	0.004**	<0.001***	0.432
Sham + Vehicle vs. Concussion + MK-801	0.966	0.995	0.997	0.891	1.000	0.998
Sham + Vehicle vs. Concussion + Ketamine	<0.001***	0.004**	0.010*	0.004**	<0.001***	0.322
Sham + MK-801 vs. Concussion + Vehicle	<0.001***	0.258	0.045*	0.237	0.325	0.946
Sham + MK-801 vs. Concussion + MK-801	0.998	1.000	1.000	0.992	1.000	1.000
Sham + MK-801 vs. Concussion + Ketamine	0.001***	0.031*	0.036*	0.106	0.168	0.621
Concussion + Vehicle vs. Concussion + MK-801	<0.001***	0.014*	0.076	0.410	<0.001***	0.434
Concussion + Vehicle vs. Concussion + Ketamine	0.995	0.200	0.985	0.735	0.809	0.999
Concussion + MK-801 vs. Concussion + Ketamine	<0.001***	0.006**	0.028*	0.137	<0.001***	0.311

*p < 0.05. **p < 0.01. ***p < 0.001.

### 3.3 Brain and plasma MK-801 exposure

**FIGURE 3:**
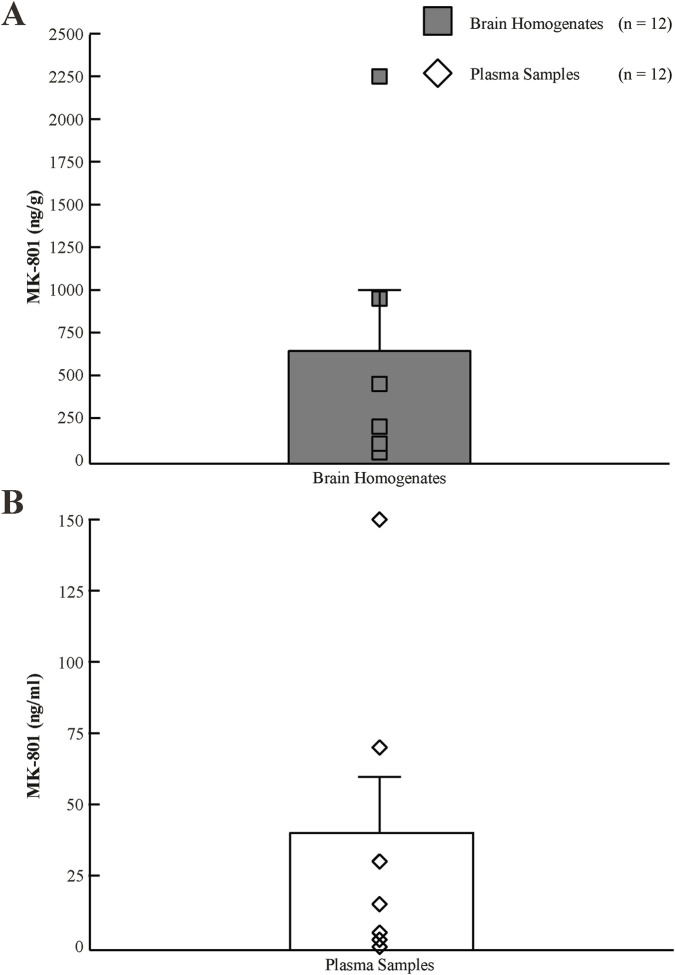
Calibration histograms for MK-801 concentrations in brain homogenates and plasma samples. **(A)** Calibration histogram depicting MK-801 concentrations in rat brain homogenates (gray squares, n = 12). **(B)** Calibration histogram showing MK-801 concentrations in plasma samples (white diamonds, n = 12).

**FIGURE 4:**
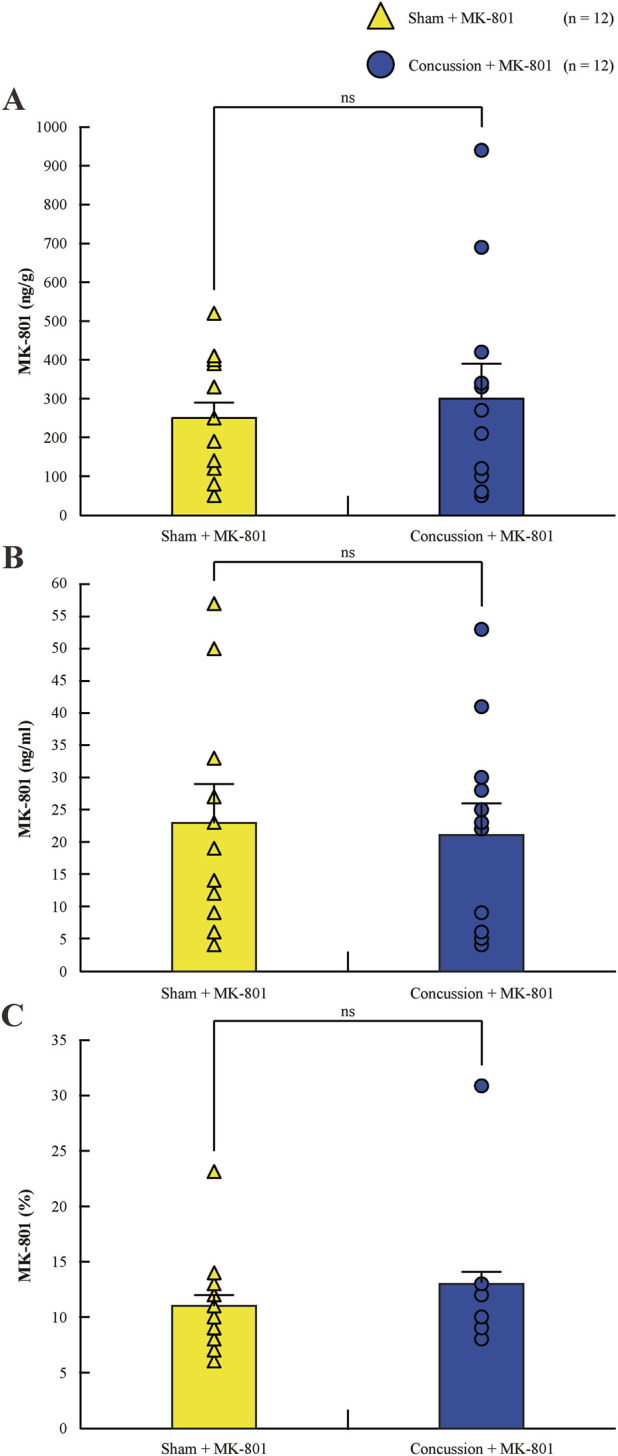
Histograms of MK-801 concentrations in brain homogenates and plasma samples for the sham injury with MK-801 treatment group (yellow triangles, n = 12) and the concussion with MK-801 treatment group (blue circles, n = 12). **(A)** Concentrations in rat brain homogenates. **(B)** Concentrations in rat plasma samples. **(C)** Brain-to-plasma ratio of MK-801 concentrations. Error bars represent the SEM. *p < 0.05, **p < 0.01, ***p < 0.001.

MK-801 was quantifiable in both plasma and brain homogenates following NAS administration (Figures 3, 4; Supplementary Tables S3, S4). Values are reported as mean ± standard deviation. Mean brain concentrations were 245.98 ± 159.09 ng/g in sham + MK-801 animals and 296.78 ± 276.25 ng/g in concussion + MK-801 animals, while corresponding plasma concentrations were 22.96 ± 16.71 ng/mL and 21.26 ± 15.40 ng/mL, respectively. No significant differences were observed between sham and concussion groups for brain concentrations (Mann-Whitney U = 71, p = 0.977), plasma concentrations (t (22) = 0.260, p = 0.797), or brain-to-plasma ratios (U = 61, p = 0.551). Together, these findings indicate that concussion status did not measurably alter MK-801 systemic exposure or central penetration following NAS administration.

### 3.4 Neurochemical dynamics in the acute post-concussion period

**FIGURE 5:**
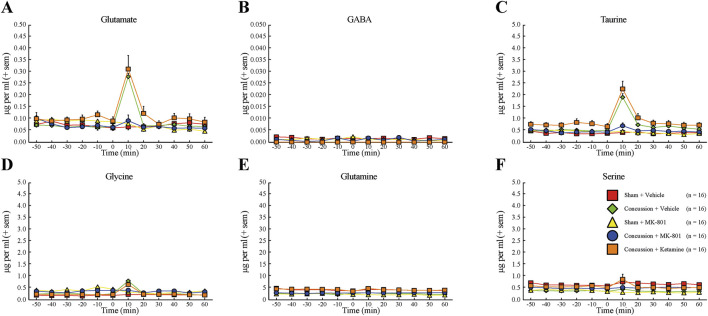
Mean levels (μg/mL) of hippocampal glutamate **(A)**, gamma-aminobutyric acid (GABA) **(B)**, taurine **(C)**, glycine **(D)**, glutamine **(E)**, and serine **(F)** measured by MD through baseline (60 min) and after sham injury with vehicle treatment (red squares, n = 16), sham injury with MK-801 treatment (yellow triangles, n = 16), concussion with vehicle treatment (green diamonds, n = 16), concussion with MK-801 treatment (blue circles, n = 16), and concussion with ketamine treatment (orange squares, n = 16) conditions (60 min). Error bars represent the SEM.

**FIGURE 6:**
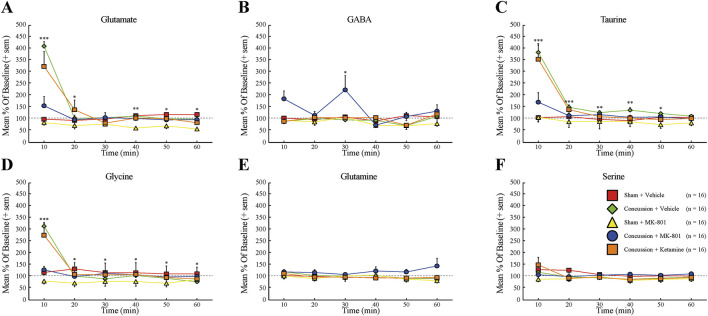
Mean percent change (+SEM) in contrast to baseline levels (60 min, normalized to 100%) of extracellular hippocampal glutamate **(A)**, GABA **(B)**, taurine **(C)**, glycine **(D)**, glutamine **(E)**, and serine **(F)** levels measured by MD after sham injury with vehicle treatment (red squares, n = 16), sham injury with MK-801 treatment (yellow triangles, n = 16), concussion with vehicle treatment (green diamonds, n = 16), concussion with MK-801 treatment (blue circles, n = 16), and concussion with ketamine treatment (orange squares, n = 16) conditions (60 min). Error bars represent the SEM. *p < 0.05, **p < 0.01, ***p < 0.001.

Continuous MD revealed marked elevations of glutamate, taurine, and glycine within the first 10 min following concussion in vehicle-treated animals, consistent with the canonical ultra-acute excitotoxic response. NAS MK-801 markedly attenuated these surges, whereas ketamine-treated rats displayed neurochemical profiles closely resembling those of vehicle-treated concussed animals. Absolute extracellular concentrations (µg/mL) are presented in Figure 5 and Supplementary Tables S5–S10, and percent-change values normalized to baseline are shown in Figure 6. Full Kruskal-Wallis and DSCF pairwise statistics for all amino acids and time points are provided in Table 1; asterisks in Figure 6 denote p-values from Kruskal-Wallis tests. Across glutamate, taurine, and glycine, concussion induced significant elevations relative to sham animals (all p < 0.05; Figures 6A,C,D). In each case, MK-801 prevented the injury-associated increases, yielding concentrations statistically indistinguishable from sham + vehicle controls (all p > 0.05). In contrast, ketamine did not reduce post-concussion surges under the tested dosing conditions, and values did not differ significantly from concussion + vehicle animals (all p > 0.05). Direct comparisons between MK-801 and ketamine demonstrated significant differences for glutamate, taurine, and glycine (all p < 0.05), indicating that MK-801, but not ketamine, modulated ultra-acute excitotoxic amino-acid dynamics at the tested dose. No significant between-group differences were observed for GABA, glutamine, or serine (Figures 6B,E,F), indicating that drug effects were selective for the glutamate-associated excitotoxic axis rather than reflecting global amino-acid disruption. Statistical comparisons at individual time points were performed to characterize ultra-acute group-level differences rather than to model longitudinal trajectories. Percent-change values were normalized to each animal’s pre-injury baseline to reduce inter-individual variability; however, baseline drift may contribute to dispersion in normalized values and should be considered when interpreting group differences.

### 3.5 Comparison to prophylactic MK-801 administration in previous work

**FIGURE 7:**
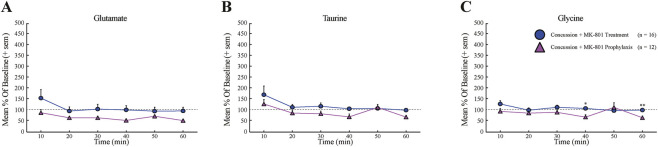
Mean percent change (+SEM) in contrast to baseline levels (60 min, normalized to 100%) of extracellular hippocampal glutamate **(A)**, taurine **(B)**, and glycine **(C)** levels measured by MD after concussion with MK-801 treatment (blue circles, n = 16), and concussion with MK-801 prophylaxis (purple triangles, n = 12) conditions (60 min). Error bars represent the SEM. *p < 0.05, **p < 0.01, ***p < 0.001.

To contextualize the magnitude of neurochemical modulation observed with post-injury NAS MK-801, extracellular amino acid levels were compared with data from a prior study in which MK-801 was administered systemically 60 min before injury (Masse et al., 2021). Post-injury glutamate, taurine, and glycine concentrations did not differ significantly between the prophylactic and post-injury MK-801 conditions (W = 73, p = 0.427; W = 64, p = 0.130; and W = 78, p = 0.268, respectively). As shown in Figure 7, these comparisons indicate that NAS administration immediately after injury achieved a degree of ultra-acute neurochemical modulation comparable to that observed with prophylactic systemic administration, despite the absence of pre-injury exposure.

### 3.6 Excitotoxic index

**FIGURE 8:**
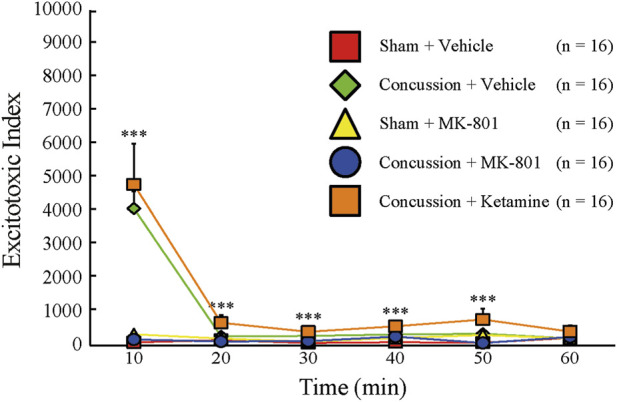
Change in the excitotoxic index over 60 min, calculated from the extracellular concentrations of glutamate, GABA, and glycine (µg/mL), as detailed in Supplementary Tables S5, S6, S8. The data represent conditions of sham injury with vehicle treatment (red squares, n = 16), sham injury with MK-801 treatment (yellow triangles, n = 16), concussion with vehicle treatment (green diamonds, n = 16), concussion with MK-801 treatment (blue circles, n = 16), and concussion with ketamine treatment (orange squares, n = 16). Error bars represent the SEM. *p < 0.05, **p < 0.01, ***p < 0.001.

The composite excitotoxic index demonstrated marked group differences at 10 min post-injury (Kruskal-Wallis χ^2^ (4) = 48.40, p < 0.001; Figure 8). Values are reported as mean ± standard deviation. Vehicle- and ketamine-treated concussed animals exhibited elevated excitotoxic index values (4092 ± 2037 and 4812 ± 4411, respectively), consistent with a pronounced shift toward excitatory neurochemical imbalance. In contrast, the concussion + MK-801 group displayed an index value (175 ± 290) that did not differ from sham + vehicle animals (90.4 ± 136; p = 0.966). These results indicate that NAS MK-801 markedly attenuated the early excitatory-inhibitory imbalance observed after concussion, as captured by this composite metric. The large variance observed in excitotoxic index values reflects, in part, low GABA concentrations in individual samples, which can amplify numerical dispersion inherent to ratio-based indices. Accordingly, group-level differences in the excitotoxic index were interpreted in conjunction with individual amino acid profiles rather than in isolation.

## 4 Discussion

In this study, we examined whether NAS administration of the high-affinity NMDA receptor antagonist MK-801 could modulate the ultra-acute excitotoxic neurochemical response that follows concussion in rats. Using continuous hippocampal MD, we show that NAS MK-801 rapidly attenuated post-injury elevations in extracellular glutamate, taurine, and glycine, reduced the composite excitotoxic index, and was associated with shorter righting times comparable to those observed in sham animals. In contrast, NAS ketamine, despite also functioning as a non-competitive NMDA receptor antagonist, did not significantly alter these neurochemical or behavioral measures under the conditions tested. Together, these findings indicate that both the pharmacological properties of the antagonist and the speed of CNS delivery are critical determinants for influencing the narrow ultra-acute excitotoxic window that follows concussive injury.

### 4.1 Early neurological recovery and functional relevance

Righting time, a commonly used early indicator of post-injury physiological recovery (Statler et al., 2000), was significantly prolonged in concussed animals treated with vehicle or ketamine, whereas animals receiving NAS MK-801 exhibited shorter righting times comparable to those observed in sham groups. Although shorter righting times occurred in parallel with attenuation of ultra-acute neurochemical disturbances, righting time remains a coarse, composite behavioral measure influenced by arousal state, sedation, dissociative effects, motor coordination, handling, and acute pharmacological action. Accordingly, this measure cannot dissociate injury-related recovery from drug-induced or motor effects and should not be interpreted as a functional correlate of neurochemical modulation or as evidence of neuroprotection. Importantly, no shortening of righting time was observed in sham animals treated with MK-801, suggesting that acute NAS dosing did not produce detectable behavioral impairment under these conditions. Nonetheless, righting time provides only a limited snapshot of early post-concussive physiology. In this context, prolonged righting times in vehicle-treated concussed animals likely reflect injury-induced suppression of arousal and motor responsiveness during the immediate post-impact period. Conversely, the shorter righting times observed following NAS MK-801 administration should not be construed as improved motor coordination or absence of drug effects, but rather as reflecting a net alteration of the acute post-injury physiological state. Given the coarse nature of this measure and the absence of dedicated motor or cognitive assessments, these findings do not establish long-term functional benefit, and broader behavioral implications remain to be determined.

### 4.2 Nasally administered MK-801 achieves consistent brain penetration

MK-801 concentrations in brain and plasma did not differ between sham and concussed animals, indicating that the concussive injury itself did not measurably alter systemic exposure or CNS entry following NAS administration. Comparable brain-to-plasma ratios further support the stability and reliability of the NAS route under both physiological and post-injury conditions (Illum, 2003). These PK findings underscore the practicality of NAS delivery for achieving rapid central exposure, which is particularly relevant in the context of concussion, where therapeutic delays of even minutes may limit the ability to influence ultra-acute pathophysiological processes (Ikonomidou and Turski, 2002; Dhuria et al., 2010; Maas et al., 2017). Future PK studies with higher temporal resolution will be required to more precisely define onset-of-action relative to the peak of post-concussive excitotoxicity.

### 4.3 MK-801 suppresses acute amino acid dysregulation after concussion

Consistent with established concussion literature (Katayama et al., 1990; Nilsson et al., 1990), vehicle-treated animals exhibited pronounced elevations of glutamate, taurine, and glycine within the first 10 min following impact. NAS MK-801 prevented these ultra-acute increases, yielding neurochemical profiles comparable to those observed in sham animals. The selectivity of these effects is notable, as each of the modulated metabolites plays a distinct role in excitatory signaling. Glutamate is the principal driver of NMDA receptor overactivation (Dingledine et al., 1999), glycine serves as an obligatory NMDA receptor co-agonist that amplifies receptor gating (Johnson and Ascher, 1987), and taurine is a neuromodulatory amino acid associated with osmotic stress responses, cell swelling, and oxidative processes that frequently accompany acute neural injury (Ramirez-Guerrero et al., 2022). In contrast, no significant changes were observed for GABA, glutamine, or serine, indicating that the early neurochemical signature in this model is dominated by glutamatergic dysregulation rather than broad perturbation across all amino acid systems. Consistent with these individual analyte findings, the composite excitotoxic index likewise shifted toward sham-like values following MK-801 administration, whereas vehicle- and ketamine-treated concussion groups exhibited marked elevations. Importantly, extracellular glutamate, glycine, and taurine arise from distinct cellular sources and subserve heterogeneous physiological roles, and their post-injury elevations should not be interpreted as uniformly or exclusively neurotoxic. While glutamate release after concussion is closely linked to neuronal depolarization and NMDA receptor overactivation, both glycine and taurine are substantially regulated by astrocytic mechanisms, including transporter-mediated release, osmotic regulation, and volume-sensitive efflux pathways activated during acute ionic and metabolic stress (Shibasaki et al., 2017; Rafiee et al., 2022). In this context, elevations in taurine may reflect an adaptive or compensatory response aimed at stabilizing cell volume and limiting excitability, rather than a direct contributor to excitotoxic injury. Accordingly, the observed amino acid dynamics are best interpreted as markers of an integrated, network-level neurochemical disturbance involving both neuronal and glial compartments, rather than as isolated indicators of irreversible excitotoxic damage. Neurochemical measurements in the present study were restricted to the hippocampal CA1 region, selected for its high NMDA receptor density, well-documented vulnerability to excitotoxic stress, and suitability for continuous high-temporal-resolution MD. While CA1 provides a sensitive readout of ultra-acute glutamatergic dysregulation after concussion, these findings should be interpreted as region-specific and not as direct evidence of global brain excitotoxicity. Other concussion-relevant regions, including neocortex, thalamus, and subcortical white matter, differ substantially in cellular composition, synaptic architecture, and receptor expression, and may therefore exhibit distinct temporal and quantitative neurochemical responses following injury. CA1 should thus be viewed as a sentinel region for interrogating early NMDA receptor-associated neurochemical disturbances, rather than as a surrogate for whole-brain excitotoxic processes.

### 4.4 Pharmacological distinctions between MK-801 and ketamine

Although MK-801 and ketamine are both non-competitive NMDA receptor antagonists, their pharmacodynamic and kinetic properties differ substantially, and these differences likely account for the divergent effects observed in this study. MK-801 is a high-affinity open-channel blocker that exhibits prolonged receptor occupancy and pronounced “*trapping*” behavior once bound within the NMDA receptor pore (Huettner and Bean, 1988). This results in sustained inhibition of NMDA-mediated calcium influx during periods of elevated glutamate release. In addition, blockade of presynaptic NMDA receptors by MK-801 can reduce regenerative glutamate release, thereby limiting injury-evoked glutamate efflux *in vivo* (Panter and Faden, 1992; Madara and Levine, 2008; Zhou et al., 2013). Together, these properties support MK-801’s capacity to maintain effective NMDA receptor blockade during the extremely brief excitotoxic window that follows concussion. Ketamine, by contrast, displays substantially lower NMDA receptor affinity, faster unbinding kinetics, and a shorter overall duration of action (Jones et al., 2001; Zorumski et al., 2016). Its pharmacological profile is also broader, encompassing interactions with opioid, monoaminergic, HCN, and cholinergic systems, which may reduce the specificity of its effects on NMDA-mediated excitatory signaling (Chen et al., 2009; Zorumski et al., 2016; Heifets et al., 2021; Subramanian et al., 2022). Under the single NAS dose used in this study (10 mg/kg), these properties likely limited ketamine’s ability to counter the rapid glutamate surge that characterizes the ultra-acute post-concussive phase. Given the absence of ketamine brain pharmacokinetic measurements and the use of a single intranasal dose, NMDA receptor target engagement by ketamine cannot be inferred under the present conditions. Accordingly, the lack of detectable ketamine effects should not be interpreted as evidence of pharmacological inefficacy, but rather as reflecting uncertain central exposure and potentially insufficient receptor engagement within the ultra-acute post-injury window examined. This interpretation is further constrained by the evaluation of only a single NAS dosing paradigm without inclusion of a sham-treated ketamine group (Yang et al., 2025). Insufficient central exposure, rapid receptor dissociation, or both may therefore have contributed to the observed lack of effect. Accordingly, the ketamine arm of this study should be viewed as a pharmacological comparator included to highlight the importance of receptor affinity, binding kinetics, and delivery speed in targeting ultra-acute excitotoxic mechanisms, rather than as a definitive assessment of ketamine’s therapeutic potential in concussion. Future studies incorporating dose-response analyses, repeated dosing strategies, or direct PK confirmation will be required to determine whether ketamine or related compounds can meaningfully modulate early excitotoxic dynamics when delivered intranasally. In contrast, MK-801 is best interpreted as a high-affinity pharmacological probe demonstrating proof-of-concept that sufficiently rapid and sustained NMDA receptor blockade can influence the earliest neurochemical events following concussive injury.

### 4.5 Comparisons to prophylactic MK-801 in previous work

Our prior study demonstrated that prophylactic systemic administration of MK-801 attenuated acute post-concussive amino acid disturbances (Masse et al., 2021). In the present study, NAS MK-801 administered immediately after injury produced a comparable magnitude of ultra-acute neurochemical modulation, despite the absence of pre-injury exposure. This finding underscores the mechanistic value of NAS delivery in overcoming the temporal limitations associated with systemic post-injury administration and highlights the importance of rapid CNS access for influencing the brief window of glutamatergic dysregulation that follows concussion.

### 4.6 Translational implications for intranasal neurotherapeutics

NAS administration represents a promising delivery platform for pharmacological interventions that require rapid CNS penetration. In contrast to systemic routes, which often produce delayed brain exposure or require higher doses to achieve central effects (Upadhyay, 2014; Achar et al., 2021; Shah et al., 2022), NAS delivery can achieve central penetration within minutes while potentially limiting systemic side effects (Djupesland et al., 2014; Crowe and Hsu, 2022; Qiu et al., 2025). Given the ultra-acute nature of post-concussive excitotoxicity, any intervention intended to influence early glutamatergic dysregulation must reach effective brain concentrations extremely rapidly. Within this context, intranasally delivered MK-801 demonstrated the capacity to modulate early neurochemical disturbances during the brief post-injury window examined. Importantly, while prophylactic administration of NMDA receptor antagonists can abolish glutamate surges, such preventive strategies are inherently impractical in clinical or athletic settings, particularly given the known cognitive and motor effects associated with potent NMDA antagonism (Lipton, 2004; Rowland et al., 2005). These considerations underscore the mechanistic relevance of a rapid post-injury NAS approach rather than pre-injury dosing. Although the present findings are limited to acute neurochemical and early behavioral outcomes, they provide proof-of-concept support for the development of fast-acting NAS formulations aimed at ultra-acute excitotoxic mechanisms (Yanamadala et al., 2024), with potential applicability to other conditions characterized by rapid glutamatergic dysregulation, such as stroke (Lai et al., 2014), neonatal hypoxia (Johnston et al., 2001), and epilepsy (Green et al., 2021). In this study, the composite excitotoxic index was used as a heuristic measure to summarize ultra-acute shifts in excitatory-inhibitory neurochemical balance rather than as a direct indicator of excitotoxic injury. While the amino acids incorporated into the index have distinct cellular sources and physiological roles, their integration provides a useful framework for comparing group-level differences in overall neurochemical disturbance during the immediate post-injury period.

### 4.7 Limitations and future directions

This study has several limitations that should be considered when interpreting the findings. First, all experiments were conducted in male rats, which limits generalizability given well-documented sex differences in concussion pathophysiology (Merritt et al., 2019; Mollayeva et al., 2021). Inclusion of female cohorts in future work will be necessary to determine whether the ultra-acute neurochemical and behavioral effects observed here are consistent across sexes. Second, the experimental design focused exclusively on the ultra-acute period, with outcomes assessed only during the first 60 min following injury. While this approach was well suited to capturing the transient excitotoxic surge that characterizes early concussion pathophysiology, it does not address longer-term behavioral, cognitive, inflammatory, or neuropathological consequences. Future studies incorporating extended timelines, repeated or chronic injury models, and comprehensive behavioral batteries will be required to determine whether early neurochemical modulation translates into sustained functional benefit. Third, behavioral assessment was limited to righting time, a coarse measure reflecting acute post-injury physiological state rather than neuroprotection, motor coordination, or cognitive function. Because righting time integrates injury-related suppression, arousal state, handling effects, and acute pharmacological influences, it cannot resolve drug-specific effects on sensorimotor performance or dissociative behavior. Dedicated motor and cognitive assessments will therefore be necessary to more fully characterize functional outcomes following NAS intervention. Fourth, interpretation of the ketamine findings is constrained by several factors. Only a single NAS ketamine dose was evaluated, brain exposure and NMDA receptor engagement were not directly quantified, and a sham-treated ketamine group was not included. Given the close correspondence between ketamine- and vehicle-treated neurochemical profiles, it is likely that the selected dose was insufficient to meaningfully engage NMDA receptors within the narrow ultra-acute window examined. Future studies incorporating dose-response analyses, repeated dosing paradigms, or PK confirmation will be required to more definitively assess ketamine’s capacity to modulate early excitotoxic dynamics. Additional methodological limitations relate to the MD approach itself. Neurochemical measurements were restricted to the hippocampal CA1 region, which provides a sensitive readout of NMDA receptor-associated excitotoxic processes but cannot be assumed to reflect uniform responses across other concussion-relevant brain regions. Moreover, baseline variability and gradual drift are inherent features of *in vivo* MD in awake animals. Although normalization to individual baselines reduces inter-animal variability, it does not fully eliminate baseline instability and should be interpreted alongside absolute concentration data. The repeated-measures structure of the MD data further increases susceptibility to Type I error when multiple comparisons are performed; accordingly, statistical findings should be interpreted in conjunction with effect directionality, temporal consistency, and biological plausibility rather than as isolated indicators of significance (Benjamini et al., 2001; Lazic, 2010). Interpretation of the composite excitotoxic index is also subject to limitations. As a ratio-based metric, the index is sensitive to low denominator values when GABA concentrations approach zero, which can inflate variance and numerical dispersion. The index was therefore used as a heuristic summary of excitatory-inhibitory neurochemical imbalance and should be interpreted alongside individual amino acid profiles rather than as a direct measure of excitotoxic injury. Finally, MK-801 was employed in the present study as a high-affinity NMDA receptor antagonist to interrogate the feasibility of rapidly modulating ultra-acute excitotoxic neurochemical responses following concussion. While potent NMDA receptor antagonism has historically been associated with psychotomimetic effects and safety concerns at certain doses and routes of administration, the present findings demonstrate that rapid intranasal delivery can achieve meaningful early neurochemical modulation within a narrowly defined post-injury window. Accordingly, these results should be viewed as proof-of-concept evidence underscoring the critical importance of delivery kinetics, formulation, and receptor engagement when targeting ultra-acute excitotoxic mechanisms. Future work will be required to optimize dosing, delivery parameters, and safety profiles, and to determine whether this intranasal formulation strategy can be further developed to support sustained therapeutic benefit.

## 5 Conclusion

This study demonstrates that NAS delivery of the high-affinity NMDA receptor antagonist MK-801 can modulate ultra-acute excitotoxic neurochemical responses that occur immediately after concussive injury in rats. NAS MK-801 rapidly attenuated post-injury elevations of glutamate, taurine, and glycine, reduced the composite excitotoxic index, and was associated with shorter righting times comparable to those observed in sham animals. Comparable brain-to-plasma ratios across injured and uninjured animals indicate that the NAS route provides reliable CNS exposure during the critical early period following concussion. Under the single-dose conditions examined here, NAS ketamine did not significantly alter amino acid dynamics or righting time, underscoring the importance of PK properties and receptor-binding kinetics when targeting the brief post-injury excitotoxic window. Alternative ketamine dosing strategies or formulations may yield different neurochemical profiles and warrant further investigation. Overall, these findings provide proof-of-concept evidence that sufficiently rapid intranasal delivery of a high-affinity NMDA receptor antagonist can modulate early glutamatergic dysregulation following concussion. Future studies will be required to determine whether such ultra-acute neurochemical modulation translates into longer-term functional benefit, to refine dosing and delivery parameters, and to further characterize safety and efficacy across extended time windows. More broadly, this work supports continued investigation of rapid-acting intranasal NMDA receptor modulation strategies for excitotoxic mechanisms in traumatic brain injury and related neurological conditions.

## Data Availability

The original contributions presented in the study are included in the article/Supplementary Material, further inquiries can be directed to the corresponding author.
