# Supplementary material for: Intranasal delivery of the NMDA receptor antagonist MK-801 attenuates ultra-acute excitotoxic neurochemical responses after concussion in rats: comparative pharmacological evaluation against ketamine
Source: Front Pharmacol. 2026 Mar 16;17:1764201. doi: 10.3389/fphar.2026.1764201 (PMC13033605; doi:10.3389/fphar.2026.1764201)
Supplement: Supplementary file 1 [file Table1.docx]

*SUPPLEMENTARY TABLE 1:* List of our cases analyzed by microdialysis (n = 80) and their general responsiveness.

| **Condition** | **Sham + Vehicle** | **Sham + MK-801** | **Concussion + Vehicle** | **Concussion + MK-801** | **Concussion + Ketamine** |
| --- | --- | --- | --- | --- | --- |
| Species | Rat | Rat | Rat | Rat | Rat |
| Strain | Sprague Dawley | Sprague Dawley | Sprague Dawley | Sprague Dawley | Sprague-Dawley |
| (n) | 16 | 16 | 16 | 16 | 16 |
| Mortality (%) | 0 | 0 | 0 | 0 | 0 |
| Skull fracture (%) | 0 | 0 | 0 | 0 | 0 |
| Bleed (%) | 0 | 0 | 0 | 0 | 0 |
| Neurological symptoms (%) | 0 | 0 | 0 | 0 | 0 |
| Mean righting time (s) | 146.56 | 195.94 | 392.81 | 189.69 | 364.69 |
