# Supplementary material for: Intranasal delivery of the NMDA receptor antagonist MK-801 attenuates ultra-acute excitotoxic neurochemical responses after concussion in rats: comparative pharmacological evaluation against ketamine
Source: Front Pharmacol. 2026 Mar 16;17:1764201. doi: 10.3389/fphar.2026.1764201 (PMC13033605; doi:10.3389/fphar.2026.1764201)
Supplement: Supplementary file 2 [file Table8.docx]

*SUPPLEMENTARY TABLE 8:* Extracellular concentrations of glycine (µg/ml).

| **Condition** | **Case** | **Time points** |  |  |  |  |
| --- | --- | --- | --- | --- | --- | --- |
|  |  | **-50** | **-40** | **-30** | **-20** | **-10** |
| Sham + | 01 | 0.173980000 | 0.144960000 | 0.095472000 | 0.104960000 | 0.121740000 |
| Vehicle | 02 | 0.251460000 | 0.249650000 | 0.228440000 | 0.201580000 | 0.187460000 |
|  | 03 | 0.057802000 | 0.036091000 | 0.046094000 | 0.021227000 | 0.023722000 |
|  | 04 | 0.159650000 | 0.066419000 | 0.088388000 | 0.081447000 | 0.064468000 |
|  | 05 | 0.337920000 | 0.352340000 | 0.422330000 | 0.278530000 | 0.331860000 |
|  | 06 | 0.173620000 | 0.161660000 | 0.163890000 | 0.177080000 | 0.670750000 |
|  | 07 | 0.260110000 | 0.217490000 | 0.259970000 | 0.238860000 | 0.246240000 |
|  | 08 | 0.101360000 | 0.107150000 | 0.105580000 | 0.124280000 | 0.083529000 |
|  | 09 | 0.221920000 | 0.206040000 | 0.123080000 | 0.080116000 | 0.147900000 |
|  | 10 | 0.180330000 | 0.178480000 | 0.186870000 | 0.175130000 | 0.217430000 |
|  | 11 | 0.067813000 | 0.027987000 | 0.023549000 | 0.024316000 | 0.021983000 |
|  | 12 | 0.241477330 | 0.237223120 | 0.278122232 | 0.251808588 | 0.224968721 |
|  | 13 | 0.241513153 | 0.251893141 | 0.270518660 | 0.269791313 | 0.275378968 |
|  | 14 | 0.200836109 | 0.256957379 | 0.271633472 | 0.194160832 | 0.253599347 |
|  | 15 | 0.253286656 | 0.217537897 | 0.221099857 | 0.221643668 | 0.220556046 |
|  | 16 | 0.294323975 | 0.177554211 | 0.251954320 | 0.195431990 | 0.211610360 |
|  | Mean ± SEM | 0.201087639 ± | 0.180589547 ± | 0.189811971 ± | 0.165022649 ± | 0.206449715 ± |
|  |  | 0.019550918 | 0.021985111 | 0.026302757 | 0.020750993 | 0.038416541 |
| Sham + | 01 | 0.085116049 | 0.088092285 | 0.094827572 | 0.090528848 | 0.068859553 |
| MK-801 | 02 | 0.132987243 | 0.147715603 | 0.090153146 | 0.148906312 | 0.143777175 |
|  | 03 | 0.131920000 | 0.115300000 | 0.133720000 | 0.126700000 | 0.132590000 |
|  | 04 | 0.308140000 | 0.200640000 | 0.212310000 | 0.150830000 | 0.140950000 |
|  | 05 | 0.125860000 | 0.133710000 | 0.118180000 | 0.167540000 | 0.147840000 |
|  | 06 | 0.436660000 | 0.452510000 | 0.422550000 | 0.380160000 | 0.401040000 |
|  | 07 | 0.195610000 | 0.259260000 | 0.164020000 | 0.103690000 | 0.102790000 |
|  | 08 | 0.410420000 | 0.377730000 | 0.424920000 | 0.436900000 | 0.350530000 |
|  | 09 | 0.383690000 | 0.576870000 | 0.558790000 | 0.627020000 | 0.826710000 |
|  | 10 | 0.101760000 | 0.296940000 | 0.139460000 | 0.156100000 | 0.119910000 |
|  | 11 | 0.194300000 | 0.298320000 | 0.215730000 | 0.207980000 | 0.119460000 |
|  | 12 | 0.763415460 | 0.305854040 | 0.595334972 | 1.568651750 | 1.671849792 |
|  | 13 | 0.316745810 | 0.712401219 | 1.252489457 | 0.310313973 | 1.360484071 |
|  | 14 | 0.940323895 | 0.404275488 | 1.434326163 | 0.218390092 | 1.047583189 |
|  | 15 | 0.471065594 | 0.736932563 | 0.674173429 | 0.782123746 | 1.849728906 |
|  | 16 | 1.741645700 | 0.724166342 | 0.569199830 | 0.455003721 | 0.495499486 |
|  | Mean ± SEM | 0.421228734 ± | 0.364419846 ± | 0.443761536 ± | 0.370677403 ± | 0.561225136 ± |
|  |  | 0.106643173 | 0.055256046 | 0.101374725 | 0.094188377 | 0.150858009 |
| Concussion + | 01 | 0.314546281 | 0.347202573 | 0.245646389 | 0.267212342 | 0.260172460 |
| Vehicle | 02 | 0.199676637 | 0.229132983 | 0.149361695 | 0.210354352 | 0.207819658 |
|  | 03 | 0.310916869 | 0.361804433 | 0.356019267 | 0.212737470 | 0.214606912 |
|  | 04 | 0.257654608 | 0.280188965 | 0.317476758 | 0.193158852 | 0.202068176 |
|  | 05 | 0.300174887 | 0.250845006 | 0.295315049 | 0.206198396 | 0.125086182 |
|  | 06 | 0.243689778 | 0.156490154 | 0.349841088 | 0.327702760 | 0.197193401 |
|  | 07 | 0.156776027 | 0.249499723 | 0.333193200 | 0.158314696 | 0.254973347 |
|  | 08 | 0.203911413 | 0.258294516 | 0.184589772 | 0.206501085 | 0.253174029 |
|  | 09 | 0.288906839 | 0.246179995 | 0.360982095 | 0.320897281 | 0.262671648 |
|  | 10 | 0.314300619 | 0.328074515 | 0.334385097 | 0.363910205 | 0.164066707 |
|  | 11 | 0.212977921 | 0.364128971 | 0.248830439 | 0.367317919 | 0.200870019 |
|  | 12 | 0.245860258 | 0.184588718 | 0.348840536 | 0.138782310 | 0.244059639 |
|  | 13 | 0.180457212 | 0.311964731 | 0.333914964 | 0.291481219 | 0.179234945 |
|  | 14 | 0.150465304 | 0.379644615 | 0.192199407 | 0.175905321 | 0.228302650 |
|  | 15 | 0.299767348 | 0.166464360 | 0.344890080 | 0.233756491 | 0.259567402 |
|  | 16 | 0.268443590 | 0.283675568 | 0.247943219 | 0.362726752 | 0.151055364 |
|  | Mean ± SEM | 0.246782850 ± | 0.274886239 ± | 0.290214316 ± | 0.252309840 ± | 0.212807659 ± |
|  |  | 0.014182019 | 0.017603730 | 0.017306808 | 0.019254161 | 0.010622590 |
| Concussion + | 01 | 0.358599718 | 0.481527549 | 0.246182851 | 0.600385960 | 0.783700707 |
| MK-801 | 02 | 0.086921000 | 0.097069000 | 0.095191000 | 0.105440000 | 0.084123000 |
|  | 03 | 0.727890000 | 0.662460000 | 0.678730000 | 0.633340000 | 0.722120000 |
|  | 04 | 0.386400000 | 0.345780000 | 0.511570000 | 0.394830000 | 0.424490000 |
|  | 05 | 0.127830000 | 0.111320000 | 0.088837000 | 0.144870000 | 0.134140000 |
|  | 06 | 0.139740000 | 0.113830000 | 0.088829000 | 0.108110000 | 0.083765000 |
|  | 07 | 0.220640000 | 0.144130000 | 0.249640000 | 0.117480000 | 0.077934000 |
|  | 08 | 0.075523000 | 0.055546000 | 0.082919000 | 0.063865000 | 0.082922000 |
|  | 09 | 0.081861000 | 0.095347000 | 0.088840000 | 0.096908000 | 0.069053000 |
|  | 10 | 0.358970000 | 0.187220000 | 0.080611000 | 0.226600000 | 0.215390000 |
|  | 11 | 0.213920000 | 0.321070000 | 0.238510000 | 0.185900000 | 0.261250000 |
|  | 12 | 0.291350000 | 0.300910000 | 0.229990000 | 0.165190000 | 0.193710000 |
|  | 13 | 1.421523008 | 0.687800059 | 0.298267967 | 1.085512224 | 1.256758254 |
|  | 14 | 0.162022675 | 0.628766896 | 1.317010119 | 1.332299284 | 0.579289460 |
|  | 15 | 1.014772140 | 0.352269370 | 0.196377133 | 0.524060123 | 1.360541768 |
|  | 16 | 0.277273063 | 0.719430903 | 0.334071940 | 0.326565847 | 0.164875545 |
|  | Mean ± SEM | 0.371577225 ± | 0.331529799 ± | 0.301598563 ± | 0.381959777 ± | 0.405878921 ± |
|  |  | 0.093902572 | 0.059097051 | 0.079568036 | 0.093587791 | 0.105579373 |
| Concussion + | 01 | 0.202640000 | 0.121670000 | 0.135630000 | 0.159010000 | 0.142450000 |
| Ketamine | 02 | 0.071440000 | 0.077754000 | 0.046228000 | 0.059286000 | 0.045793000 |
|  | 03 | 0.416080000 | 0.439440000 | 0.354580000 | 0.194370000 | 0.385420000 |
|  | 04 | 0.153042258 | 0.331011301 | 0.282606165 | 0.244665826 | 0.136964623 |
|  | 05 | 0.217699602 | 0.268530639 | 0.338196593 | 0.194179348 | 0.155024337 |
|  | 06 | 0.191415493 | 0.194490731 | 0.214425009 | 0.450849161 | 0.542565276 |
|  | 07 | 0.154080808 | 0.148302102 | 0.156730269 | 0.156016656 | 0.162163149 |
|  | 08 | 0.311749369 | 0.225206056 | 0.285130362 | 0.245777965 | 0.153725820 |
|  | 09 | 0.296047098 | 0.248528175 | 0.275845248 | 0.156585366 | 0.155079899 |
|  | 10 | 0.309495374 | 0.244028595 | 0.280597140 | 0.295887300 | 0.162573591 |
|  | 11 | 0.331063834 | 0.286304188 | 0.315363109 | 0.326812305 | 0.356700931 |
|  | 12 | 0.275264749 | 0.239346722 | 0.241146555 | 0.248940345 | 0.263319485 |
|  | 13 | 0.307819184 | 0.297963286 | 0.277796250 | 0.297107994 | 0.331247076 |
|  | 14 | 0.201174222 | 0.158538218 | 0.186275170 | 0.195104804 | 0.184068664 |
|  | 15 | 0.315445421 | 0.243154918 | 0.237436625 | 0.242539768 | 0.238456490 |
|  | 16 | 0.150533551 | 0.162793265 | 0.180382570 | 0.162581014 | 0.222362130 |
|  | Mean ± SEM | 0.244061935 ± | 0.230441387 ± | 0.238023066 ± | 0.226857116 ± | 0.227369654 ± |
|  |  | 0.022435849 | 0.022045008 | 0.020318189 | 0.022469912 | 0.030811837 |

| **Condition** | **Case** | **Time points** |  |  |  |  |
| --- | --- | --- | --- | --- | --- | --- |
|  |  | **0** | **10** | **20** | **30** | **40** |
| Sham + | 01 | 0.074420000 | 0.098954000 | 0.197710000 | 0.099880000 | 0.100450000 |
| Vehicle | 02 | 0.185470000 | 0.292950000 | 0.362690000 | 0.295210000 | 0.237910000 |
|  | 03 | 0.013837000 | 0.035704000 | 0.017558000 | 0.024765000 | 0.111690000 |
|  | 04 | 0.068661000 | 0.067415000 | 0.089239000 | 0.079358000 | 0.077372000 |
|  | 05 | 0.242470000 | 0.276080000 | 0.374280000 | 0.350890000 | 0.373570000 |
|  | 06 | 0.627690000 | 0.703290000 | 0.605430000 | 0.702930000 | 0.712880000 |
|  | 07 | 0.234980000 | 0.520170000 | 0.462450000 | 0.503080000 | 0.533540000 |
|  | 08 | 0.093793000 | 0.156680000 | 0.157670000 | 0.200060000 | 0.173390000 |
|  | 09 | 0.138650000 | 0.146230000 | 0.159630000 | 0.106040000 | 0.133310000 |
|  | 10 | 0.188080000 | 0.190350000 | 0.194960000 | 0.169720000 | 0.163920000 |
|  | 11 | 0.016958000 | 0.040096000 | 0.127630000 | 0.053866000 | 0.006752600 |
|  | 12 | 0.200128400 | 0.251859266 | 0.257069798 | 0.195824218 | 0.194140473 |
|  | 13 | 0.286758208 | 0.229522126 | 0.191604921 | 0.239956495 | 0.135082591 |
|  | 14 | 0.230929237 | 0.190095847 | 0.098382163 | 0.262572225 | 0.173217320 |
|  | 15 | 0.158119774 | 0.213975936 | 0.159044253 | 0.172462783 | 0.133682279 |
|  | 16 | 0.312888315 | 0.293304330 | 0.182033852 | 0.161321460 | 0.156576711 |
|  | Mean ± SEM | 0.192114558 ± | 0.231667282 ± | 0.227336374 ± | 0.226121011 ± | 0.213592748 ± |
|  |  | 0.036771296 | 0.043395709 | 0.038080786 | 0.043774126 | 0.045217084 |
| Sham + | 01 | 0.066403462 | 0.058181348 | 0.048859892 | 0.057528784 | 0.062712291 |
| MK-801 | 02 | 0.126660810 | 0.173111777 | 0.077746690 | 0.044144276 | 0.054357405 |
|  | 03 | 0.172760000 | 0.198320000 | 0.339130000 | 0.415410000 | 0.407490000 |
|  | 04 | 0.139970000 | 0.119340000 | 0.139770000 | 0.131960000 | 0.154240000 |
|  | 05 | 0.048630000 | 0.081958000 | 0.076014000 | 0.102390000 | 0.092641000 |
|  | 06 | 0.373270000 | 0.008253600 | 0.005669100 | 0.002709000 | 0.009898500 |
|  | 07 | 0.056501000 | 0.067642000 | 0.065203000 | 0.054386000 | 0.067740000 |
|  | 08 | 0.367910000 | 0.456990000 | 0.420980000 | 0.409030000 | 0.369160000 |
|  | 09 | 0.748680000 | 1.051800000 | 1.138500000 | 1.172800000 | 1.439500000 |
|  | 10 | 0.076183000 | 0.058999000 | 0.053090000 | 0.045337000 | 0.052325000 |
|  | 11 | 0.178370000 | 0.262150000 | 0.100300000 | 0.375623843 | 0.001235100 |
|  | 12 | 0.507818590 | 0.282684932 | 0.844251004 | 0.184214518 | 0.387147004 |
|  | 13 | 0.910317517 | 0.203506503 | 0.202656012 | 0.450698111 | 0.577899642 |
|  | 14 | 1.026436089 | 0.921311882 | 0.329671498 | 0.298132464 | 0.215183033 |
|  | 15 | 1.320068039 | 0.368864949 | 0.341542932 | 0.114949148 | 0.809472341 |
|  | 16 | 1.251098551 | 0.959858606 | 0.339044615 | 0.323266239 | 0.520181438 |
|  | Mean ± SEM | 0.460692316 ± | 0.329560787 ± | 0.282651796 ± | 0.261411211 ± | 0.326323922 ± |
|  |  | 0.111811492 | 0.086008213 | 0.077866502 | 0.072021066 | 0.095161384 |
| Concussion + | 01 | 0.250109472 | 0.727069860 | 0.312146322 | 0.246336904 | 0.344095257 |
| Vehicle | 02 | 0.349417273 | 0.888852398 | 0.248509499 | 0.295860280 | 0.249949475 |
|  | 03 | 0.193175694 | 0.777241646 | 0.293342428 | 0.313527351 | 0.250690515 |
|  | 04 | 0.203634465 | 0.710489086 | 0.262597683 | 0.319043048 | 0.305426435 |
|  | 05 | 0.297988801 | 0.712588494 | 0.252350043 | 0.151075386 | 0.190223149 |
|  | 06 | 0.319185431 | 1.145164545 | 0.224914659 | 0.318033531 | 0.223224646 |
|  | 07 | 0.259471640 | 0.482637429 | 0.155447559 | 0.144920712 | 0.247876974 |
|  | 08 | 0.351623590 | 0.994105975 | 0.185254006 | 0.183967579 | 0.258277700 |
|  | 09 | 0.348007539 | 1.111141963 | 0.299188880 | 0.203335353 | 0.251607095 |
|  | 10 | 0.264404954 | 0.836858845 | 0.219768107 | 0.136586228 | 0.265843767 |
|  | 11 | 0.219027666 | 1.066841680 | 0.231421648 | 0.166439486 | 0.334612278 |
|  | 12 | 0.306416599 | 0.785255116 | 0.220113086 | 0.220272954 | 0.180709814 |
|  | 13 | 0.206773889 | 0.727577719 | 0.339646976 | 0.319559647 | 0.358587902 |
|  | 14 | 0.212168723 | 0.620793715 | 0.353993863 | 0.188212287 | 0.328781442 |
|  | 15 | 0.255622429 | 0.753658372 | 0.315951851 | 0.308559242 | 0.338610156 |
|  | 16 | 0.188667476 | 0.615719199 | 0.269176951 | 0.158102367 | 0.147515004 |
|  | Mean ± SEM | 0.264105977 ± | 0.809749753 ± | 0.261488972 ± | 0.229614522 ± | 0.267251975 ± |
|  |  | 0.014524924 | 0.046924357 | 0.013798750 | 0.017972630 | 0.015823818 |
| Concussion + | 01 | 0.600365511 | 0.547517586 | 0.286972478 | 0.507859489 | 0.458283460 |
| MK-801 | 02 | 0.134070000 | 0.097357000 | 0.106560000 | 0.122310000 | 0.200630000 |
|  | 03 | 0.503160000 | 1.065400000 | 0.999550000 | 0.876670000 | 0.964770000 |
|  | 04 | 0.504790000 | 0.529970000 | 0.465660000 | 0.497890000 | 0.410350000 |
|  | 05 | 0.108070000 | 0.281920000 | 0.174320000 | 0.191790000 | 0.150810000 |
|  | 06 | 0.091982000 | 0.113700000 | 0.116430000 | 0.112490000 | 0.112750000 |
|  | 07 | 0.069498000 | 0.107490000 | 0.094020000 | 0.079054000 | 0.066456000 |
|  | 08 | 0.061513000 | 0.146210000 | 0.092460000 | 0.092288000 | 0.080263000 |
|  | 09 | 0.088812000 | 0.086709000 | 0.085642000 | 0.071777000 | 0.077659000 |
|  | 10 | 0.167590000 | 0.211510000 | 0.218230000 | 0.201760000 | 0.205950000 |
|  | 11 | 0.211060000 | 0.277870000 | 0.362420000 | 0.293900000 | 0.218420000 |
|  | 12 | 0.213480000 | 0.294330000 | 0.190770000 | 0.426480000 | 0.281360000 |
|  | 13 | 1.377409705 | 0.601161459 | 0.422058498 | 0.791999040 | 1.017708103 |
|  | 14 | 1.451750499 | 0.702876874 | 0.408431199 | 0.669159465 | 0.876698796 |
|  | 15 | 0.732725829 | 0.595123347 | 0.230971637 | 0.549265086 | 0.537188862 |
|  | 16 | 0.198177487 | 0.941917793 | 0.583462590 | 0.603275722 | 0.460262944 |
|  | Mean ± SEM | 0.407153377 ± | 0.412566441 ± | 0.302372400 ± | 0.380497988 ± | 0.382472510 ± |
|  |  | 0.111240367 | 0.077461521 | 0.060192083 | 0.067449434 | 0.079980652 |
| Concussion + | 01 | 0.186910000 | 0.235320000 | 0.143440000 | 0.124660000 | 0.138440000 |
| Ketamine | 02 | 0.048649000 | 0.081251000 | 0.050476000 | 0.068712000 | 0.058278000 |
|  | 03 | 0.185970000 | 1.748800000 | 0.396720000 | 0.332870000 | 0.298960000 |
|  | 04 | 0.242656569 | 0.949235847 | 0.228220331 | 0.285421703 | 0.229492640 |
|  | 05 | 0.213579039 | 1.075034375 | 0.313161737 | 0.286772997 | 0.326661300 |
|  | 06 | 0.412375780 | 0.537039308 | 0.282656420 | 0.365658393 | 0.457127717 |
|  | 07 | 0.208094925 | 0.271238602 | 0.182099307 | 0.162550574 | 0.214948958 |
|  | 08 | 0.297291842 | 0.949512195 | 0.163120269 | 0.163961312 | 0.273305299 |
|  | 09 | 0.299428091 | 1.080941968 | 0.317981497 | 0.272396972 | 0.184718251 |
|  | 10 | 0.223532380 | 1.077140454 | 0.191959630 | 0.164112700 | 0.315374264 |
|  | 11 | 0.224316594 | 0.335421079 | 0.201863740 | 0.214848679 | 0.180679126 |
|  | 12 | 0.251267173 | 0.201769396 | 0.148621283 | 0.201502202 | 0.163737438 |
|  | 13 | 0.497729410 | 0.356813577 | 0.248261514 | 0.187039092 | 0.245498494 |
|  | 14 | 0.072030034 | 0.739398149 | 0.378059896 | 0.478400459 | 0.390744332 |
|  | 15 | 0.105751706 | 0.310301726 | 0.272767421 | 0.213973255 | 0.294921341 |
|  | 16 | 0.219666716 | 0.506577481 | 0.386463772 | 0.259596448 | 0.175609739 |
|  | Mean ± SEM | 0.230578079 ± | 0.653487197 ± | 0.244117051 ± | 0.236404799 ± | 0.246781056 ± |
|  |  | 0.028350607 | 0.113956712 | 0.024660584 | 0.025207990 | 0.025047334 |

| **Condition** | **Case** | **Time points** |  |
| --- | --- | --- | --- |
|  |  | **50** | **60** |
| Sham + | 01 | 0.139940000 | 0.196110000 |
| Vehicle | 02 | 0.222970000 | 0.165620000 |
|  | 03 | 0.030626000 | 0.015514000 |
|  | 04 | 0.181620000 | 0.105160000 |
|  | 05 | 0.391160000 | 0.356000000 |
|  | 06 | 0.589310000 | 0.370210000 |
|  | 07 | 0.512350000 | 0.509810000 |
|  | 08 | 0.167070000 | 0.140660000 |
|  | 09 | 0.175040000 | 0.089246000 |
|  | 10 | 0.178610000 | 0.178980000 |
|  | 11 | 0.013950000 | 0.045198000 |
|  | 12 | 0.243992371 | 0.286117012 |
|  | 13 | 0.128373326 | 0.244891578 |
|  | 14 | 0.180721909 | 0.240208008 |
|  | 15 | 0.133926993 | 0.219828700 |
|  | 16 | 0.123662565 | 0.230120318 |
|  | Mean ± SEM | 0.213332698 ± | 0.212104601 ± |
|  |  | 0.039284904 | 0.031866508 |
| Sham + | 01 | 0.027928040 | 0.127803377 |
| MK-801 | 02 | 0.019570023 | 0.119444626 |
|  | 03 | 0.296960000 | 0.206100000 |
|  | 04 | 0.162840000 | 0.091971000 |
|  | 05 | 0.058912000 | 0.098976000 |
|  | 06 | 0.019868000 | 0.280740480 |
|  | 07 | 0.068894000 | 0.066218000 |
|  | 08 | 0.372470000 | 0.445610000 |
|  | 09 | 1.331900000 | 1.610200000 |
|  | 10 | 0.062190000 | 0.046781000 |
|  | 11 | 0.022354000 | 0.006514900 |
|  | 12 | 0.444030266 | 0.494417453 |
|  | 13 | 0.736542755 | 0.753162763 |
|  | 14 | 0.614559339 | 0.645965484 |
|  | 15 | 0.468487544 | 0.628884794 |
|  | 16 | 0.619431943 | 0.588592792 |
|  | Mean ± SEM | 0.332933619 ± | 0.388211417 ± |
|  |  | 0.091025104 | 0.102625090 |
| Concussion + | 01 | 0.297249731 | 0.181765360 |
| Vehicle | 02 | 0.216408987 | 0.150818513 |
|  | 03 | 0.233402385 | 0.184965306 |
|  | 04 | 0.197301940 | 0.263170305 |
|  | 05 | 0.203701212 | 0.194191737 |
|  | 06 | 0.191702961 | 0.153084904 |
|  | 07 | 0.287773051 | 0.212361478 |
|  | 08 | 0.233743084 | 0.142709402 |
|  | 09 | 0.216722200 | 0.158210487 |
|  | 10 | 0.229890280 | 0.129021944 |
|  | 11 | 0.285667407 | 0.237774300 |
|  | 12 | 0.265078083 | 0.131335824 |
|  | 13 | 0.268435161 | 0.266530110 |
|  | 14 | 0.176613393 | 0.209378583 |
|  | 15 | 0.242961427 | 0.269598422 |
|  | 16 | 0.225141614 | 0.271132578 |
|  | Mean ± SEM | 0.235737057 ± | 0.197253078 ± |
|  |  | 0.009082039 | 0.012887464 |
| Concussion + | 01 | 0.263176257 | 0.471855009 |
| MK-801 | 02 | 0.155490000 | 0.148050000 |
|  | 03 | 0.856170000 | 1.031000000 |
|  | 04 | 0.524290000 | 0.499150000 |
|  | 05 | 0.172080000 | 0.188050000 |
|  | 06 | 0.109280000 | 0.104550000 |
|  | 07 | 0.133220000 | 0.061710000 |
|  | 08 | 0.090401000 | 0.074741000 |
|  | 09 | 0.070129000 | 0.074059000 |
|  | 10 | 0.176890000 | 0.194400000 |
|  | 11 | 0.278430000 | 0.177620000 |
|  | 12 | 0.266390000 | 0.240060000 |
|  | 13 | 0.509444937 | 0.809116257 |
|  | 14 | 0.312966246 | 0.453633946 |
|  | 15 | 0.369238865 | 0.619543541 |
|  | 16 | 0.375018465 | 0.405679888 |
|  | Mean ± SEM | 0.291413423 ± | 0.347076165 ± |
|  |  | 0.051203199 | 0.071481137 |
| Concussion + | 01 | 0.120370000 | 0.127510000 |
| Ketamine | 02 | 0.043577000 | 0.043437000 |
|  | 03 | 0.366700000 | 0.301810000 |
|  | 04 | 0.197005050 | 0.231506901 |
|  | 05 | 0.228168493 | 0.245064431 |
|  | 06 | 0.445245528 | 0.507271035 |
|  | 07 | 0.193300490 | 0.163459142 |
|  | 08 | 0.193986543 | 0.153725820 |
|  | 09 | 0.180033642 | 0.260798991 |
|  | 10 | 0.235719092 | 0.177594617 |
|  | 11 | 0.201896970 | 0.134445800 |
|  | 12 | 0.084025613 | 0.172204525 |
|  | 13 | 0.222843191 | 0.107820991 |
|  | 14 | 0.416983814 | 0.298874690 |
|  | 15 | 0.192591907 | 0.227271351 |
|  | 16 | 0.158723965 | 0.146904961 |
|  | Mean ± SEM | 0.217573206 ± | 0.206231266 ± |
|  |  | 0.027323394 | 0.026734088 |
