# Supplementary material for: Intranasal delivery of the NMDA receptor antagonist MK-801 attenuates ultra-acute excitotoxic neurochemical responses after concussion in rats: comparative pharmacological evaluation against ketamine
Source: Front Pharmacol. 2026 Mar 16;17:1764201. doi: 10.3389/fphar.2026.1764201 (PMC13033605; doi:10.3389/fphar.2026.1764201)
Supplement: Supplementary file 3 [file Table7.docx]

*SUPPLEMENTARY TABLE 7:* Extracellular concentrations of taurine (µg/ml).

| **Condition** | **Case** | **Time points** |  |  |  |  |
| --- | --- | --- | --- | --- | --- | --- |
|  |  | **-50** | **-40** | **-30** | **-20** | **-10** |
| Sham + | 01 | 0.126340000 | 0.163510000 | 0.146400000 | 0.121450000 | 0.081045000 |
| Vehicle | 02 | 0.777890000 | 0.446530000 | 0.492200000 | 0.428480000 | 0.408260000 |
|  | 03 | 0.132350000 | 0.067567000 | 0.054524000 | 0.013241000 | 0.016104000 |
|  | 04 | 0.811920000 | 0.266890000 | 0.246900000 | 0.266780000 | 0.275670000 |
|  | 05 | 0.631970000 | 0.645980000 | 0.749090000 | 0.551370000 | 0.523630000 |
|  | 06 | 0.331630000 | 0.375970000 | 0.392410000 | 0.368990000 | 4.888600000 |
|  | 07 | 0.466880000 | 0.458720000 | 0.538350000 | 0.516940000 | 0.507850000 |
|  | 08 | 0.326970000 | 0.357520000 | 0.358880000 | 0.184410000 | 0.195320000 |
|  | 09 | 0.354240000 | 0.278250000 | 0.253900000 | 0.219810000 | 0.290240000 |
|  | 10 | 0.327720000 | 0.332580000 | 0.335420000 | 0.331630000 | 0.484520000 |
|  | 11 | 0.107070000 | 0.049743000 | 0.046395000 | 0.040280000 | 0.029937000 |
|  | 12 | 0.663088886 | 0.537483757 | 0.337540969 | 0.620802135 | 0.397628603 |
|  | 13 | 0.758910247 | 0.604278075 | 0.820190779 | 0.616129498 | 0.763520740 |
|  | 14 | 0.454227490 | 0.312241653 | 0.628934817 | 0.541740136 | 0.395057089 |
|  | 15 | 0.723196994 | 0.898077757 | 0.727518428 | 0.728848099 | 0.620161873 |
|  | 16 | 0.532316809 | 0.319583755 | 0.619656020 | 0.347564677 | 0.547015465 |
|  | Mean ± SEM | 0.480586459 ± | 0.377159643 ± | 0.415539287 ± | 0.358038253 ± | 0.359150698 ± |
|  |  | 0.068069679 | 0.062294023 | 0.069326554 | 0.061280292 | 0.060075560 |
| Sham + | 01 | 0.406573937 | 0.429170876 | 0.440876256 | 0.461822611 | 0.380882631 |
| MK-801 | 02 | 0.484449766 | 0.505372434 | 0.296537744 | 0.549818968 | 0.519094593 |
|  | 03 | 0.378850000 | 0.386480000 | 0.399470000 | 0.375650000 | 0.393280000 |
|  | 04 | 0.703500000 | 0.600400000 | 0.548170000 | 0.386930000 | 0.417640000 |
|  | 05 | 0.291060000 | 0.259430000 | 0.268280000 | 0.318230000 | 0.385020000 |
|  | 06 | 0.688150000 | 0.622110000 | 0.643090000 | 0.578300000 | 0.612540000 |
|  | 07 | 0.316190000 | 0.382530000 | 0.303930000 | 0.277220000 | 0.282690000 |
|  | 08 | 0.675800000 | 0.570030000 | 0.578550000 | 0.544050000 | 0.512890000 |
|  | 09 | 2.088900000 | 4.193000000 | 4.270500000 | 4.077000000 | 4.728200000 |
|  | 10 | 0.596960000 | 1.024100000 | 0.683170000 | 0.449820000 | 0.529110000 |
|  | 11 | 0.462730000 | 0.580910000 | 0.550350000 | 0.694630000 | 0.332900000 |
|  | 12 | 0.073192290 | 0.159811905 | 0.589032695 | 0.065413902 | 0.069456436 |
|  | 13 | 0.559583189 | 0.579016248 | 0.446968575 | 0.608252259 | 0.572382175 |
|  | 14 | 0.646081288 | 0.566933347 | 0.609898431 | 0.657382258 | 0.661588227 |
|  | 15 | 0.643077024 | 0.701022273 | 0.545590729 | 0.672255420 | 0.650386027 |
|  | 16 | 0.624310666 | 0.553648740 | 0.496682964 | 0.770762342 | 0.463784220 |
|  | Mean ± SEM | 0.602463010 ± | 0.757122864 ± | 0.729443587 ± | 0.717971110 ± | 0.719490269 ± |
|  |  | 0.108382071 | 0.234084405 | 0.238147843 | 0.228458714 | 0.269894084 |
| Concussion + | 01 | 0.328416607 | 0.387515552 | 0.689591382 | 0.581019580 | 0.551191801 |
| Vehicle | 02 | 0.681422304 | 0.379002685 | 0.472120359 | 0.481091612 | 0.552124943 |
|  | 03 | 0.572326632 | 0.345622422 | 0.692603628 | 0.697351189 | 0.440409927 |
|  | 04 | 0.415820837 | 0.304204047 | 0.513440508 | 0.687135747 | 0.466505140 |
|  | 05 | 0.689685001 | 0.494352864 | 0.506528480 | 0.441700669 | 0.336641097 |
|  | 06 | 0.487905990 | 0.513122246 | 0.756895708 | 0.376236331 | 0.462036886 |
|  | 07 | 0.400048964 | 0.474065611 | 0.712648931 | 0.560927044 | 0.616631304 |
|  | 08 | 0.443153256 | 0.306251020 | 0.410184430 | 0.715227681 | 0.350040803 |
|  | 09 | 0.383345392 | 0.646862461 | 0.666299415 | 0.423452608 | 0.565168059 |
|  | 10 | 0.736729593 | 0.577451819 | 0.617855029 | 0.389462606 | 0.337120963 |
|  | 11 | 0.531901598 | 0.486006051 | 0.289416563 | 0.432924423 | 0.435522594 |
|  | 12 | 0.557225548 | 0.416200750 | 0.566878248 | 0.568637769 | 0.517529435 |
|  | 13 | 0.536565459 | 0.605550114 | 0.548302318 | 0.362259223 | 0.644123408 |
|  | 14 | 0.328795299 | 0.585977800 | 0.447551420 | 0.669980411 | 0.551730330 |
|  | 15 | 0.452138426 | 0.441250408 | 0.394547829 | 0.442017630 | 0.360463598 |
|  | 16 | 0.372428991 | 0.510888018 | 0.435634998 | 0.435161606 | 0.507394711 |
|  | Mean ± SEM | 0.494869368 ± | 0.467145242 ± | 0.545031203 ± | 0.516536633 ± | 0.480914687 ± |
|  |  | 0.032064667 | 0.026417683 | 0.033639561 | 0.030821060 | 0.024716240 |
| Concussion + | 01 | 0.672634450 | 0.691367076 | 0.641419888 | 0.601942361 | 0.732526359 |
| MK-801 | 02 | 0.274470000 | 0.336080000 | 0.255860000 | 0.329320000 | 0.401270000 |
|  | 03 | 3.335800000 | 3.080700000 | 3.045300000 | 2.909000000 | 2.995800000 |
|  | 04 | 0.337580000 | 0.249120000 | 0.330480000 | 0.238350000 | 0.259630000 |
|  | 05 | 0.415020000 | 0.392950000 | 0.241350000 | 0.276340000 | 0.289220000 |
|  | 06 | 0.985350000 | 0.583140000 | 0.414860000 | 0.432380000 | 0.390850000 |
|  | 07 | 0.390910000 | 0.164970000 | 0.133420000 | 0.227150000 | 0.246530000 |
|  | 08 | 0.217570000 | 0.195390000 | 0.233300000 | 0.198780000 | 0.195130000 |
|  | 09 | 0.276730000 | 0.381560000 | 0.349510000 | 0.362100000 | 0.257310000 |
|  | 10 | 0.952730000 | 0.531220000 | 0.185760000 | 0.499170000 | 0.488970000 |
|  | 11 | 0.496540000 | 0.668010000 | 0.442470000 | 0.436780000 | 0.458380000 |
|  | 12 | 0.662490000 | 0.656180000 | 0.471450000 | 0.438810000 | 0.530330000 |
|  | 13 | 0.572556250 | 0.610733465 | 0.531235130 | 0.799622731 | 0.594155734 |
|  | 14 | 0.606331996 | 0.723922575 | 0.535619604 | 0.431139963 | 0.581486643 |
|  | 15 | 0.564331113 | 0.632103528 | 0.620394603 | 0.536681735 | 0.692695942 |
|  | 16 | 0.745114200 | 0.569599279 | 0.553905241 | 0.715383047 | 0.647168785 |
|  | Mean ± SEM | 0.719134876 ± | 0.654190370 ± | 0.561645904 ± | 0.589559365 ± | 0.610090841 ± |
|  |  | 0.183450262 | 0.168099361 | 0.170182645 | 0.160360498 | 0.164648543 |
| Concussion + | 01 | 0.352670000 | 0.365910000 | 0.297590000 | 0.231280000 | 0.296250000 |
| Ketamine | 02 | 0.234750000 | 0.269140000 | 0.238900000 | 0.226500000 | 0.218580000 |
|  | 03 | 0.792470000 | 0.771160000 | 0.657150000 | 0.269130000 | 0.309900000 |
|  | 04 | 0.544093099 | 0.509679895 | 0.537489769 | 0.468344534 | 0.465589191 |
|  | 05 | 0.429473035 | 0.342345045 | 0.358860657 | 0.610206146 | 0.461824592 |
|  | 06 | 0.628850151 | 0.616443521 | 0.823156159 | 2.567095501 | 1.872349905 |
|  | 07 | 0.700922615 | 0.713664130 | 0.748862579 | 0.765059115 | 0.721052039 |
|  | 08 | 0.354490539 | 0.241923544 | 0.383731604 | 0.565026206 | 0.331734328 |
|  | 09 | 0.533208966 | 0.532824021 | 0.311998460 | 0.345888484 | 0.366305410 |
|  | 10 | 0.368230138 | 0.506884605 | 0.453673270 | 0.457730005 | 0.423543869 |
|  | 11 | 1.508421503 | 1.380702360 | 1.434401010 | 1.486880842 | 1.553097428 |
|  | 12 | 0.799403764 | 0.724615219 | 0.729646532 | 0.736624659 | 0.732830210 |
|  | 13 | 1.715685986 | 1.678204896 | 1.552852701 | 1.636399351 | 1.777474176 |
|  | 14 | 1.017130802 | 0.886722628 | 1.001338431 | 0.976236897 | 0.899610656 |
|  | 15 | 1.427671186 | 1.314292565 | 1.306741656 | 1.367877444 | 1.303421275 |
|  | 16 | 0.825899851 | 0.883539847 | 0.836621345 | 0.795098139 | 0.916764884 |
|  | Mean ± SEM | 0.764585727 ± | 0.733628267 ± | 0.729563386 ± | 0.844086083 ± | 0.790645498 ± |
|  |  | 0.111424490 | 0.104030505 | 0.103665624 | 0.159170602 | 0.137933665 |

| **Condition** | **Case** | **Time points** |  |  |  |  |
| --- | --- | --- | --- | --- | --- | --- |
|  |  | **0** | **10** | **20** | **30** | **40** |
| Sham + | 01 | 0.083951000 | 0.312720000 | 0.287270000 | 0.304510000 | 0.207620000 |
| Vehicle | 02 | 0.390620000 | 0.609670000 | 0.592190000 | 0.513110000 | 0.500870000 |
|  | 03 | 0.011627000 | 0.016034000 | 0.014842000 | 0.014668000 | 0.017558000 |
|  | 04 | 0.251310000 | 0.281570000 | 0.284470000 | 0.307360000 | 0.250250000 |
|  | 05 | 0.411850000 | 0.606060000 | 0.784260000 | 0.759280000 | 0.819770000 |
|  | 06 | 4.530200000 | 4.890400000 | 4.362600000 | 5.023600000 | 4.633800000 |
|  | 07 | 0.514630000 | 1.507400000 | 1.423600000 | 1.435500000 | 1.494400000 |
|  | 08 | 0.460950000 | 0.189660000 | 0.184110000 | 0.188550000 | 0.169030000 |
|  | 09 | 0.277610000 | 0.291410000 | 0.269590000 | 0.399840000 | 0.445390000 |
|  | 10 | 0.334000000 | 0.760390000 | 0.951570000 | 0.641870000 | 0.597970000 |
|  | 11 | 0.031936000 | 0.008770800 | 0.011800000 | 0.012490000 | 0.003709500 |
|  | 12 | 0.395268896 | 0.478261576 | 0.550651890 | 0.286501585 | 0.352482411 |
|  | 13 | 0.657884087 | 0.660196560 | 0.685922821 | 0.268998410 | 0.489825119 |
|  | 14 | 0.631695332 | 0.367105073 | 0.606272583 | 0.531680879 | 0.400838271 |
|  | 15 | 0.757898540 | 0.346018211 | 0.321578263 | 0.339962422 | 0.673464373 |
|  | 16 | 0.591516115 | 0.849934962 | 0.548518572 | 0.540887411 | 0.403237462 |
|  | Mean ± SEM | 0.377722641 ± | 0.412700084 ± | 0.435217581 ± | 0.364979193 ± | 0.380858224 ± |
|  |  | 0.062441629 | 0.069237533 | 0.075424757 | 0.058134933 | 0.063059148 |
| Sham + | 01 | 0.334955359 | 0.333433516 | 0.328850633 | 0.346488416 | 0.376876196 |
| MK-801 | 02 | 0.408595463 | 0.644244396 | 0.226943802 | 0.048372701 | 0.000000000 |
|  | 03 | 0.432750000 | 0.617940000 | 1.623500000 | 2.007400000 | 1.143500000 |
|  | 04 | 0.415990000 | 0.362640000 | 0.500420000 | 0.441370000 | 0.430690000 |
|  | 05 | 0.122730000 | 0.222760000 | 0.219250000 | 0.260600000 | 0.241460000 |
|  | 06 | 0.598020000 | 0.001640800 | 0.001447600 | 0.001234100 | 0.001437300 |
|  | 07 | 0.226940000 | 0.287650000 | 0.265720000 | 0.248120000 | 0.270210000 |
|  | 08 | 0.595920000 | 0.752580000 | 0.683150000 | 0.615620000 | 0.527250000 |
|  | 09 | 4.579600000 | 4.825300000 | 4.969600000 | 4.554000000 | 4.816800000 |
|  | 10 | 0.359720000 | 0.198440000 | 0.191040000 | 0.181870000 | 0.187140000 |
|  | 11 | 0.368520000 | 0.526120000 | 0.184090000 | 0.000238900 | 0.000572000 |
|  | 12 | 0.455726779 | 0.665440811 | 0.083378396 | 0.077551967 | 0.585659461 |
|  | 13 | 0.503070110 | 0.615092103 | 0.367030471 | 0.425732958 | 0.391616047 |
|  | 14 | 0.589131974 | 0.599815629 | 0.486427313 | 0.505012593 | 0.527721534 |
|  | 15 | 0.510000494 | 0.601922729 | 0.379022832 | 0.391476122 | 0.514535697 |
|  | 16 | 0.418358108 | 0.685523565 | 0.473776483 | 0.391574893 | 0.367540784 |
|  | Mean ± SEM | 0.682501768 ± | 0.746283972 ± | 0.686477971 ± | 0.656041416 ± | 0.648938064 ± |
|  |  | 0.261778759 | 0.277189593 | 0.300033798 | 0.284970423 | 0.286631081 |
| Concussion + | 01 | 0.571917360 | 1.212510641 | 1.069134307 | 0.670028158 | 0.761508742 |
| Vehicle | 02 | 0.560572327 | 2.310310392 | 0.974592365 | 0.369949578 | 0.422794840 |
|  | 03 | 0.560441359 | 1.331641674 | 0.833769891 | 0.595409600 | 0.582476590 |
|  | 04 | 0.552223168 | 2.394440443 | 0.552959859 | 0.734382162 | 0.990848667 |
|  | 05 | 0.387122572 | 1.897715032 | 0.860176269 | 0.394173331 | 0.660421087 |
|  | 06 | 0.550334585 | 2.771144116 | 0.642125020 | 0.536673739 | 0.525559001 |
|  | 07 | 0.535759752 | 1.574032969 | 0.534029705 | 0.664778848 | 0.865986617 |
|  | 08 | 0.555687939 | 1.483890974 | 0.875208095 | 0.661302432 | 0.776758609 |
|  | 09 | 0.464924686 | 0.978458199 | 0.489886864 | 0.834259686 | 0.802900743 |
|  | 10 | 0.554166941 | 1.905758732 | 0.715664671 | 0.668946918 | 0.372788265 |
|  | 11 | 0.483753207 | 2.811155693 | 0.899740183 | 0.817634677 | 0.788429915 |
|  | 12 | 0.420624219 | 1.968953496 | 0.882753404 | 0.711619417 | 0.995658752 |
|  | 13 | 0.530117532 | 2.918152138 | 0.449330722 | 0.620617042 | 0.737055175 |
|  | 14 | 0.565915769 | 2.180607248 | 0.760316683 | 0.384443356 | 0.934688214 |
|  | 15 | 0.465556644 | 1.241952334 | 0.587626510 | 0.655239961 | 0.401207966 |
|  | 16 | 0.433790402 | 1.753232125 | 0.816731962 | 0.787724453 | 0.416323865 |
|  | Mean ± SEM | 0.512056779 ± | 1.920872263 ± | 0.746502907 ± | 0.631698960 ± | 0.689712941 ± |
|  |  | 0.015014061 | 0.151299928 | 0.046288998 | 0.036446044 | 0.053302112 |
| Concussion + | 01 | 0.570183415 | 0.778400392 | 0.523026192 | 0.550235647 | 0.476510564 |
| MK-801 | 02 | 0.493100000 | 0.366650000 | 0.402630000 | 0.445380000 | 0.398160000 |
|  | 03 | 2.296200000 | 3.675600000 | 3.164100000 | 2.670000000 | 2.572800000 |
|  | 04 | 0.341670000 | 0.507100000 | 0.359500000 | 0.392600000 | 0.350170000 |
|  | 05 | 0.271580000 | 2.230500000 | 0.741410000 | 0.850050000 | 0.524470000 |
|  | 06 | 0.386410000 | 0.514370000 | 0.566300000 | 0.505490000 | 0.550740000 |
|  | 07 | 0.201380000 | 0.265190000 | 0.234050000 | 0.205120000 | 0.204310000 |
|  | 08 | 0.218700000 | 0.560260000 | 0.337790000 | 0.313330000 | 0.306280000 |
|  | 09 | 0.365100000 | 0.298660000 | 0.300500000 | 0.321490000 | 0.366070000 |
|  | 10 | 0.429780000 | 0.543110000 | 0.550740000 | 0.481970000 | 0.500160000 |
|  | 11 | 0.494630000 | 0.824090000 | 0.960200000 | 1.210700000 | 0.872570000 |
|  | 12 | 0.496410000 | 0.967610000 | 0.540610000 | 0.495020000 | 0.514900000 |
|  | 13 | 0.617998437 | 0.653244171 | 0.499192781 | 0.482980423 | 0.548942968 |
|  | 14 | 0.509091836 | 0.708789341 | 0.448941268 | 0.434079940 | 0.541287132 |
|  | 15 | 0.619510910 | 0.617930460 | 0.429406567 | 0.485036707 | 0.379231527 |
|  | 16 | 0.442262593 | 0.699196180 | 0.383072191 | 0.407365237 | 0.418674801 |
|  | Mean ± SEM | 0.547125449 ± | 0.888168784 ± | 0.652591812 ± | 0.640677997 ± | 0.595329812 ± |
|  |  | 0.120916957 | 0.217076519 | 0.173101839 | 0.147258452 | 0.136852529 |
| Concussion + | 01 | 0.275530000 | 0.392570000 | 0.378970000 | 0.287700000 | 0.305720000 |
| Ketamine | 02 | 0.257840000 | 0.321910000 | 0.176060000 | 0.236300000 | 0.221790000 |
|  | 03 | 0.211960000 | 0.793370000 | 0.364590000 | 0.298660000 | 0.305350000 |
|  | 04 | 0.489095458 | 2.176287010 | 0.827627685 | 0.699427045 | 0.422634304 |
|  | 05 | 0.305302603 | 1.958621369 | 0.688436539 | 0.643940033 | 0.409012105 |
|  | 06 | 2.118490648 | 3.512360446 | 1.054046847 | 1.167859277 | 1.636313289 |
|  | 07 | 0.835123528 | 1.588618073 | 0.914469056 | 0.726751547 | 0.735413807 |
|  | 08 | 0.362337509 | 4.248215925 | 0.635619318 | 0.522785822 | 0.481641053 |
|  | 09 | 0.431701756 | 2.404696337 | 0.628201712 | 0.383168991 | 0.379334340 |
|  | 10 | 0.487341210 | 2.370347339 | 0.531225016 | 0.330905215 | 0.561487667 |
|  | 11 | 0.830022745 | 2.511156361 | 1.696103309 | 0.985582567 | 0.938085623 |
|  | 12 | 0.759610845 | 1.045410142 | 0.653447712 | 0.620023097 | 0.681516720 |
|  | 13 | 1.614317333 | 2.593099575 | 2.157418561 | 1.703278680 | 1.930096095 |
|  | 14 | 0.361920911 | 4.259914495 | 1.924680636 | 2.191636119 | 1.799518863 |
|  | 15 | 0.572340265 | 1.921294967 | 1.449640861 | 1.107407332 | 0.990796799 |
|  | 16 | 0.739182803 | 4.263013752 | 2.594717761 | 1.042836736 | 0.822880627 |
|  | Mean ± SEM | 0.665757351 ± | 2.272555362 ± | 1.042203438 ± | 0.809266404 ± | 0.788849456 ± |
|  |  | 0.129832138 | 0.324491840 | 0.178093994 | 0.136150086 | 0.136954747 |

| **Condition** | **Case** | **Time points** |  |
| --- | --- | --- | --- |
|  |  | **50** | **60** |
| Sham + | 01 | 0.329150000 | 0.404790000 |
| Vehicle | 02 | 0.900970000 | 0.872270000 |
|  | 03 | 0.015231000 | 0.012979000 |
|  | 04 | 0.283410000 | 0.286730000 |
|  | 05 | 0.764770000 | 0.817080000 |
|  | 06 | 3.719000000 | 1.433600000 |
|  | 07 | 1.402800000 | 1.376500000 |
|  | 08 | 0.179520000 | 0.165400000 |
|  | 09 | 0.428910000 | 0.287540000 |
|  | 10 | 0.660050000 | 0.708200000 |
|  | 11 | 0.005626000 | 0.004746400 |
|  | 12 | 0.348331276 | 0.445343426 |
|  | 13 | 0.364633618 | 0.312819772 |
|  | 14 | 0.553577107 | 0.467249603 |
|  | 15 | 0.430510189 | 0.387425929 |
|  | 16 | 0.489940743 | 0.457956352 |
|  | Mean ± SEM | 0.411044995 ± | 0.402180749 ± |
|  |  | 0.068665097 | 0.070170883 |
| Sham + | 01 | 0.177336465 | 0.431883138 |
| MK-801 | 02 | 0.130378304 | 0.187712483 |
|  | 03 | 0.727640000 | 0.529480000 |
|  | 04 | 0.355570000 | 0.240020000 |
|  | 05 | 0.111780000 | 0.199970000 |
|  | 06 | 0.001732000 | 0.519150250 |
|  | 07 | 0.292450000 | 0.294760000 |
|  | 08 | 0.598260000 | 0.695040000 |
|  | 09 | 4.588200000 | 3.749200000 |
|  | 10 | 0.134510000 | 0.143540000 |
|  | 11 | 0.000925620 | 0.000713050 |
|  | 12 | 0.509669354 | 0.454443589 |
|  | 13 | 0.483184355 | 0.522182165 |
|  | 14 | 0.457150147 | 0.482550579 |
|  | 15 | 0.494172552 | 0.472607701 |
|  | 16 | 0.496888735 | 0.424531253 |
|  | Mean ± SEM | 0.597490471 ± | 0.584236513 ± |
|  |  | 0.271656592 | 0.215741047 |
| Concussion + | 01 | 0.635518303 | 0.600975706 |
| Vehicle | 02 | 0.716898042 | 0.639087159 |
|  | 03 | 0.702475280 | 0.453752210 |
|  | 04 | 0.726442276 | 0.628200511 |
|  | 05 | 0.685376204 | 0.366737392 |
|  | 06 | 0.485555737 | 0.337816223 |
|  | 07 | 0.613921985 | 0.521135956 |
|  | 08 | 0.674016648 | 0.713546597 |
|  | 09 | 0.395464711 | 0.492715254 |
|  | 10 | 0.680096034 | 0.745017431 |
|  | 11 | 0.454104453 | 0.514240610 |
|  | 12 | 0.688531869 | 0.582549497 |
|  | 13 | 0.581978452 | 0.393552073 |
|  | 14 | 0.444515181 | 0.602938296 |
|  | 15 | 0.733349657 | 0.644221352 |
|  | 16 | 0.619392752 | 0.660610513 |
|  | Mean ± SEM | 0.614852349 ± | 0.556068549 ± |
|  |  | 0.027687009 | 0.030520132 |
| Concussion + | 01 | 0.547659547 | 0.520819495 |
| MK-801 | 02 | 0.419060000 | 0.437510000 |
|  | 03 | 2.203600000 | 2.405500000 |
|  | 04 | 0.380490000 | 0.374580000 |
|  | 05 | 0.537460000 | 0.533080000 |
|  | 06 | 0.513070000 | 0.483640000 |
|  | 07 | 0.226270000 | 0.202640000 |
|  | 08 | 0.299830000 | 0.260820000 |
|  | 09 | 0.337260000 | 0.305860000 |
|  | 10 | 0.418070000 | 0.352230000 |
|  | 11 | 0.860210000 | 0.624180000 |
|  | 12 | 0.605620000 | 0.526740000 |
|  | 13 | 0.421869689 | 0.451473387 |
|  | 14 | 0.495173680 | 0.454770240 |
|  | 15 | 0.495343620 | 0.546087961 |
|  | 16 | 0.498835905 | 0.466521650 |
|  | Mean ± SEM | 0.578738903 ± | 0.559153296 ± |
|  |  | 0.114072087 | 0.126248969 |
| Concussion + | 01 | 0.318070000 | 0.325360000 |
| Ketamine | 02 | 0.221470000 | 0.252860000 |
|  | 03 | 0.286720000 | 0.305790000 |
|  | 04 | 0.509028536 | 0.419745078 |
|  | 05 | 0.336435173 | 0.536703826 |
|  | 06 | 1.775977762 | 1.748663691 |
|  | 07 | 0.630004024 | 0.677592233 |
|  | 08 | 0.377305973 | 0.547170649 |
|  | 09 | 0.540182405 | 0.437786859 |
|  | 10 | 0.529729650 | 0.510497172 |
|  | 11 | 0.793115657 | 0.715071893 |
|  | 12 | 0.240374391 | 0.617703327 |
|  | 13 | 1.517440049 | 0.961714524 |
|  | 14 | 1.578600541 | 1.452518915 |
|  | 15 | 1.079256423 | 1.255979693 |
|  | 16 | 0.870866096 | 0.899522529 |
|  | Mean ± SEM | 0.725286042 ± | 0.729042524 ± |
|  |  | 0.126795076 | 0.108134312 |
