# Supplementary material for: Intranasal delivery of the NMDA receptor antagonist MK-801 attenuates ultra-acute excitotoxic neurochemical responses after concussion in rats: comparative pharmacological evaluation against ketamine
Source: Front Pharmacol. 2026 Mar 16;17:1764201. doi: 10.3389/fphar.2026.1764201 (PMC13033605; doi:10.3389/fphar.2026.1764201)
Supplement: Supplementary file 4 [file Table9.docx]

*SUPPLEMENTARY TABLE 9:* Extracellular concentrations of glutamine (µg/ml).

| **Condition** | **Case** | **Time points** |  |  |  |  |
| --- | --- | --- | --- | --- | --- | --- |
|  |  | **-50** | **-40** | **-30** | **-20** | **-10** |
| Sham + | 01 | 2.420900000 | 3.019600000 | 2.896300000 | 2.331400000 | 1.524400000 |
| Vehicle | 02 | 5.541700000 | 6.872200000 | 7.296300000 | 7.658700000 | 7.843500000 |
|  | 03 | 2.632900000 | 1.002800000 | 1.065700000 | 0.192850000 | 0.220970000 |
|  | 04 | 14.445000000 | 5.470200000 | 5.207100000 | 4.929900000 | 4.919700000 |
|  | 05 | 8.383800000 | 8.861700000 | 9.877200000 | 6.959600000 | 6.966600000 |
|  | 06 | 6.324500000 | 7.070600000 | 7.405300000 | 7.021400000 | 6.453100000 |
|  | 07 | 6.355400000 | 5.977100000 | 6.139700000 | 6.476500000 | 6.534700000 |
|  | 08 | 3.470900000 | 3.360000000 | 3.540400000 | 3.561100000 | 3.580200000 |
|  | 09 | 7.417700000 | 6.315700000 | 5.713900000 | 6.738000000 | 7.187500000 |
|  | 10 | 3.694400000 | 3.753800000 | 4.025700000 | 7.711300000 | 3.801700000 |
|  | 11 | 0.150110000 | 0.069269000 | 0.062355000 | 0.077795000 | 0.038936000 |
|  | 12 | 3.067704579 | 2.716770869 | 3.106860590 | 2.339949619 | 2.604148482 |
|  | 13 | 4.461128665 | 3.774524212 | 2.894977588 | 3.558509075 | 3.283011243 |
|  | 14 | 3.164596958 | 3.543537365 | 3.240667940 | 3.571294731 | 3.155779264 |
|  | 15 | 3.339628187 | 3.713535161 | 3.200529062 | 3.424774047 | 2.914762290 |
|  | 16 | 4.386692630 | 2.593816592 | 2.987673598 | 2.981464472 | 3.099860387 |
|  | Mean ± SEM | 4.953566314 ± | 4.257197075 ± | 4.291291486 ± | 4.345908559 ± | 4.008054229 ± |
|  |  | 0.818277344 | 0.582806638 | 0.627239046 | 0.629105190 | 0.607244456 |
| Sham + | 01 | 2.563013015 | 2.723451088 | 2.765148858 | 2.925533827 | 2.709719380 |
| MK-801 | 02 | 0.713924099 | 0.947776071 | 0.542609896 | 1.032835220 | 0.973435839 |
|  | 03 | 6.851500000 | 6.900400000 | 7.423800000 | 6.825300000 | 7.087100000 |
|  | 04 | 0.988010000 | 0.912930000 | 1.116700000 | 0.998500000 | 1.182700000 |
|  | 05 | 4.234000000 | 4.246500000 | 4.322500000 | 5.416000000 | 6.414900000 |
|  | 06 | 3.277500000 | 3.705600000 | 4.278500000 | 3.984500000 | 4.005900000 |
|  | 07 | 1.867900000 | 3.086900000 | 3.095500000 | 3.375000000 | 3.997300000 |
|  | 08 | 3.563600000 | 3.373600000 | 3.502700000 | 3.596700000 | 3.546000000 |
|  | 09 | 3.981400000 | 3.399600000 | 3.453500000 | 3.436600000 | 2.245400000 |
|  | 10 | 2.716900000 | 3.354800000 | 2.133300000 | 2.822700000 | 2.371100000 |
|  | 11 | 2.895200000 | 3.670600000 | 3.883400000 | 5.534600000 | 2.619600000 |
|  | 12 | 2.096105869 | 0.080962055 | 0.576725583 | 1.374964130 | 1.305624562 |
|  | 13 | 1.229094784 | 0.726982697 | 0.641186320 | 0.908584078 | 0.674117110 |
|  | 14 | 0.974318805 | 0.641833266 | 0.980991222 | 0.632522326 | 0.863564289 |
|  | 15 | 1.051482900 | 0.559227217 | 0.692973666 | 0.870547493 | 0.762247564 |
|  | 16 | 1.126947179 | 0.605794601 | 1.060381571 | 0.687988380 | 0.485177339 |
|  | Mean ± SEM | 2.508181041 ± | 2.433559812 ± | 2.529369820 ± | 2.776429716 ± | 2.577742880 ± |
|  |  | 0.408472636 | 0.468131808 | 0.481519437 | 0.492287893 | 0.502485407 |
| Concussion + | 01 | 2.618639398 | 2.857668374 | 2.767615220 | 2.208887016 | 2.423526933 |
| Vehicle | 02 | 3.533269592 | 1.863388864 | 2.960653400 | 2.447073313 | 2.342791858 |
|  | 03 | 2.849452259 | 2.991816296 | 2.741832501 | 2.302910482 | 1.787580217 |
|  | 04 | 2.707590588 | 2.644016983 | 2.410805730 | 2.710215855 | 1.906462695 |
|  | 05 | 3.238666970 | 2.127088944 | 2.690966025 | 3.289337054 | 1.910390369 |
|  | 06 | 2.350261219 | 2.097056819 | 2.844485187 | 2.997322906 | 2.021319402 |
|  | 07 | 3.585894149 | 3.212415225 | 2.655628387 | 2.586721615 | 2.601323945 |
|  | 08 | 2.023996495 | 3.138803258 | 2.342116364 | 2.718061460 | 2.103027550 |
|  | 09 | 3.113704476 | 2.054352075 | 2.230617630 | 2.277884553 | 1.660140726 |
|  | 10 | 2.558603166 | 2.225698743 | 2.547983582 | 2.583116164 | 2.021157730 |
|  | 11 | 3.374812691 | 2.735715682 | 3.027851977 | 2.748729559 | 2.870936217 |
|  | 12 | 1.992931136 | 2.088784286 | 2.177992052 | 3.442781289 | 2.897077986 |
|  | 13 | 2.463245808 | 3.065790329 | 2.745239178 | 2.719923957 | 1.947013519 |
|  | 14 | 2.760204082 | 2.446038607 | 2.550679189 | 2.907237099 | 1.783536982 |
|  | 15 | 3.182796048 | 1.901858833 | 2.873391395 | 2.545934616 | 3.259294164 |
|  | 16 | 2.966268036 | 2.264542441 | 2.703399194 | 2.072679709 | 2.144075783 |
|  | Mean ± SEM | 2.832521007 ± | 2.482189735 ± | 2.641953563 ± | 2.659926040 ± | 2.229978505 ± |
|  |  | 0.122589450 | 0.116234808 | 0.062343792 | 0.093378348 | 0.115842470 |
| Concussion + | 01 | 0.394371718 | 0.467019526 | 0.438244759 | 0.455062160 | 0.442550491 |
| MK-801 | 02 | 4.170000000 | 4.955400000 | 3.803600000 | 5.120300000 | 5.015800000 |
|  | 03 | 2.689000000 | 2.439300000 | 2.375400000 | 2.483800000 | 2.357900000 |
|  | 04 | 3.408400000 | 3.060000000 | 3.755100000 | 2.973000000 | 3.049400000 |
|  | 05 | 2.496500000 | 1.886100000 | 2.063700000 | 2.492600000 | 2.460900000 |
|  | 06 | 4.114400000 | 3.939600000 | 3.783100000 | 4.151000000 | 3.771300000 |
|  | 07 | 1.600000000 | 0.963710000 | 0.984120000 | 2.625200000 | 3.106700000 |
|  | 08 | 4.812900000 | 4.100200000 | 5.077100000 | 4.457000000 | 4.237900000 |
|  | 09 | 6.572100000 | 7.614500000 | 7.298400000 | 7.009100000 | 5.021500000 |
|  | 10 | 4.948100000 | 2.814000000 | 1.066800000 | 3.778100000 | 3.693600000 |
|  | 11 | 5.441900000 | 6.203900000 | 5.420800000 | 5.283300000 | 5.031100000 |
|  | 12 | 4.850800000 | 4.625900000 | 3.497200000 | 3.417100000 | 3.805000000 |
|  | 13 | 0.634567396 | 1.202252894 | 0.823286010 | 0.625255530 | 1.115097771 |
|  | 14 | 0.552445334 | 1.032644401 | 1.110367822 | 0.589509523 | 0.916334184 |
|  | 15 | 0.907254938 | 0.546171632 | 0.653776205 | 0.966974947 | 1.167388025 |
|  | 16 | 0.634264285 | 0.698333592 | 0.838272406 | 0.625290776 | 0.957726523 |
|  | Mean ± SEM | 3.014187729 ± | 2.909314503 ± | 2.686829200 ± | 2.940787059 ± | 2.884387312 ± |
|  |  | 0.510987225 | 0.542235255 | 0.513464864 | 0.492938119 | 0.397905905 |
| Concussion + | 01 | 4.672700000 | 4.636400000 | 4.527400000 | 3.300100000 | 4.958800000 |
| Ketamine | 02 | 4.087500000 | 4.912200000 | 4.457700000 | 4.947800000 | 4.637400000 |
|  | 03 | 4.187800000 | 4.618600000 | 3.936400000 | 2.197500000 | 2.740500000 |
|  | 04 | 2.755385151 | 2.601115856 | 2.736326678 | 2.493001510 | 2.020139649 |
|  | 05 | 2.840328887 | 2.653540304 | 2.526529305 | 2.475699717 | 1.961621707 |
|  | 06 | 5.095827253 | 5.277280145 | 5.882890282 | 6.289346337 | 5.052868969 |
|  | 07 | 4.677872249 | 4.738296187 | 5.015995915 | 4.911954381 | 4.682061867 |
|  | 08 | 2.798586515 | 2.161933387 | 2.514476036 | 2.605767669 | 2.246628757 |
|  | 09 | 2.798342811 | 2.345507717 | 2.684841592 | 2.726173842 | 2.126287571 |
|  | 10 | 2.920243704 | 2.489666937 | 2.770333063 | 2.659398863 | 2.040406174 |
|  | 11 | 7.890991862 | 7.297578540 | 7.606925142 | 7.950493202 | 8.359384462 |
|  | 12 | 4.862146809 | 4.489276693 | 4.538915287 | 4.543138958 | 4.404498066 |
|  | 13 | 8.097548526 | 7.924197370 | 7.354080019 | 7.612014819 | 8.833031163 |
|  | 14 | 7.484583817 | 6.754308480 | 7.504466860 | 7.223129512 | 6.683946685 |
|  | 15 | 4.732217890 | 4.503163540 | 4.548043312 | 4.947230658 | 4.632129521 |
|  | 16 | 5.221935112 | 5.507287012 | 5.189077029 | 4.916450447 | 5.385906114 |
|  | Mean ± SEM | 4.695250662 ± | 4.556897011 ± | 4.612150033 ± | 4.487449995 ± | 4.422850669 ± |
|  |  | 0.448207474 | 0.445463106 | 0.441756867 | 0.491342724 | 0.548386649 |

| **Condition** | **Case** | **Time points** |  |  |  |  |
| --- | --- | --- | --- | --- | --- | --- |
|  |  | **0** | **10** | **20** | **30** | **40** |
| Sham + | 01 | 1.968800000 | 7.154400000 | 8.065800000 | 7.832500000 | 4.850400000 |
| Vehicle | 02 | 7.757300000 | 8.752700000 | 7.411100000 | 7.986300000 | 7.861100000 |
|  | 03 | 0.175340000 | 0.206720000 | 0.221200000 | 0.206230000 | 0.219430000 |
|  | 04 | 4.538200000 | 5.127000000 | 5.534000000 | 5.635200000 | 4.548800000 |
|  | 05 | 5.985000000 | 7.534700000 | 9.401400000 | 9.102300000 | 10.347000000 |
|  | 06 | 5.745400000 | 6.208500000 | 5.649200000 | 6.384300000 | 6.051500000 |
|  | 07 | 6.148900000 | 6.292100000 | 5.822400000 | 5.935500000 | 6.296900000 |
|  | 08 | 3.526600000 | 3.662700000 | 3.672000000 | 3.532900000 | 3.363900000 |
|  | 09 | 6.500300000 | 6.952500000 | 6.598900000 | 3.659000000 | 3.489200000 |
|  | 10 | 4.246300000 | 3.930200000 | 4.131400000 | 3.938400000 | 3.618600000 |
|  | 11 | 0.063423000 | 0.078683000 | 0.022548000 | 0.016636000 | 0.088732000 |
|  | 12 | 3.013770372 | 2.702544826 | 3.209300806 | 2.920086005 | 2.994606806 |
|  | 13 | 3.598004997 | 3.125597031 | 2.917517819 | 2.256025424 | 2.585292821 |
|  | 14 | 2.713994415 | 2.541718716 | 2.843541039 | 2.469266662 | 2.850227790 |
|  | 15 | 3.089922110 | 2.834006907 | 2.700786244 | 3.244378720 | 3.495131898 |
|  | 16 | 3.062715850 | 3.405393490 | 2.364593284 | 2.664082592 | 2.485854949 |
|  | Mean ± SEM | 3.883373172 ± | 4.406841498 ± | 4.410355450 ± | 4.236444088 ± | 4.071667267 ± |
|  |  | 0.544193826 | 0.641205751 | 0.669318106 | 0.668727413 | 0.654798106 |
| Sham + | 01 | 2.437507087 | 1.544082519 | 1.704297126 | 2.221789919 | 2.394244155 |
| MK-801 | 02 | 0.753144240 | 1.246422493 | 0.538942142 | 2.096489455 | 1.942160008 |
|  | 03 | 7.133100000 | 5.055700000 | 6.139800000 | 6.395200000 | 7.375200000 |
|  | 04 | 1.326900000 | 1.837700000 | 1.740300000 | 1.844100000 | 2.170700000 |
|  | 05 | 1.923900000 | 3.265600000 | 3.517200000 | 4.069900000 | 3.876400000 |
|  | 06 | 4.064500000 | 0.001087100 | 2.793987758 | 1.836691688 | 2.879380707 |
|  | 07 | 3.381800000 | 3.909700000 | 3.823000000 | 3.478100000 | 3.733400000 |
|  | 08 | 4.185800000 | 3.867700000 | 3.753100000 | 3.758400000 | 3.337900000 |
|  | 09 | 2.086400000 | 2.553900000 | 2.454000000 | 2.486700000 | 2.334400000 |
|  | 10 | 2.540300000 | 2.080600000 | 1.987100000 | 1.838300000 | 1.862100000 |
|  | 11 | 3.376600000 | 4.651300000 | 1.762900000 | 0.000183530 | 1.262265751 |
|  | 12 | 0.984563053 | 1.609271264 | 0.049320299 | 0.044798863 | 1.545071749 |
|  | 13 | 0.973760656 | 1.331641973 | 1.049979704 | 1.038157094 | 0.621587680 |
|  | 14 | 0.704212756 | 0.820599503 | 0.928677441 | 0.945694642 | 0.501928151 |
|  | 15 | 0.475428760 | 0.823796174 | 0.642321646 | 1.555129896 | 1.058389994 |
|  | 16 | 0.444350010 | 0.541696265 | 1.395194845 | 0.663410798 | 0.749473564 |
|  | Mean ± SEM | 2.299516660 ± | 2.196299831 ± | 2.142507560 ± | 2.142065368 ± | 2.352787610 ± |
|  |  | 0.450784717 | 0.382419134 | 0.391357602 | 0.412167828 | 0.426492077 |
| Concussion + | 01 | 3.046023206 | 4.002414598 | 2.337444092 | 2.973633889 | 1.951983535 |
| Vehicle | 02 | 2.680997602 | 2.060526998 | 3.317770791 | 3.115657613 | 2.832776950 |
|  | 03 | 2.384277565 | 3.927027290 | 2.902022428 | 2.606161276 | 1.730018798 |
|  | 04 | 2.765865042 | 3.824755299 | 2.287677449 | 1.817511506 | 2.952599339 |
|  | 05 | 2.159360093 | 2.537300840 | 2.340639907 | 3.151442386 | 3.525586527 |
|  | 06 | 3.157980985 | 3.897945939 | 2.968296719 | 1.812781906 | 2.982347406 |
|  | 07 | 2.588603693 | 2.392526852 | 2.730473440 | 2.139062855 | 3.029009962 |
|  | 08 | 2.103773891 | 2.195898368 | 2.644725314 | 3.039426291 | 3.482379855 |
|  | 09 | 3.141149261 | 3.240781158 | 3.195533911 | 2.307186136 | 2.976285100 |
|  | 10 | 2.615479836 | 3.098589485 | 2.850169392 | 3.153137012 | 2.638559515 |
|  | 11 | 2.025229657 | 3.667014138 | 3.141149261 | 2.240162226 | 2.801762330 |
|  | 12 | 2.339924425 | 3.798895694 | 2.778617500 | 2.918952375 | 2.338100202 |
|  | 13 | 3.105241778 | 2.402216301 | 2.184762121 | 2.875958664 | 2.086458469 |
|  | 14 | 2.742931886 | 1.898300403 | 3.234433901 | 3.093916548 | 2.541125049 |
|  | 15 | 3.059274665 | 2.914272715 | 2.252404784 | 2.427222800 | 2.382035617 |
|  | 16 | 3.136503965 | 3.958891200 | 2.308169765 | 2.580446510 | 2.934794618 |
|  | Mean ± SEM | 2.690788597 ± | 3.113584830 ± | 2.717143173 ± | 2.640791249 ± | 2.699113954 ± |
|  |  | 0.099270066 | 0.192628401 | 0.097855658 | 0.117150604 | 0.126537782 |
| Concussion + | 01 | 0.363460299 | 0.466884968 | 0.416276285 | 0.448131818 | 1.579377126 |
| MK-801 | 02 | 5.275400000 | 3.675500000 | 4.942000000 | 5.507500000 | 5.034700000 |
|  | 03 | 2.095200000 | 2.136100000 | 2.122400000 | 1.720700000 | 1.844400000 |
|  | 04 | 3.577000000 | 3.183500000 | 3.525100000 | 3.491500000 | 3.528800000 |
|  | 05 | 2.218000000 | 3.195400000 | 2.275200000 | 2.463900000 | 2.261600000 |
|  | 06 | 4.162200000 | 3.729100000 | 4.119900000 | 4.114800000 | 4.559200000 |
|  | 07 | 3.112900000 | 2.830500000 | 2.575700000 | 2.124800000 | 3.828200000 |
|  | 08 | 4.876900000 | 4.421300000 | 4.569100000 | 4.969500000 | 5.347700000 |
|  | 09 | 7.220600000 | 6.477500000 | 6.094100000 | 6.279400000 | 6.676100000 |
|  | 10 | 1.976100000 | 3.201800000 | 3.262800000 | 2.968500000 | 2.928700000 |
|  | 11 | 7.034000000 | 4.192700000 | 3.938300000 | 4.387800000 | 4.046100000 |
|  | 12 | 3.479500000 | 5.424800000 | 3.399100000 | 4.854700000 | 3.409700000 |
|  | 13 | 0.569962358 | 1.550506831 | 0.820008177 | 0.713059170 | 0.753422340 |
|  | 14 | 0.495164315 | 1.174944665 | 1.239112659 | 1.236490392 | 1.276677334 |
|  | 15 | 0.585632516 | 0.909806714 | 1.639057676 | 1.414395680 | 0.666231972 |
|  | 16 | 0.429431419 | 1.551768620 | 1.696937869 | 0.812994318 | 0.834451791 |
|  | Mean ± SEM | 2.966965682 ± | 3.007631987 ± | 2.914693292 ± | 2.969260711 ± | 3.035960035 ± |
|  |  | 0.572792288 | 0.416356642 | 0.397300864 | 0.468135905 | 0.455798167 |
| Concussion + | 01 | 4.733800000 | 4.713700000 | 5.086200000 | 4.164100000 | 4.640800000 |
| Ketamine | 02 | 4.835800000 | 4.607600000 | 4.307300000 | 5.658800000 | 6.547900000 |
|  | 03 | 3.505800000 | 3.787300000 | 3.913900000 | 3.289300000 | 3.368600000 |
|  | 04 | 2.523797567 | 3.309722159 | 2.470624330 | 2.828787601 | 2.865213706 |
|  | 05 | 2.495852480 | 3.036001003 | 2.360547737 | 2.302380389 | 2.413571646 |
|  | 06 | 4.872573639 | 5.416539558 | 4.037535158 | 4.789547902 | 4.451991148 |
|  | 07 | 5.225872666 | 5.964375428 | 6.134952640 | 5.974985047 | 5.878822134 |
|  | 08 | 3.022274574 | 2.482128351 | 2.993712429 | 2.421510967 | 2.659805037 |
|  | 09 | 2.601575955 | 3.742664500 | 2.464029245 | 2.587148660 | 2.524695370 |
|  | 10 | 2.590934200 | 3.595727051 | 2.375548335 | 2.359041430 | 2.823151909 |
|  | 11 | 4.266092257 | 6.147583078 | 5.857925122 | 5.778366330 | 5.151268499 |
|  | 12 | 4.166503699 | 4.470250753 | 3.289269480 | 3.745972992 | 4.195584817 |
|  | 13 | 7.161577346 | 9.272359134 | 7.941503167 | 5.931854573 | 7.817527466 |
|  | 14 | 2.522115134 | 8.551613920 | 7.892783363 | 7.676690748 | 7.212084686 |
|  | 15 | 2.113966450 | 4.471447361 | 4.944394324 | 4.517373055 | 1.384634631 |
|  | 16 | 2.620383922 | 4.841753122 | 2.627272992 | 4.157951750 | 1.306167581 |
|  | Mean ± SEM | 3.703682493 ± | 4.900672839 ± | 4.293593645 ± | 4.261488215 ± | 4.077613664 ± |
|  |  | 0.350106439 | 0.465600628 | 0.469582722 | 0.402686834 | 0.501882414 |

| **Condition** | **Case** | **Time points** |  |
| --- | --- | --- | --- |
|  |  | **50** | **60** |
| Sham + | 01 | 4.580600000 | 3.834300000 |
| Vehicle | 02 | 8.605800000 | 7.203400000 |
|  | 03 | 0.204050000 | 0.183730000 |
|  | 04 | 5.078800000 | 5.261800000 |
|  | 05 | 9.442900000 | 9.168800000 |
|  | 06 | 6.368000000 | 7.904700000 |
|  | 07 | 6.223600000 | 6.183600000 |
|  | 08 | 3.559200000 | 3.297100000 |
|  | 09 | 3.536900000 | 4.094600000 |
|  | 10 | 3.789700000 | 3.766400000 |
|  | 11 | 0.063201000 | 0.086822000 |
|  | 12 | 2.658265683 | 3.019377116 |
|  | 13 | 2.829542950 | 3.190774487 |
|  | 14 | 3.263024469 | 3.296770520 |
|  | 15 | 2.425508854 | 2.086211331 |
|  | 16 | 2.816481740 | 2.284829892 |
|  | Mean ± SEM | 4.090348419 ± | 4.053950959 ± |
|  |  | 0.647798779 | 0.638082872 |
| Sham + | 01 | 1.059851255 | 2.334809645 |
| MK-801 | 02 | 1.213704562 | 0.099144587 |
|  | 03 | 7.157300000 | 6.359100000 |
|  | 04 | 1.961600000 | 1.457200000 |
|  | 05 | 1.586500000 | 2.817900000 |
|  | 06 | 0.001376800 | 1.659858064 |
|  | 07 | 3.798500000 | 4.102800000 |
|  | 08 | 3.599400000 | 4.132200000 |
|  | 09 | 2.198700000 | 2.097900000 |
|  | 10 | 2.183100000 | 2.066100000 |
|  | 11 | 1.606066696 | 2.226521924 |
|  | 12 | 1.486549306 | 0.309201550 |
|  | 13 | 0.483902476 | 1.086462350 |
|  | 14 | 0.968198194 | 0.513541455 |
|  | 15 | 0.869431195 | 1.109562107 |
|  | 16 | 0.456851279 | 0.552060077 |
|  | Mean ± SEM | 1.914439485 ± | 2.057772610 ± |
|  |  | 0.435205271 | 0.416226186 |
| Concussion + | 01 | 2.486419913 | 3.202923446 |
| Vehicle | 02 | 2.808079990 | 2.792960394 |
|  | 03 | 2.481914825 | 1.818224541 |
|  | 04 | 2.286591690 | 1.472661567 |
|  | 05 | 2.601518642 | 2.149965928 |
|  | 06 | 1.952039459 | 3.188256482 |
|  | 07 | 3.238699419 | 3.258980433 |
|  | 08 | 2.462033942 | 2.610831684 |
|  | 09 | 2.581210502 | 2.545540426 |
|  | 10 | 1.794986644 | 1.544269985 |
|  | 11 | 2.424864812 | 2.004870024 |
|  | 12 | 2.216137859 | 2.247980324 |
|  | 13 | 1.922673209 | 1.839886910 |
|  | 14 | 2.484921357 | 2.654214871 |
|  | 15 | 1.880362017 | 2.577927986 |
|  | 16 | 2.264282465 | 2.423778110 |
|  | Mean ± SEM | 2.367921047 ± | 2.395829570 ± |
|  |  | 0.093149631 | 0.141519681 |
| Concussion + | 01 | 0.616245065 | 2.643956226 |
| MK-801 | 02 | 4.884400000 | 5.045100000 |
|  | 03 | 1.549100000 | 1.821000000 |
|  | 04 | 3.623800000 | 3.590300000 |
|  | 05 | 2.617200000 | 2.936600000 |
|  | 06 | 4.067300000 | 4.233900000 |
|  | 07 | 3.651400000 | 3.692100000 |
|  | 08 | 5.488000000 | 5.330500000 |
|  | 09 | 6.252500000 | 6.684300000 |
|  | 10 | 3.076600000 | 3.268200000 |
|  | 11 | 3.966800000 | 2.552700000 |
|  | 12 | 3.872100000 | 3.391200000 |
|  | 13 | 0.879650646 | 1.678363480 |
|  | 14 | 1.661163666 | 0.795625326 |
|  | 15 | 1.668015395 | 1.396342925 |
|  | 16 | 0.695182642 | 0.509643174 |
|  | Mean ± SEM | 3.035591088 ± | 3.098114446 ± |
|  |  | 0.434271259 | 0.421846788 |
| Concussion + | 01 | 4.471900000 | 5.211700000 |
| Ketamine | 02 | 5.554700000 | 5.139600000 |
|  | 03 | 3.427900000 | 3.268600000 |
|  | 04 | 2.221059785 | 2.497263162 |
|  | 05 | 2.447750927 | 2.042335029 |
|  | 06 | 4.532870113 | 4.766476621 |
|  | 07 | 6.196344276 | 6.074398795 |
|  | 08 | 2.401543461 | 2.168237206 |
|  | 09 | 2.488448416 | 2.436815597 |
|  | 10 | 2.295922015 | 2.515207149 |
|  | 11 | 4.782825267 | 5.501763086 |
|  | 12 | 0.968792329 | 3.733282038 |
|  | 13 | 7.365113617 | 5.012704840 |
|  | 14 | 5.939020865 | 5.759553240 |
|  | 15 | 5.530384108 | 5.552581199 |
|  | 16 | 4.985817809 | 5.248592795 |
|  | Mean ± SEM | 4.100649562 ± | 4.183069422 ± |
|  |  | 0.453823807 | 0.366105287 |
