# Supplementary material for: Intranasal delivery of the NMDA receptor antagonist MK-801 attenuates ultra-acute excitotoxic neurochemical responses after concussion in rats: comparative pharmacological evaluation against ketamine
Source: Front Pharmacol. 2026 Mar 16;17:1764201. doi: 10.3389/fphar.2026.1764201 (PMC13033605; doi:10.3389/fphar.2026.1764201)
Supplement: Supplementary file 5 [file Table2.docx]

*SUPPLEMENTARY TABLE 2:* Righting times of rats.

| **Condition** | **Case** | **Righting Time (s)** | **Condition** | **Case** | **Righting Time (s)** |
| --- | --- | --- | --- | --- | --- |
| Sham + Vehicle | 01 | 195 | Sham + MK-801 | 17 | 210 |
|  | 02 | 155 |  | 18 | 50 |
|  | 03 | 120 |  | 19 | 275 |
|  | 04 | 210 |  | 20 | 145 |
|  | 05 | 120 |  | 21 | 90 |
|  | 06 | 65 |  | 22 | 170 |
|  | 07 | 165 |  | 23 | 340 |
|  | 08 | 130 |  | 24 | 70 |
|  | 09 | 165 |  | 25 | 245 |
|  | 10 | 65 |  | 26 | 140 |
|  | 11 | 145 |  | 27 | 170 |
|  | 12 | 190 |  | 28 | 295 |
|  | 13 | 110 |  | 29 | 160 |
|  | 14 | 160 |  | 30 | 250 |
|  | 15 | 195 |  | 31 | 190 |
|  | 16 | 155 |  | 32 | 335 |
|  | Mean ± SEM | 147 ± 11 |  | Mean ± SEM | 196 ± 22 |
| Concussion + Vehicle | 33 | 260 | Concussion + MK-801 | 49 | 220 |
|  | 34 | 600 |  | 50 | 220 |
|  | 35 | 550 |  | 51 | 190 |
|  | 36 | 230 |  | 52 | 290 |
|  | 37 | 260 |  | 53 | 185 |
|  | 38 | 270 |  | 54 | 125 |
|  | 39 | 230 |  | 55 | 90 |
|  | 40 | 610 |  | 56 | 250 |
|  | 41 | 385 |  | 57 | 100 |
|  | 42 | 430 |  | 58 | 130 |
|  | 43 | 530 |  | 59 | 120 |
|  | 44 | 640 |  | 60 | 175 |
|  | 45 | 285 |  | 61 | 230 |
|  | 46 | 240 |  | 62 | 235 |
|  | 47 | 505 |  | 63 | 275 |
|  | 48 | 260 |  | 64 | 200 |
|  | Mean ± SEM | 393 ± 39 |  | Mean ± SEM | 190 ± 15 |

| **Condition** | **Case** | **Righting Time (s)** |
| --- | --- | --- |
| Concussion + Ketamine | 65 | 570 |
|  | 66 | 230 |
|  | 67 | 285 |
|  | 68 | 340 |
|  | 69 | 395 |
|  | 70 | 205 |
|  | 71 | 185 |
|  | 72 | 185 |
|  | 73 | 605 |
|  | 74 | 605 |
|  | 75 | 255 |
|  | 76 | 400 |
|  | 77 | 350 |
|  | 78 | 385 |
|  | 79 | 525 |
|  | 80 | 315 |
|  | Mean ± SEM | 365 ± 36 |
