# Supplementary material for: Intranasal delivery of the NMDA receptor antagonist MK-801 attenuates ultra-acute excitotoxic neurochemical responses after concussion in rats: comparative pharmacological evaluation against ketamine
Source: Front Pharmacol. 2026 Mar 16;17:1764201. doi: 10.3389/fphar.2026.1764201 (PMC13033605; doi:10.3389/fphar.2026.1764201)
Supplement: Supplementary file 6 [file Table5.docx]

*SUPPLEMENTARY TABLE 5:* Extracellular concentrations of glutamate (µg/ml).

| **Condition** | **Case** | **Time Points** |  |  |  |  |
| --- | --- | --- | --- | --- | --- | --- |
|  |  | **-50** | **-40** | **-30** | **-20** | **-10** |
| Sham + | 01 | 0.062104000 | 0.053209000 | 0.044755000 | 0.033597000 | 0.023841000 |
| Vehicle | 02 | 0.060258000 | 0.045651000 | 0.038419000 | 0.032736000 | 0.025701000 |
|  | 03 | 0.009274400 | 0.025933000 | 0.006462500 | 0.009995700 | 0.010489000 |
|  | 04 | 0.061118000 | 0.008687500 | 0.016856000 | 0.013414000 | 0.010793000 |
|  | 05 | 0.187200000 | 0.236940000 | 0.220320000 | 0.151460000 | 0.177400000 |
|  | 06 | 0.051226000 | 0.057527000 | 0.060180000 | 0.057455000 | 4.000900000 |
|  | 07 | 0.100760000 | 0.108360000 | 0.186610000 | 0.089101000 | 0.088567000 |
|  | 08 | 0.054543000 | 0.196050000 | 0.056015000 | 0.056501000 | 0.040126000 |
|  | 09 | 0.089197000 | 0.088851000 | 0.064014000 | 0.036035000 | 0.072291000 |
|  | 10 | 0.100060000 | 0.129100000 | 0.128210000 | 0.156510000 | 0.119400000 |
|  | 11 | 0.070940000 | 0.035438000 | 0.029166000 | 0.026434000 | 0.019561000 |
|  | 12 | 0.019658382 | 0.060310863 | 0.041319931 | 0.040231735 | 0.036173509 |
|  | 13 | 0.095486968 | 0.089780521 | 0.087119342 | 0.087860082 | 0.061989026 |
|  | 14 | 0.085994513 | 0.090713306 | 0.070617284 | 0.125144033 | 0.116337449 |
|  | 15 | 0.093388203 | 0.072139918 | 0.151454047 | 0.129410151 | 0.067229081 |
|  | 16 | 0.073141289 | 0.128326475 | 0.063648834 | 0.140356653 | 0.140891632 |
|  | Mean ± SEM | 0.075883125 ± | 0.090080756 ± | 0.072741210 ± | 0.074263240 ± | 0.065873050 ± |
|  |  | 0.011163993 | 0.017233311 | 0.015560093 | 0.014702358 | 0.014201730 |
| Sham + | 01 | 0.018550836 | 0.026503061 | 0.027928600 | 0.030899607 | 0.016008516 |
| MK-801 | 02 | 0.104761373 | 0.148317953 | 0.085548474 | 0.168571592 | 0.162209291 |
|  | 03 | 0.031248000 | 0.027723000 | 0.028275000 | 0.028775000 | 0.031435000 |
|  | 04 | 0.281030000 | 0.212540000 | 0.217860000 | 0.164610000 | 0.151510000 |
|  | 05 | 0.033304000 | 0.032909000 | 0.023477000 | 0.026892000 | 0.032205000 |
|  | 06 | 0.602140000 | 0.479370000 | 0.399410000 | 0.287300000 | 0.284570000 |
|  | 07 | 0.018654000 | 0.038430000 | 0.020850000 | 0.017775000 | 0.019691000 |
|  | 08 | 0.123840000 | 0.101990000 | 0.117680000 | 0.109670000 | 0.086728000 |
|  | 09 | 0.105750000 | 1.444700000 | 1.430700000 | 1.313300000 | 3.754700000 |
|  | 10 | 0.049192000 | 0.133980000 | 0.106570000 | 0.067610000 | 0.074325000 |
|  | 11 | 0.075535000 | 0.111970000 | 0.094596000 | 0.113210000 | 0.061084000 |
|  | 12 | 0.117619894 | 0.134734315 | 0.106950398 | 0.138821341 | 0.139371518 |
|  | 13 | 0.097982109 | 0.090493031 | 0.097149990 | 0.101682071 | 0.128696247 |
|  | 14 | 0.100552765 | 0.095411454 | 0.122336474 | 0.096317870 | 0.138027876 |
|  | 15 | 0.146467949 | 0.105188861 | 0.087402300 | 0.166349669 | 0.132292193 |
|  | 16 | 0.066792475 | 0.119186306 | 0.128562513 | 0.101860382 | 0.095470891 |
|  | Mean ± SEM | 0.123338775 ± | 0.206465436 ± | 0.193456047 ± | 0.183352783 ± | 0.331770283 ± |
|  |  | 0.035707639 | 0.086787912 | 0.085613916 | 0.077300594 | 0.228837427 |
| Concussion + | 01 | 0.084891595 | 0.057603901 | 0.070882773 | 0.075045039 | 0.064934460 |
| Vehicle | 02 | 0.054140523 | 0.055957632 | 0.068537616 | 0.081319501 | 0.076458346 |
|  | 03 | 0.062604833 | 0.088541343 | 0.071830155 | 0.072296080 | 0.059964590 |
|  | 04 | 0.080838044 | 0.079316022 | 0.060259676 | 0.062604833 | 0.061750637 |
|  | 05 | 0.074819767 | 0.085834001 | 0.054424179 | 0.057150779 | 0.066763202 |
|  | 06 | 0.079887855 | 0.055671945 | 0.060431943 | 0.068565531 | 0.074619508 |
|  | 07 | 0.078624684 | 0.082306365 | 0.063559061 | 0.076159961 | 0.051820815 |
|  | 08 | 0.076514265 | 0.058167478 | 0.076314006 | 0.065654076 | 0.072170189 |
|  | 09 | 0.072629825 | 0.071026657 | 0.058154156 | 0.059867346 | 0.062555011 |
|  | 10 | 0.072582673 | 0.076763485 | 0.057619766 | 0.055843707 | 0.053627562 |
|  | 11 | 0.068181818 | 0.075238904 | 0.061973469 | 0.075914749 | 0.064896894 |
|  | 12 | 0.085643782 | 0.056330944 | 0.056315227 | 0.079514020 | 0.064001006 |
|  | 13 | 0.085007458 | 0.086038910 | 0.071598591 | 0.055047764 | 0.061347551 |
|  | 14 | 0.078945698 | 0.072042908 | 0.069630899 | 0.075248342 | 0.058459488 |
|  | 15 | 0.064806881 | 0.060490653 | 0.058173855 | 0.057793012 | 0.063172427 |
|  | 16 | 0.059125964 | 0.083706243 | 0.066219175 | 0.074788156 | 0.059078359 |
|  | Mean ± SEM | 0.073702854 ± | 0.071564837 ± | 0.064120284 ± | 0.068300806 ± | 0.063476253 ± |
|  |  | 0.002394403 | 0.003089298 | 0.001665392 | 0.002275745 | 0.001685295 |
| Concussion + | 01 | 0.028399394 | 0.034627263 | 0.025414606 | 0.030061845 | 0.028476615 |
| MK-801 | 02 | 0.014403000 | 0.015129000 | 0.014988000 | 0.012471000 | 0.023023000 |
|  | 03 | 2.427100000 | 2.435900000 | 2.550800000 | 2.222400000 | 3.131900000 |
|  | 04 | 0.128810000 | 0.106620000 | 0.149590000 | 0.131120000 | 0.140480000 |
|  | 05 | 0.019568000 | 0.019252000 | 0.011546000 | 0.022331000 | 0.020070000 |
|  | 06 | 0.041525000 | 0.027202000 | 0.014885000 | 0.020378000 | 0.016101000 |
|  | 07 | 0.364870000 | 0.150400000 | 0.135850000 | 0.036976000 | 0.024666000 |
|  | 08 | 0.010663000 | 0.007227500 | 0.009634800 | 0.011112000 | 0.009290500 |
|  | 09 | 0.064948000 | 0.050433000 | 0.050545000 | 0.050325000 | 0.034838000 |
|  | 10 | 0.214310000 | 0.060084000 | 0.020572000 | 0.085976000 | 0.083179000 |
|  | 11 | 0.060634000 | 0.101680000 | 0.066973000 | 0.039959000 | 0.066450000 |
|  | 12 | 0.115700000 | 0.126100000 | 0.084198000 | 0.083557000 | 0.108630000 |
|  | 13 | 0.096481876 | 0.127276881 | 0.111635699 | 0.156823028 | 0.122022540 |
|  | 14 | 0.169159306 | 0.098020104 | 0.103213524 | 0.084267438 | 0.117301249 |
|  | 15 | 0.124657326 | 0.129789826 | 0.068169357 | 0.120392933 | 0.158924764 |
|  | 16 | 0.079622297 | 0.121748401 | 0.070941212 | 0.103213524 | 0.123880597 |
|  | Mean ± SEM | 0.247553200 ± | 0.225718123 ± | 0.218059762 ± | 0.200710236 ± | 0.263077079 ± |
|  |  | 0.147058135 | 0.147829827 | 0.155928379 | 0.135254535 | 0.191675488 |
| Concussion + | 01 | 0.062226000 | 0.042290000 | 0.039152000 | 0.032037000 | 0.045887000 |
| Ketamine | 02 | 0.024632000 | 0.017829000 | 0.008232600 | 0.012046000 | 0.008575300 |
|  | 03 | 0.136850000 | 0.130970000 | 0.108580000 | 0.036499000 | 0.055154000 |
|  | 04 | 0.060251713 | 0.075007426 | 0.139961676 | 0.044810494 | 0.139803843 |
|  | 05 | 0.110898946 | 0.146327087 | 0.098242702 | 0.154866763 | 0.174133389 |
|  | 06 | 0.052688068 | 0.048648026 | 0.053437174 | 0.280877619 | 0.246470637 |
|  | 07 | 0.031508835 | 0.031099046 | 0.033871341 | 0.037715615 | 0.036789973 |
|  | 08 | 0.147771864 | 0.121780705 | 0.111091065 | 0.086734924 | 0.121825808 |
|  | 09 | 0.077601371 | 0.066404312 | 0.130778945 | 0.095688061 | 0.127689324 |
|  | 10 | 0.132842452 | 0.072538451 | 0.110572369 | 0.084333138 | 0.175206351 |
|  | 11 | 0.133914360 | 0.121798584 | 0.127965476 | 0.134013081 | 0.149296908 |
|  | 12 | 0.071982830 | 0.064097404 | 0.058191788 | 0.065961773 | 0.068667738 |
|  | 13 | 0.218928092 | 0.216119195 | 0.194048076 | 0.201678182 | 0.190098010 |
|  | 14 | 0.123811056 | 0.107276160 | 0.119009066 | 0.120487786 | 0.117217087 |
|  | 15 | 0.168479327 | 0.135534666 | 0.131061678 | 0.135433082 | 0.136541120 |
|  | 16 | 0.050259447 | 0.056116819 | 0.061920686 | 0.056317007 | 0.078948214 |
|  | Mean ± SEM | 0.100290398 ± | 0.090864805 ± | 0.095382290 ± | 0.098718720 ± | 0.117019044 ± |
|  |  | 0.013657107 | 0.013023706 | 0.012133059 | 0.017758882 | 0.016033327 |

| **Condition** | **Case** | **Time Points** |  |  |  |  |
| --- | --- | --- | --- | --- | --- | --- |
|  |  | **0** | **10** | **20** | **30** | **40** |
| Sham + | 01 | 0.024490000 | 0.015105000 | 0.011217000 | 0.032192000 | 0.043138000 |
| Vehicle | 02 | 0.028616000 | 0.066261000 | 0.040150000 | 0.036376000 | 0.034955000 |
|  | 03 | 0.007565000 | 0.019261000 | 0.008918600 | 0.011441000 | 0.018064000 |
|  | 04 | 0.018175000 | 0.015216000 | 0.016183000 | 0.016104000 | 0.017564000 |
|  | 05 | 0.110420000 | 0.078588000 | 0.112120000 | 0.113480000 | 0.113040000 |
|  | 06 | 3.752300000 | 4.102300000 | 3.877300000 | 4.110600000 | 4.027900000 |
|  | 07 | 0.122820000 | 0.308340000 | 0.309180000 | 0.540240000 | 0.315550000 |
|  | 08 | 0.063275000 | 0.083627000 | 0.084836000 | 0.100150000 | 0.096715000 |
|  | 09 | 0.058075000 | 0.067507000 | 0.071419000 | 0.129410000 | 0.163190000 |
|  | 10 | 0.130930000 | 0.201000000 | 0.252330000 | 0.145630000 | 0.162000000 |
|  | 11 | 0.021624000 | 0.017933000 | 0.026786000 | 0.012319000 | 0.003830600 |
|  | 12 | 0.021003554 | 0.066152018 | 0.098347741 | 0.074096697 | 0.072979545 |
|  | 13 | 0.072208505 | 0.053936900 | 0.054224966 | 0.087832647 | 0.076419753 |
|  | 14 | 0.092688615 | 0.093717421 | 0.065541838 | 0.077777778 | 0.089396433 |
|  | 15 | 0.139176955 | 0.084718793 | 0.037544582 | 0.055459534 | 0.109917695 |
|  | 16 | 0.073058985 | 0.054910837 | 0.042057613 | 0.073223594 | 0.113552812 |
|  | Mean ± SEM | 0.061521901 ± | 0.065566712 ± | 0.065834024 ± | 0.068963732 ± | 0.079625917 ± |
|  |  | 0.011708568 | 0.012784203 | 0.016697871 | 0.011690160 | 0.013689827 |
| Sham + | 01 | 0.014321087 | 0.015898250 | 0.032894730 | 0.010670341 | 0.016540803 |
| MK-801 | 02 | 0.124806015 | 0.218361401 | 0.025008827 | 0.104631796 | 0.065576038 |
|  | 03 | 0.058665000 | 0.058911000 | 0.073620000 | 0.055920000 | 0.050497000 |
|  | 04 | 0.144910000 | 0.108950000 | 0.113240000 | 0.096145000 | 0.097443000 |
|  | 05 | 0.009246000 | 0.013642000 | 0.013856000 | 0.020505000 | 0.017314000 |
|  | 06 | 0.244710000 | 0.002127100 | 0.001749500 | 0.000857420 | 0.002139000 |
|  | 07 | 0.008381600 | 0.008213600 | 0.006520700 | 0.005611000 | 0.009186600 |
|  | 08 | 0.084185000 | 0.090378000 | 0.077497000 | 0.082616000 | 0.076728000 |
|  | 09 | 3.616300000 | 5.281500000 | 5.328100000 | 5.441500000 | 6.763000000 |
|  | 10 | 0.050752000 | 0.058200000 | 0.057644000 | 0.055886000 | 0.060491000 |
|  | 11 | 0.036600000 | 0.050234000 | 0.015503000 | 0.092664154 | 0.000097203 |
|  | 12 | 0.182010203 | 0.125558156 | 0.046391675 | 0.114888660 | 0.077476652 |
|  | 13 | 0.139484086 | 0.085901513 | 0.061695741 | 0.074831347 | 0.063077655 |
|  | 14 | 0.150925733 | 0.100508187 | 0.094133555 | 0.074073524 | 0.068649886 |
|  | 15 | 0.136928289 | 0.099586912 | 0.061353978 | 0.097818657 | 0.057089364 |
|  | 16 | 0.084742489 | 0.071800053 | 0.100790514 | 0.093687777 | 0.057654016 |
|  | Mean ± SEM | 0.317935469 ± | 0.399360636 ± | 0.381874951 ± | 0.401394167 ± | 0.467685014 ± |
|  |  | 0.220537736 | 0.325757772 | 0.329860412 | 0.336136742 | 0.419753559 |
| Concussion + | 01 | 0.062930981 | 0.316891346 | 0.082111574 | 0.074703361 | 0.093635460 |
| Vehicle | 02 | 0.074221905 | 0.338292850 | 0.064282164 | 0.074843138 | 0.096648444 |
|  | 03 | 0.055584892 | 0.309902466 | 0.076877679 | 0.077374666 | 0.091150525 |
|  | 04 | 0.065555694 | 0.318009567 | 0.076023483 | 0.079626639 | 0.077157234 |
|  | 05 | 0.074958408 | 0.246611005 | 0.069859511 | 0.071569413 | 0.080935363 |
|  | 06 | 0.065191940 | 0.233809847 | 0.053684762 | 0.060231684 | 0.068596340 |
|  | 07 | 0.066886438 | 0.362915152 | 0.061972395 | 0.089577300 | 0.066501325 |
|  | 08 | 0.055333046 | 0.290883603 | 0.065530840 | 0.070768378 | 0.078177953 |
|  | 09 | 0.072991324 | 0.214290205 | 0.086413932 | 0.079183956 | 0.056661008 |
|  | 10 | 0.069454923 | 0.193166101 | 0.073682887 | 0.062382120 | 0.072299761 |
|  | 11 | 0.074453037 | 0.219414058 | 0.062444989 | 0.063278008 | 0.090296115 |
|  | 12 | 0.060731799 | 0.314708286 | 0.070209355 | 0.067003018 | 0.094524079 |
|  | 13 | 0.072899806 | 0.285489860 | 0.078755276 | 0.064949697 | 0.066123965 |
|  | 14 | 0.061807737 | 0.368101177 | 0.069392872 | 0.066647624 | 0.058443619 |
|  | 15 | 0.075756133 | 0.192008632 | 0.054730394 | 0.079215462 | 0.053968707 |
|  | 16 | 0.065425751 | 0.275508585 | 0.075581580 | 0.061791869 | 0.075994160 |
|  | Mean ± SEM | 0.067136488 ± | 0.280000171 ± | 0.070097106 ± | 0.071446646 ± | 0.076319629 ± |
|  |  | 0.001684757 | 0.014379887 | 0.002322787 | 0.002068092 | 0.003516094 |
| Concussion + | 01 | 0.024429870 | 0.020557806 | 0.013247008 | 0.014490899 | 0.063276988 |
| MK-801 | 02 | 0.041488000 | 0.023366000 | 0.024212000 | 0.026214000 | 0.044511000 |
|  | 03 | 1.471300000 | 4.805100000 | 4.504400000 | 3.878000000 | 4.166700000 |
|  | 04 | 0.153690000 | 0.224190000 | 0.139680000 | 0.158380000 | 0.128710000 |
|  | 05 | 0.016673000 | 0.085014000 | 0.030507000 | 0.047107000 | 0.026839000 |
|  | 06 | 0.015826000 | 0.020701000 | 0.019942000 | 0.021659000 | 0.020515000 |
|  | 07 | 0.015242000 | 0.025957000 | 0.024240000 | 0.020619000 | 0.008007400 |
|  | 08 | 0.007295400 | 0.026559000 | 0.008647200 | 0.009666500 | 0.006341700 |
|  | 09 | 0.032015000 | 0.019991000 | 0.019228000 | 0.015305000 | 0.014788000 |
|  | 10 | 0.042430000 | 0.052187000 | 0.050909000 | 0.049449000 | 0.051158000 |
|  | 11 | 0.051984000 | 0.335560000 | 0.207780000 | 0.196630000 | 0.149400000 |
|  | 12 | 0.096772000 | 0.209920000 | 0.119520000 | 0.183970000 | 0.171430000 |
|  | 13 | 0.112321048 | 0.087861712 | 0.099010052 | 0.065367042 | 0.048263783 |
|  | 14 | 0.118534877 | 0.076713372 | 0.055254341 | 0.040694487 | 0.058346025 |
|  | 15 | 0.091821505 | 0.080155346 | 0.058300335 | 0.090862016 | 0.055878769 |
|  | 16 | 0.133749619 | 0.087541882 | 0.094760889 | 0.070773683 | 0.050776729 |
|  | Mean ± SEM | 0.151598270 ± | 0.386335945 ± | 0.341852364 ± | 0.305574227 ± | 0.316558900 ± |
|  |  | 0.088779758 | 0.295442088 | 0.277846835 | 0.238645866 | 0.256967995 |
| Concussion + | 01 | 0.042226000 | 0.054433000 | 0.034513000 | 0.030132000 | 0.032107000 |
| Ketamine | 02 | 0.008437200 | 0.017172000 | 0.010855000 | 0.012398000 | 0.008722300 |
|  | 03 | 0.019209000 | 0.351100000 | 0.079081000 | 0.068029000 | 0.063635000 |
|  | 04 | 0.130726636 | 0.118305929 | 0.094216390 | 0.031659516 | 0.114602989 |
|  | 05 | 0.071327503 | 0.157073284 | 0.083246007 | 0.057162626 | 0.106206023 |
|  | 06 | 0.277858581 | 0.503236671 | 0.166965334 | 0.200804817 | 0.319554891 |
|  | 07 | 0.041089204 | 0.091083628 | 0.054770005 | 0.044540738 | 0.052998547 |
|  | 08 | 0.060901628 | 0.327342023 | 0.059300438 | 0.034008389 | 0.046378152 |
|  | 09 | 0.088212079 | 0.373708899 | 0.087885075 | 0.079709982 | 0.120089306 |
|  | 10 | 0.128546299 | 0.696044382 | 0.159216995 | 0.034346669 | 0.099307654 |
|  | 11 | 0.097118908 | 0.276408131 | 0.164068631 | 0.124286082 | 0.113098166 |
|  | 12 | 0.076170885 | 0.099274181 | 0.043226556 | 0.029365513 | 0.031244443 |
|  | 13 | 0.194413004 | 0.281141855 | 0.098953781 | 0.081734576 | 0.113948393 |
|  | 14 | 0.041000453 | 0.621574419 | 0.198361228 | 0.192636261 | 0.156230947 |
|  | 15 | 0.058619266 | 0.280098248 | 0.116782429 | 0.101520193 | 0.171436362 |
|  | 16 | 0.113650623 | 0.751235845 | 0.517954871 | 0.109600104 | 0.115475204 |
|  | Mean ± SEM | 0.090594204 ± | 0.312452031 ± | 0.123087296 ± | 0.076995904 ± | 0.104064711 ± |
|  |  | 0.017206561 | 0.057390183 | 0.029458354 | 0.014218406 | 0.018432507 |

| **Condition** | **Case** | **Time Points** |  |
| --- | --- | --- | --- |
|  |  | **50** | **60** |
| Sham + | 01 | 0.065705000 | 0.121870000 |
| Vehicle | 02 | 0.102550000 | 0.119290000 |
|  | 03 | 0.010207000 | 0.008216700 |
|  | 04 | 0.018436000 | 0.016983000 |
|  | 05 | 0.127080000 | 0.107750000 |
|  | 06 | 3.041700000 | 0.862610000 |
|  | 07 | 0.290320000 | 0.304050000 |
|  | 08 | 0.074608000 | 0.074330000 |
|  | 09 | 0.136040000 | 0.020438000 |
|  | 10 | 0.165010000 | 0.167900000 |
|  | 11 | 0.006902600 | 0.010229000 |
|  | 12 | 0.048900441 | 0.067120985 |
|  | 13 | 0.062085048 | 0.068422497 |
|  | 14 | 0.117133059 | 0.088847737 |
|  | 15 | 0.077297668 | 0.118902606 |
|  | 16 | 0.148573388 | 0.087928669 |
|  | Mean ± SEM | 0.082894872 ± | 0.077016371 ± |
|  |  | 0.013785908 | 0.013091997 |
| Sham + | 01 | 0.042740994 | 0.024094282 |
| MK-801 | 02 | 0.018348389 | 0.061718023 |
|  | 03 | 0.038731000 | 0.030172000 |
|  | 04 | 0.086697000 | 0.051391000 |
|  | 05 | 0.018035000 | 0.019854000 |
|  | 06 | 0.004248300 | 0.068634673 |
|  | 07 | 0.007529800 | 0.007370300 |
|  | 08 | 0.080034000 | 0.089596000 |
|  | 09 | 6.631600000 | 5.710200000 |
|  | 10 | 0.054659000 | 0.049909000 |
|  | 11 | 0.002455500 | 0.000616680 |
|  | 12 | 0.106636011 | 0.070481550 |
|  | 13 | 0.089928378 | 0.082870219 |
|  | 14 | 0.082602752 | 0.046554132 |
|  | 15 | 0.068887634 | 0.065945496 |
|  | 16 | 0.088204701 | 0.054593004 |
|  | Mean ± SEM | 0.463833654 ± | 0.402125022 ± |
|  |  | 0.411276662 | 0.353930679 |
| Concussion + | 01 | 0.079316022 | 0.057743679 |
| Vehicle | 02 | 0.067046655 | 0.052975710 |
|  | 03 | 0.083571473 | 0.077933776 |
|  | 04 | 0.078213332 | 0.066611791 |
|  | 05 | 0.081135621 | 0.067425596 |
|  | 06 | 0.062419126 | 0.082445006 |
|  | 07 | 0.064514141 | 0.050234149 |
|  | 08 | 0.061202169 | 0.079456528 |
|  | 09 | 0.056158054 | 0.052590218 |
|  | 10 | 0.059427260 | 0.061391928 |
|  | 11 | 0.063843833 | 0.062429272 |
|  | 12 | 0.068181818 | 0.085376587 |
|  | 13 | 0.072979149 | 0.086038910 |
|  | 14 | 0.070424323 | 0.077231902 |
|  | 15 | 0.061966422 | 0.070424323 |
|  | 16 | 0.062966137 | 0.056110952 |
|  | Mean ± SEM | 0.068335346 ± | 0.067901270 ± |
|  |  | 0.002101032 | 0.003068117 |
| Concussion + | 01 | 0.021556345 | 0.061660321 |
| MK-801 | 02 | 0.030421000 | 0.026884000 |
|  | 03 | 2.944400000 | 3.533300000 |
|  | 04 | 0.156500000 | 0.151620000 |
|  | 05 | 0.031144000 | 0.028967000 |
|  | 06 | 0.019700000 | 0.018855000 |
|  | 07 | 0.021743000 | 0.008172900 |
|  | 08 | 0.009768900 | 0.007606000 |
|  | 09 | 0.015014000 | 0.015690000 |
|  | 10 | 0.042696000 | 0.040460000 |
|  | 11 | 0.177380000 | 0.140530000 |
|  | 12 | 0.178820000 | 0.157670000 |
|  | 13 | 0.052771855 | 0.062260128 |
|  | 14 | 0.066524520 | 0.067712458 |
|  | 15 | 0.041197076 | 0.060188852 |
|  | 16 | 0.051401157 | 0.051903747 |
|  | Mean ± SEM | 0.241314866 ± | 0.277092525 ± |
|  |  | 0.180769333 | 0.217426491 |
| Concussion + | 01 | 0.032551000 | 0.029886000 |
| Ketamine | 02 | 0.008748100 | 0.006461400 |
|  | 03 | 0.080008000 | 0.066409000 |
|  | 04 | 0.097672122 | 0.060967374 |
|  | 05 | 0.107316129 | 0.110975551 |
|  | 06 | 0.383956430 | 0.367069088 |
|  | 07 | 0.043637879 | 0.041052476 |
|  | 08 | 0.061927743 | 0.049930089 |
|  | 09 | 0.063416174 | 0.092587163 |
|  | 10 | 0.073542014 | 0.054722385 |
|  | 11 | 0.124058094 | 0.113530323 |
|  | 12 | 0.156132630 | 0.041656425 |
|  | 13 | 0.071776600 | 0.039135657 |
|  | 14 | 0.141127126 | 0.126455397 |
|  | 15 | 0.099372049 | 0.124513220 |
|  | 16 | 0.051658097 | 0.050917512 |
|  | Mean ± SEM | 0.099806262 ± | 0.086016816 ± |
|  |  | 0.021329278 | 0.020788963 |
