# Supplementary material for: Intranasal delivery of the NMDA receptor antagonist MK-801 attenuates ultra-acute excitotoxic neurochemical responses after concussion in rats: comparative pharmacological evaluation against ketamine
Source: Front Pharmacol. 2026 Mar 16;17:1764201. doi: 10.3389/fphar.2026.1764201 (PMC13033605; doi:10.3389/fphar.2026.1764201)
Supplement: Supplementary file 7 [file Table3.docx]

*SUPPLEMENTARY TABLE 3:* Accuracy of the standard calibration curve.

| **Sample** | **STD Levels** | **Nominal**  **Con. (ng/ml)** | **Experimental**  **Conc. (ng/ml)** | **Experimental**  **Conc. (ng/g)*** | **Accuracy (%)** | **Bias (%)** |
| --- | --- | --- | --- | --- | --- | --- |
| Brain | 1 | 0.00 | - | - | - | - |
|  | 2 | 7.50 | 6.86 | 41.20 | 91.50 | 8.50 |
|  | 3 | 15.00 | 18.20 | 109.00 | 121.10 | -21.10 |
|  | 4 | 37.50 | 32.00 | 192.00 | 85.40 | 14.60 |
|  | 5 | 75.00 | 73.90 | 443.00 | 98.50 | 1.50 |
|  | 6 | 150.00 | 156.00 | 938.00 | 104.20 | -4.20 |
|  | 7 | 375.00 | 373.00 | 2238.00 | 99.40 | 0.60 |
| Plasma | 1 | 0.00 | - |  | - | - |
|  | 2 | 1.50 | 1.48 |  | 98.40 | 1.60 |
|  | 3 | 3.75 | 3.31 |  | 88.20 | 11.80 |
|  | 4 | 7.50 | 7.14 |  | 95.20 | 4.80 |
|  | 5 | 15.00 | 14.20 |  | 94.90 | 5.10 |
|  | 6 | 37.50 | 31.90 |  | 85.10 | 14.90 |
|  | 7 | 75.00 | 72.20 |  | 96.30 | 3.70 |
|  | 8 | 150.00 | 148.00 |  | 98.80 | 1.20 |

*Actual concentration of MK-801 spiked in brain homogenates. Since the brain were homogenized in five volumes of solvent, resulting in an approximate six-fold dilution factor, the concentrations in ng/ml should be converted to ng/g, using a multiplication factor of six.
