# Supplementary material for: Intranasal delivery of the NMDA receptor antagonist MK-801 attenuates ultra-acute excitotoxic neurochemical responses after concussion in rats: comparative pharmacological evaluation against ketamine
Source: Front Pharmacol. 2026 Mar 16;17:1764201. doi: 10.3389/fphar.2026.1764201 (PMC13033605; doi:10.3389/fphar.2026.1764201)
Supplement: Supplementary file 8 [file Table4.docx]

*SUPPLEMENTARY TABLE 4:* Concentrations of MK-801 in brain homogenates and plasma samples of rats.

| **Condition** | **Case** | **Brain Concentration (ng/g)** | **Plasma Concentration (ng/ml)** | **Brain/Plasma Ratio** |
| --- | --- | --- | --- | --- |
| Sham + MK-801 | 17 | 521.00 | 56.80 | 9.17 |
|  | 18 | 399.49 | 26.69 | 14.97 |
|  | 19 | 80.30 | 8.75 | 9.18 |
|  | 20 | 191.00 | 23.10 | 8.27 |
|  | 21 | 390.00 | 49.90 | 7.82 |
|  | 22 | 80.40 | 5.96 | 13.49 |
|  | 23 | 413.00 | 33.40 | 12.37 |
|  | 24 | 119.95 | 18.80 | 6.38 |
|  | 25 | 245.00 | 22.50 | 10.89 |
|  | 26 | 50.10 | 3.81 | 13.15 |
|  | 27 | 325.48 | 13.61 | 23.91 |
|  | 28 | 136.00 | 12.20 | 11.15 |
|  | Mean ± SEM | 245.98 ± 45.92 | 22.96 ± 4.82 | 11.73 ± 1.33 |
| Concussion + MK-801 | 49 | 49.40 | 5.89 | 8.39 |
|  | 50 | 408.57 | 40.80 | 10.01 |
|  | 51 | 118.00 | 9.42 | 12.53 |
|  | 52 | 940.00 | 29.60 | 31.76 |
|  | 53 | 55.70 | 5.30 | 10.51 |
|  | 54 | 330.00 | 24.70 | 13.36 |
|  | 55 | 689.00 | 53.30 | 12.93 |
|  | 56 | 103.00 | 9.38 | 10.98 |
|  | 57 | 338.00 | 27.70 | 12.20 |
|  | 58 | 209.31 | 23.02 | 9.09 |
|  | 59 | 53.40 | 3.85 | 13.87 |
|  | 60 | 267.00 | 22.10 | 12.08 |
|  | Mean ± SEM | 296.78 ± 79.75 | 21.26 ± 4.45 | 13.14 ± 1.76 |
