# Supplementary material for: Intranasal delivery of the NMDA receptor antagonist MK-801 attenuates ultra-acute excitotoxic neurochemical responses after concussion in rats: comparative pharmacological evaluation against ketamine
Source: Front Pharmacol. 2026 Mar 16;17:1764201. doi: 10.3389/fphar.2026.1764201 (PMC13033605; doi:10.3389/fphar.2026.1764201)
Supplement: Supplementary file 9 [file Table6.docx]

*SUPPLEMENTARY TABLE 6:* Extracellular concentrations of GABA (µg/ml).

| **Condition** | **Case** | **Time points** |  |  |  |  |
| --- | --- | --- | --- | --- | --- | --- |
|  |  | **-50** | **-40** | **-30** | **-20** | **-10** |
| Sham + | 01 | 0.003346000 | 0.001359100 | 0.001205000 | 0.000489000 | 0.000760990 |
| Vehicle | 02 | 0.001247500 | 0.000877170 | 0.000534890 | 0.000581570 | 0.000678510 |
|  | 03 | 0.000261590 | 0.001157800 | 0.000112130 | 0.000600850 | 0.000663830 |
|  | 04 | 0.003186200 | 0.000959410 | 0.000671540 | 0.000985930 | 0.001095200 |
|  | 05 | 0.002042700 | 0.008478800 | 0.002902100 | 0.002468900 | 0.002948600 |
|  | 06 | 0.002009400 | 0.003185000 | 0.003229400 | 0.001898700 | 1.332800000 |
|  | 07 | 0.003625700 | 0.003359600 | 0.004569600 | 0.003225000 | 0.002641500 |
|  | 08 | 0.003448700 | 0.004077800 | 0.003881300 | 0.003306800 | 0.002979500 |
|  | 09 | 0.005859300 | 0.004233100 | 0.003082900 | 0.003351800 | 0.004384500 |
|  | 10 | 0.002042600 | 0.002353400 | 0.002860800 | 0.001593400 | 0.002311000 |
|  | 11 | 0.010482000 | 0.005784000 | 0.005190000 | 0.004997100 | 0.008232200 |
|  | 12 | 0.000109458 | 0.000088269 | 0.000113555 | 0.000092617 | 0.000059224 |
|  | 13 | 0.000042353 | 0.000070588 | 0.000084705 | 0.000056470 | 0.000042353 |
|  | 14 | 0.000042353 | 0.000098823 | 0.000042353 | 0.000042353 | 0.000056470 |
|  | 15 | 0.000014118 | 0.000056470 | 0.000084705 | 0.000070588 | 0.000098823 |
|  | 16 | 0.000056470 | 0.000084705 | 0.000042353 | 0.000070588 | 0.000042353 |
|  | Mean ± SEM | 0.002298667 ± | 0.002119960 ± | 0.001486309 ± | 0.001336283 ± | 0.001739539 ± |
|  |  | 0.000789558 | 0.000693066 | 0.000465373 | 0.000423938 | 0.000621241 |
| Sham + | 01 | 0.000044262 | 0.000088523 | 0.000103277 | 0.000059015 | 0.000118031 |
| MK-801 | 02 | 0.000088523 | 0.000044262 | 0.000132785 | 0.000073769 | 0.000029508 |
|  | 03 | 0.002098200 | 0.001871500 | 0.000584910 | 0.000537980 | 0.000921480 |
|  | 04 | 0.107840000 | 0.068834000 | 0.075079000 | 0.041561000 | 0.030999000 |
|  | 05 | 0.002426700 | 0.001310900 | 0.001110700 | 0.001435300 | 0.001477200 |
|  | 06 | 0.002391200 | 0.003499800 | 0.002151300 | 0.002723900 | 0.002974500 |
|  | 07 | 0.001163100 | 0.001225800 | 0.000005728 | 0.002834510 | 0.000915639 |
|  | 08 | 0.005981500 | 0.004935600 | 0.003879000 | 0.004025100 | 0.003089200 |
|  | 09 | 0.008855500 | 0.532180000 | 0.474430000 | 0.410940000 | 1.846100000 |
|  | 10 | 0.000161490 | 0.002714100 | 0.014658000 | 0.004001900 | 0.005437500 |
|  | 11 | 0.001501496 | 0.000389501 | 0.001187935 | 0.002254853 | 0.002781725 |
|  | 12 | 0.000059015 | 0.000044262 | 0.000103277 | 0.000029508 | 0.000132785 |
|  | 13 | 0.000098342 | 0.000098342 | 0.000056196 | 0.000056196 | 0.000098342 |
|  | 14 | 0.000056196 | 0.000126440 | 0.000126440 | 0.000084293 | 0.000056196 |
|  | 15 | 0.000070244 | 0.000028098 | 0.000070244 | 0.000070244 | 0.000112391 |
|  | 16 | 0.000070244 | 0.000070244 | 0.000098342 | 0.000084293 | 0.000098342 |
|  | Mean ± SEM | 0.008306626 ± | 0.038591336 ± | 0.035861071 ± | 0.029423241 ± | 0.118458865 ± |
|  |  | 0.006664377 | 0.033177296 | 0.029607291 | 0.025560590 | 0.115191611 |
| Concussion + | 01 | 0.000058423 | 0.000087635 | 0.000116846 | 0.000029212 | 0.000043817 |
| Vehicle | 02 | 0.000058423 | 0.000087635 | 0.000043817 | 0.000073029 | 0.000043817 |
|  | 03 | 0.000058423 | 0.000058423 | 0.000116846 | 0.000043817 | 0.000058423 |
|  | 04 | 0.000058423 | 0.000102241 | 0.000058423 | 0.000073029 | 0.000043817 |
|  | 05 | 0.000116171 | 0.000116171 | 0.000043564 | 0.000043564 | 0.000043564 |
|  | 06 | 0.000072607 | 0.000116171 | 0.000058086 | 0.000072607 | 0.000072607 |
|  | 07 | 0.000116171 | 0.000101650 | 0.000058086 | 0.000072607 | 0.000043564 |
|  | 08 | 0.000058086 | 0.000072607 | 0.000043564 | 0.000101650 | 0.000058086 |
|  | 09 | 0.000072677 | 0.000072677 | 0.000101747 | 0.000029071 | 0.000058141 |
|  | 10 | 0.000058141 | 0.000116282 | 0.000043606 | 0.000072677 | 0.000058141 |
|  | 11 | 0.000072677 | 0.000101747 | 0.000101747 | 0.000087212 | 0.000043606 |
|  | 12 | 0.000101747 | 0.000116282 | 0.000058141 | 0.000087212 | 0.000043606 |
|  | 13 | 0.000115105 | 0.000086328 | 0.000071940 | 0.000028776 | 0.000086328 |
|  | 14 | 0.000100717 | 0.000071940 | 0.000043164 | 0.000071940 | 0.000100717 |
|  | 15 | 0.000057552 | 0.000057552 | 0.000086328 | 0.000057552 | 0.000043164 |
|  | 16 | 0.000115105 | 0.000100717 | 0.000071940 | 0.000100717 | 0.000071940 |
|  | Mean ± SEM | 0.000080653 ± | 0.000091629 ± | 0.000069865 ± | 0.000065292 ± | 0.000057084 ± |
|  |  | 0.000006279 | 0.000005103 | 0.000006689 | 0.000006057 | 0.000004414 |
| Concussion + | 01 | 0.000097401 | 0.000125230 | 0.000083486 | 0.000055658 | 0.000069572 |
| MK-801 | 02 | 0.000137960 | 0.000758276 | 0.000289539 | 0.000272666 | 0.002675096 |
|  | 03 | 1.002900000 | 1.035600000 | 1.125600000 | 0.984640000 | 1.469200000 |
|  | 04 | 0.000912970 | 0.001022700 | 0.000770640 | 0.000554090 | 0.000309930 |
|  | 05 | 0.002694400 | 0.003362500 | 0.001217400 | 0.001644500 | 0.002653500 |
|  | 06 | 0.001315000 | 0.000899620 | 0.000765560 | 0.000904590 | 0.001071200 |
|  | 07 | 0.002314300 | 0.000216810 | 0.000608820 | 0.000035565 | 0.001002469 |
|  | 08 | 0.001858287 | 0.000321774 | 0.000556420 | 0.000165457 | 0.002729799 |
|  | 09 | 0.000408255 | 0.000829667 | 0.000394846 | 0.000825009 | 0.002120384 |
|  | 10 | 0.005371100 | 0.001138443 | 0.000182940 | 0.003655700 | 0.002961700 |
|  | 11 | 0.001686078 | 0.000418501 | 0.000120592 | 0.000252225 | 0.011522000 |
|  | 12 | 0.001976500 | 0.002088900 | 0.000282330 | 0.000483103 | 0.000452040 |
|  | 13 | 0.000028502 | 0.000085507 | 0.000057004 | 0.000071256 | 0.000099758 |
|  | 14 | 0.000042753 | 0.000042753 | 0.000128260 | 0.000042753 | 0.000099758 |
|  | 15 | 0.000028502 | 0.000042753 | 0.000057004 | 0.000071256 | 0.000057004 |
|  | 16 | 0.000071256 | 0.000057004 | 0.000042753 | 0.000085507 | 0.000099758 |
|  | Mean ± SEM | 0.063865204 ± | 0.065438152 ± | 0.070697350 ± | 0.062109958 ± | 0.093570248 ± |
|  |  | 0.062603335 | 0.064677835 | 0.070326893 | 0.061502435 | 0.091711335 |
| Concussion + | 01 | 0.001031600 | 0.000693250 | 0.001272300 | 0.000629950 | 0.001226100 |
| Ketamine | 02 | 0.001382812 | 0.003479756 | 0.004139877 | 0.005921831 | 0.002537859 |
|  | 03 | 0.001530146 | 0.005118922 | 0.000913180 | 0.008127490 | 0.006566494 |
|  | 04 | 0.000098164 | 0.000091702 | 0.000085859 | 0.000071613 | 0.000052614 |
|  | 05 | 0.000079385 | 0.000082159 | 0.000052190 | 0.000068594 | 0.000060547 |
|  | 06 | 0.000050324 | 0.000088068 | 0.000062906 | 0.000062906 | 0.000037743 |
|  | 07 | 0.000062906 | 0.000088068 | 0.000062906 | 0.000037743 | 0.000037743 |
|  | 08 | 0.000086842 | 0.000086842 | 0.000037218 | 0.000049624 | 0.000037218 |
|  | 09 | 0.000062030 | 0.000074436 | 0.000049624 | 0.000049624 | 0.000049624 |
|  | 10 | 0.000062030 | 0.000062030 | 0.000037218 | 0.000049624 | 0.000037218 |
|  | 11 | 0.000072681 | 0.000072681 | 0.000036340 | 0.000048454 | 0.000048454 |
|  | 12 | 0.000046899 | 0.000058624 | 0.000070349 | 0.000058624 | 0.000035174 |
|  | 13 | 0.000073980 | 0.000061650 | 0.000049320 | 0.000036990 | 0.000036990 |
|  | 14 | 0.000084794 | 0.000072681 | 0.000060567 | 0.000060567 | 0.000036340 |
|  | 15 | 0.000063858 | 0.000076630 | 0.000038315 | 0.000051087 | 0.000038315 |
|  | 16 | 0.000076630 | 0.000063858 | 0.000038315 | 0.000063858 | 0.000051087 |
|  | Mean ± SEM | 0.000070810 ± | 0.000075341 ± | 0.000052394 ± | 0.000054562 ± | 0.000043005 ± |
|  |  | 0.000004065 | 0.000003165 | 0.000004283 | 0.000003007 | 0.000002300 |

| **Condition** | **Case** | **Time points** |  |  |  |  |
| --- | --- | --- | --- | --- | --- | --- |
|  |  | **0** | **10** | **20** | **30** | **40** |
| Sham + | 01 | 0.000405350 | 0.000528220 | 0.000850640 | 0.000360900 | 0.000476710 |
| Vehicle | 02 | 0.000564270 | 0.000758840 | 0.000799090 | 0.000665650 | 0.000716280 |
|  | 03 | 0.000307310 | 0.000316580 | 0.000541470 | 0.001043600 | 0.000673440 |
|  | 04 | 0.001186300 | 0.000957680 | 0.001645000 | 0.001205400 | 0.001405100 |
|  | 05 | 0.003480500 | 0.002081300 | 0.002467300 | 0.002316800 | 0.002130400 |
|  | 06 | 1.246700000 | 1.423500000 | 1.353000000 | 1.457900000 | 1.427300000 |
|  | 07 | 0.002861300 | 0.009000200 | 0.007330300 | 0.007364000 | 0.007402200 |
|  | 08 | 0.004378200 | 0.003760300 | 0.002893600 | 0.003108900 | 0.002713500 |
|  | 09 | 0.004455100 | 0.004223100 | 0.004229200 | 0.001598400 | 0.002075200 |
|  | 10 | 0.002014500 | 0.006709600 | 0.008425200 | 0.006222700 | 0.005958500 |
|  | 11 | 0.003811300 | 0.001791600 | 0.002075300 | 0.002110100 | 0.001117800 |
|  | 12 | 0.000110668 | 0.000039264 | 0.000018282 | 0.000071611 | 0.000102985 |
|  | 13 | 0.000084705 | 0.000084705 | 0.000084705 | 0.000084705 | 0.000028235 |
|  | 14 | 0.000084705 | 0.000112940 | 0.000028235 | 0.000098823 | 0.000070588 |
|  | 15 | 0.000127058 | 0.000056470 | 0.000028235 | 0.000098823 | 0.000070588 |
|  | 16 | 0.000098823 | 0.000098823 | 0.000042353 | 0.000070588 | 0.000056470 |
|  | Mean ± SEM | 0.001507771 ± | 0.001537102 ± | 0.001723472 ± | 0.001361214 ± | 0.001256843 ± |
|  |  | 0.000468364 | 0.000541487 | 0.000622052 | 0.000456779 | 0.000432466 |
| Sham + | 01 | 0.000132785 | 0.000162292 | 0.000118031 | 0.000132785 | 0.000059015 |
| MK-801 | 02 | 0.000088523 | 0.000073769 | 0.000059015 | 0.000147538 | 0.000029508 |
|  | 03 | 0.000238530 | 0.000672230 | 0.001512900 | 0.001634200 | 0.000923980 |
|  | 04 | 0.027543000 | 0.022237000 | 0.027450000 | 0.020396000 | 0.017733000 |
|  | 05 | 0.000904990 | 0.001206300 | 0.001012500 | 0.001448500 | 0.000626400 |
|  | 06 | 0.002284700 | 0.000291290 | 0.000410950 | 0.000194470 | 0.000172860 |
|  | 07 | 0.021087000 | 0.000436864 | 0.001384537 | 0.000584687 | 0.002268663 |
|  | 08 | 0.003016500 | 0.002676700 | 0.002496900 | 0.002119000 | 0.002773100 |
|  | 09 | 1.789800000 | 2.676500000 | 2.977600000 | 2.962000000 | 3.251000000 |
|  | 10 | 0.003326400 | 0.006665200 | 0.008740700 | 0.007972000 | 0.007972000 |
|  | 11 | 0.002167767 | 0.001603049 | 0.001143075 | 0.001545117 | 0.001764089 |
|  | 12 | 0.000147538 | 0.000147538 | 0.000029508 | 0.000103277 | 0.000073769 |
|  | 13 | 0.000056196 | 0.000098342 | 0.000028098 | 0.000056196 | 0.000098342 |
|  | 14 | 0.000140489 | 0.000042147 | 0.000126440 | 0.000098342 | 0.000056196 |
|  | 15 | 0.000098342 | 0.000070244 | 0.000070244 | 0.000112391 | 0.000042147 |
|  | 16 | 0.000042147 | 0.000112391 | 0.000126440 | 0.000112391 | 0.000056196 |
|  | Mean ± SEM | 0.115692182 ± | 0.169562210 ± | 0.188894334 ± | 0.187416056 ± | 0.205353079 ± |
|  |  | 0.111625555 | 0.167134946 | 0.185921663 | 0.184976792 | 0.203046362 |
| Concussion + | 01 | 0.000102241 | 0.000043817 | 0.000058423 | 0.000087635 | 0.000043817 |
| Vehicle | 02 | 0.000102241 | 0.000058423 | 0.000102241 | 0.000029212 | 0.000058423 |
|  | 03 | 0.000073029 | 0.000029212 | 0.000058423 | 0.000043817 | 0.000087635 |
|  | 04 | 0.000087635 | 0.000087635 | 0.000087635 | 0.000102241 | 0.000087635 |
|  | 05 | 0.000072607 | 0.000043564 | 0.000072607 | 0.000058086 | 0.000101650 |
|  | 06 | 0.000072607 | 0.000043564 | 0.000043564 | 0.000072607 | 0.000087128 |
|  | 07 | 0.000087128 | 0.000072607 | 0.000101650 | 0.000101650 | 0.000058086 |
|  | 08 | 0.000087128 | 0.000043564 | 0.000087128 | 0.000101650 | 0.000101650 |
|  | 09 | 0.000072677 | 0.000087212 | 0.000043606 | 0.000072677 | 0.000043606 |
|  | 10 | 0.000087212 | 0.000087212 | 0.000058141 | 0.000101747 | 0.000043606 |
|  | 11 | 0.000087212 | 0.000043606 | 0.000072677 | 0.000029071 | 0.000072677 |
|  | 12 | 0.000072677 | 0.000072677 | 0.000101747 | 0.000058141 | 0.000087212 |
|  | 13 | 0.000086328 | 0.000100717 | 0.000086328 | 0.000100717 | 0.000057552 |
|  | 14 | 0.000071940 | 0.000100717 | 0.000086328 | 0.000086328 | 0.000043164 |
|  | 15 | 0.000057552 | 0.000100717 | 0.000057552 | 0.000043164 | 0.000057552 |
|  | 16 | 0.000057552 | 0.000028776 | 0.000086328 | 0.000043164 | 0.000071940 |
|  | Mean ± SEM | 0.000079860 ± | 0.000065251 ± | 0.000075274 ± | 0.000070744 ± | 0.000068958 ± |
|  |  | 0.000003311 | 0.000006571 | 0.000004992 | 0.000006869 | 0.000005241 |
| Concussion + | 01 | 0.000111315 | 0.000111315 | 0.000055658 | 0.000125230 | 0.000083486 |
| MK-801 | 02 | 0.000519328 | 0.000516113 | 0.000400918 | 0.001380212 | 0.000166280 |
|  | 03 | 0.622490000 | 2.507800000 | 2.249800000 | 1.835700000 | 1.909800000 |
|  | 04 | 0.000988130 | 0.002558800 | 0.001722100 | 0.001276100 | 0.001019100 |
|  | 05 | 0.002216800 | 0.008706500 | 0.004862900 | 0.005373800 | 0.003106100 |
|  | 06 | 0.001050100 | 0.001100400 | 0.001360700 | 0.001359400 | 0.001187900 |
|  | 07 | 0.000205468 | 0.000342780 | 0.000992675 | 0.000011137 | 0.000447433 |
|  | 08 | 0.000139733 | 0.002083702 | 0.000596133 | 0.001938059 | 0.000772002 |
|  | 09 | 0.000522017 | 0.001527330 | 0.000998490 | 0.003466780 | 0.000709975 |
|  | 10 | 0.000465171 | 0.001388954 | 0.000430971 | 0.002717888 | 0.000230287 |
|  | 11 | 0.000283085 | 0.003613257 | 0.000331200 | 0.003437300 | 0.000099409 |
|  | 12 | 0.000412878 | 0.004863200 | 0.000867009 | 0.009630600 | 0.000295490 |
|  | 13 | 0.000057004 | 0.000057004 | 0.000085507 | 0.000099758 | 0.000071256 |
|  | 14 | 0.000128260 | 0.000156762 | 0.000142511 | 0.000071256 | 0.000057004 |
|  | 15 | 0.000099758 | 0.000085507 | 0.000099758 | 0.000128260 | 0.000028502 |
|  | 16 | 0.000128260 | 0.000142511 | 0.000099758 | 0.000128260 | 0.000085507 |
|  | Mean ± SEM | 0.039363582 ± | 0.158440883 ± | 0.141427893 ± | 0.116677752 ± | 0.119884983 ± |
|  |  | 0.038875338 | 0.156625003 | 0.140558447 | 0.114603254 | 0.119327824 |
| Concussion + | 01 | 0.000206310 | 0.000906020 | 0.042139663 | 0.000105460 | 0.001885900 |
| Ketamine | 02 | 0.006746268 | 0.021557113 | 0.071983633 | 0.022088090 | 0.004298374 |
|  | 03 | 0.006210316 | 0.007953900 | 0.054045499 | 0.015305596 | 0.008291495 |
|  | 04 | 0.000084915 | 0.000058798 | 0.000064404 | 0.000073856 | 0.000086464 |
|  | 05 | 0.000074128 | 0.000071302 | 0.000072038 | 0.000056946 | 0.000064448 |
|  | 06 | 0.000075487 | 0.000037743 | 0.000062906 | 0.000088068 | 0.000075487 |
|  | 07 | 0.000075487 | 0.000037743 | 0.000050324 | 0.000050324 | 0.000062906 |
|  | 08 | 0.000062030 | 0.000062030 | 0.000049624 | 0.000086842 | 0.000074436 |
|  | 09 | 0.000074436 | 0.000049624 | 0.000074436 | 0.000037218 | 0.000049624 |
|  | 10 | 0.000062030 | 0.000049624 | 0.000074436 | 0.000074436 | 0.000062030 |
|  | 11 | 0.000060567 | 0.000060567 | 0.000072681 | 0.000036340 | 0.000072681 |
|  | 12 | 0.000058624 | 0.000058624 | 0.000070349 | 0.000070349 | 0.000046899 |
|  | 13 | 0.000061650 | 0.000049320 | 0.000049320 | 0.000061650 | 0.000073980 |
|  | 14 | 0.000060567 | 0.000048454 | 0.000048454 | 0.000084794 | 0.000048454 |
|  | 15 | 0.000063858 | 0.000063858 | 0.000076630 | 0.000038315 | 0.000051087 |
|  | 16 | 0.000076630 | 0.000051087 | 0.000063858 | 0.000063858 | 0.000063858 |
|  | Mean ± SEM | 0.000068493 ± | 0.000053752 ± | 0.000063805 ± | 0.000063308 ± | 0.000064027 ± |
|  |  | 0.000002369 | 0.000002745 | 0.000003006 | 0.000005163 | 0.000003428 |

| **Condition** | **Case** | **Time points** |  |
| --- | --- | --- | --- |
|  |  | **50** | **60** |
| Sham + | 01 | 0.001027700 | 0.002578500 |
| Vehicle | 02 | 0.001615700 | 0.002466900 |
|  | 03 | 0.000119059 | 0.000277450 |
|  | 04 | 0.001042700 | 0.001490200 |
|  | 05 | 0.002468400 | 0.002695300 |
|  | 06 | 1.049800000 | 0.274640000 |
|  | 07 | 0.007713800 | 0.007594100 |
|  | 08 | 0.002748300 | 0.002448600 |
|  | 09 | 0.010875000 | 0.002608300 |
|  | 10 | 0.005885100 | 0.005686600 |
|  | 11 | 0.001459900 | 0.001343100 |
|  | 12 | 0.000042131 | 0.000015109 |
|  | 13 | 0.000056470 | 0.000042353 |
|  | 14 | 0.000070588 | 0.000028235 |
|  | 15 | 0.000084705 | 0.000056470 |
|  | 16 | 0.000098823 | 0.000098823 |
|  | Mean ± SEM | 0.001971041 ± | 0.001559710 ± |
|  |  | 0.000808887 | 0.000439748 |
| Sham + | 01 | 0.000044262 | 0.000044262 |
| MK-801 | 02 | 0.000088523 | 0.000029508 |
|  | 03 | 0.000341970 | 0.000332680 |
|  | 04 | 0.013401000 | 0.008362200 |
|  | 05 | 0.000656740 | 0.000702250 |
|  | 06 | 0.000116180 | 0.002328927 |
|  | 07 | 0.000524124 | 0.001939133 |
|  | 08 | 0.002296000 | 0.002568600 |
|  | 09 | 3.196100000 | 2.859800000 |
|  | 10 | 0.003374300 | 0.002872600 |
|  | 11 | 0.000311344 | 0.002223155 |
|  | 12 | 0.000059015 | 0.000044262 |
|  | 13 | 0.000154538 | 0.000112391 |
|  | 14 | 0.000098342 | 0.000084293 |
|  | 15 | 0.000140489 | 0.000112391 |
|  | 16 | 0.000028098 | 0.000112391 |
|  | Mean ± SEM | 0.201108433 ± | 0.180104315 ± |
|  |  | 0.199667837 | 0.178647168 |
| Concussion + | 01 | 0.000058423 | 0.000029212 |
| Vehicle | 02 | 0.000043817 | 0.000043817 |
|  | 03 | 0.000043817 | 0.000058423 |
|  | 04 | 0.000043817 | 0.000087635 |
|  | 05 | 0.000029043 | 0.000116171 |
|  | 06 | 0.000058086 | 0.000101650 |
|  | 07 | 0.000072607 | 0.000116171 |
|  | 08 | 0.000072607 | 0.000116171 |
|  | 09 | 0.000058141 | 0.000116282 |
|  | 10 | 0.000058141 | 0.000043606 |
|  | 11 | 0.000029071 | 0.000101747 |
|  | 12 | 0.000029071 | 0.000116282 |
|  | 13 | 0.000043164 | 0.000028776 |
|  | 14 | 0.000071940 | 0.000071940 |
|  | 15 | 0.000057552 | 0.000086328 |
|  | 16 | 0.000071940 | 0.000071940 |
|  | Mean ± SEM | 0.000052577 ± | 0.000081635 ± |
|  |  | 0.000003916 | 0.000008162 |
| Concussion + | 01 | 0.000111315 | 0.000083486 |
| MK-801 | 02 | 0.000759366 | 0.002667728 |
|  | 03 | 1.409200000 | 1.505100000 |
|  | 04 | 0.000838020 | 0.002764200 |
|  | 05 | 0.003490600 | 0.003197200 |
|  | 06 | 0.001476000 | 0.001343800 |
|  | 07 | 0.000245390 | 0.001094843 |
|  | 08 | 0.000282200 | 0.000232834 |
|  | 09 | 0.000234292 | 0.002197326 |
|  | 10 | 0.000868907 | 0.001555440 |
|  | 11 | 0.002356800 | 0.002540915 |
|  | 12 | 0.000506090 | 0.000695843 |
|  | 13 | 0.000071256 | 0.000057004 |
|  | 14 | 0.000171013 | 0.000028502 |
|  | 15 | 0.000156762 | 0.000042753 |
|  | 16 | 0.000128260 | 0.000042753 |
|  | Mean ± SEM | 0.088806017 ± | 0.095227789 ± |
|  |  | 0.088026582 | 0.093991909 |
| Concussion + | 01 | 0.001293400 | 0.001943800 |
| Ketamine | 02 | 0.014734268 | 0.001838804 |
|  | 03 | 0.005309575 | 0.001723695 |
|  | 04 | 0.000051546 | 0.000087424 |
|  | 05 | 0.000052749 | 0.000072594 |
|  | 06 | 0.000037743 | 0.000075487 |
|  | 07 | 0.000037743 | 0.000088068 |
|  | 08 | 0.000049624 | 0.000086842 |
|  | 09 | 0.000049624 | 0.000086842 |
|  | 10 | 0.000037218 | 0.000049624 |
|  | 11 | 0.000048454 | 0.000048454 |
|  | 12 | 0.000046899 | 0.000082074 |
|  | 13 | 0.000036990 | 0.000061650 |
|  | 14 | 0.000036340 | 0.000048454 |
|  | 15 | 0.000038315 | 0.000076630 |
|  | 16 | 0.000051087 | 0.000089402 |
|  | Mean ± SEM | 0.000044179 ± | 0.000073350 ± |
|  |  | 0.000001859 | 0.000004435 |
