# Supplementary material for: Intranasal delivery of the NMDA receptor antagonist MK-801 attenuates ultra-acute excitotoxic neurochemical responses after concussion in rats: comparative pharmacological evaluation against ketamine
Source: Front Pharmacol. 2026 Mar 16;17:1764201. doi: 10.3389/fphar.2026.1764201 (PMC13033605; doi:10.3389/fphar.2026.1764201)
Supplement: Supplementary file 10 [file Table10.docx]

*SUPPLEMENTARY TABLE 10:* Extracellular concentrations of serine (µg/ml).

| **Condition** | **Case** | **Time points** |  |  |  |  |
| --- | --- | --- | --- | --- | --- | --- |
|  |  | **-50** | **-40** | **-30** | **-20** | **-10** |
| Sham + | 01 | 0.588020000 | 0.571910000 | 0.429680000 | 0.414660000 | 0.341240000 |
| Vehicle | 02 | 0.753990000 | 1.053600000 | 1.058600000 | 1.045500000 | 0.961600000 |
|  | 03 | 0.330810000 | 0.186620000 | 0.190570000 | 0.046114000 | 0.058912000 |
|  | 04 | 1.578700000 | 0.667150000 | 0.673390000 | 0.659600000 | 0.618360000 |
|  | 05 | 1.388500000 | 1.426500000 | 1.654800000 | 1.162200000 | 1.305700000 |
|  | 06 | 1.017800000 | 1.121500000 | 1.167900000 | 1.196700000 | 1.512900000 |
|  | 07 | 1.095400000 | 1.010100000 | 1.055900000 | 1.066000000 | 1.089700000 |
|  | 08 | 0.497560000 | 0.491080000 | 0.508340000 | 0.616990000 | 0.524230000 |
|  | 09 | 1.550900000 | 1.264300000 | 1.024600000 | 1.114900000 | 1.250000000 |
|  | 10 | 0.614370000 | 0.631510000 | 0.635830000 | 0.640880000 | 0.704830000 |
|  | 11 | 0.139430000 | 0.060755000 | 0.052250000 | 0.058082000 | 0.065657000 |
|  | 12 | 0.367140080 | 0.283587885 | 0.274543923 | 0.287652735 | 0.310401841 |
|  | 13 | 0.619204000 | 0.309590244 | 0.446092109 | 0.471564724 | 0.348026460 |
|  | 14 | 0.350573722 | 0.309261059 | 0.336834185 | 0.338715240 | 0.373687181 |
|  | 15 | 0.383790012 | 0.445958868 | 0.356303101 | 0.473171458 | 0.282534408 |
|  | 16 | 0.375215538 | 0.373068000 | 0.315664483 | 0.386917265 | 0.313885318 |
|  | Mean ± SEM | 0.728212709 ± | 0.637905691 ± | 0.636331113 ± | 0.623727964 ± | 0.628854013 ± |
|  |  | 0.114999718 | 0.103508005 | 0.109226102 | 0.096350129 | 0.114649518 |
| Sham + | 01 | 0.286468936 | 0.300923057 | 0.319072186 | 0.323379009 | 0.281993728 |
| MK-801 | 02 | 0.189110139 | 0.197612629 | 0.120687893 | 0.186688883 | 0.184594552 |
|  | 03 | 0.804740000 | 0.750300000 | 0.831360000 | 0.795720000 | 0.813860000 |
|  | 04 | 0.454490000 | 0.281510000 | 0.347630000 | 0.264400000 | 0.247460000 |
|  | 05 | 0.612630000 | 0.599360000 | 0.555100000 | 0.704880000 | 0.826990000 |
|  | 06 | 0.870750000 | 0.930510000 | 0.941170000 | 0.832910000 | 0.823900000 |
|  | 07 | 0.405770000 | 0.797300000 | 0.574780000 | 0.501340000 | 0.548680000 |
|  | 08 | 0.832780000 | 0.820210000 | 0.837850000 | 0.930480000 | 0.744650000 |
|  | 09 | 0.769350000 | 0.752510000 | 0.735040000 | 0.950800000 | 0.984170000 |
|  | 10 | 0.301860000 | 0.513280000 | 0.299830000 | 0.365910000 | 0.287290000 |
|  | 11 | 0.497790000 | 0.775210000 | 0.646040000 | 0.812710000 | 0.445730000 |
|  | 12 | 0.195627951 | 0.229014580 | 0.039658901 | 0.236483787 | 0.200522820 |
|  | 13 | 0.118411596 | 0.149469661 | 0.166534173 | 0.161258004 | 0.135084070 |
|  | 14 | 0.170950037 | 0.192686327 | 0.157887572 | 0.171407414 | 0.142358540 |
|  | 15 | 0.142728797 | 0.150297295 | 0.135802805 | 0.168331010 | 0.157397526 |
|  | 16 | 0.187078015 | 0.118427930 | 0.119440693 | 0.193252603 | 0.138949993 |
|  | Mean ± SEM | 0.427533467 ± | 0.472413842 ± | 0.426742764 ± | 0.474996919 ± | 0.435226952 ± |
|  |  | 0.067910135 | 0.073985599 | 0.075702903 | 0.076802788 | 0.076303198 |
| Concussion + | 01 | 0.426966600 | 0.478272792 | 0.337416135 | 0.373174165 | 0.288678452 |
| Vehicle | 02 | 0.354591324 | 0.348439699 | 0.354152575 | 0.330487560 | 0.451225755 |
|  | 03 | 0.365148718 | 0.325853275 | 0.426089103 | 0.379865085 | 0.484652931 |
|  | 04 | 0.359317014 | 0.328394362 | 0.340121753 | 0.498482660 | 0.383503044 |
|  | 05 | 0.468974006 | 0.432354645 | 0.360681703 | 0.490768228 | 0.365695822 |
|  | 06 | 0.329800521 | 0.437414018 | 0.392658026 | 0.462285497 | 0.283080516 |
|  | 07 | 0.323709362 | 0.301055318 | 0.392305047 | 0.331701180 | 0.425231699 |
|  | 08 | 0.483265151 | 0.346571573 | 0.445052856 | 0.385055391 | 0.305001448 |
|  | 09 | 0.486928105 | 0.306338974 | 0.451777241 | 0.334255529 | 0.461796043 |
|  | 10 | 0.388996329 | 0.331685916 | 0.400832662 | 0.299149431 | 0.394323574 |
|  | 11 | 0.297895962 | 0.449028561 | 0.369182559 | 0.320234578 | 0.342609007 |
|  | 12 | 0.372969827 | 0.481896320 | 0.377553944 | 0.452538276 | 0.472665413 |
|  | 13 | 0.351989458 | 0.501821077 | 0.313380797 | 0.319210074 | 0.462928731 |
|  | 14 | 0.510002196 | 0.314277609 | 0.329148212 | 0.360856913 | 0.474175482 |
|  | 15 | 0.349427139 | 0.425711044 | 0.478348402 | 0.408891248 | 0.484964677 |
|  | 16 | 0.483619459 | 0.401616091 | 0.358916871 | 0.380961968 | 0.318770819 |
|  | Mean ± SEM | 0.397100073 ± | 0.388170705 ± | 0.382976118 ± | 0.382994861 ± | 0.399956463 ± |
|  |  | 0.017169717 | 0.017463307 | 0.011870982 | 0.015831422 | 0.018559283 |
| Concussion + | 01 | 0.176331940 | 0.214147013 | 0.193470112 | 0.204126610 | 0.189237878 |
| MK-801 | 02 | 0.439170000 | 0.544520000 | 0.448040000 | 0.572990000 | 0.504980000 |
|  | 03 | 0.869720000 | 0.775510000 | 0.773240000 | 0.777720000 | 0.854340000 |
|  | 04 | 0.802770000 | 0.741620000 | 0.934870000 | 0.713560000 | 0.740080000 |
|  | 05 | 0.435770000 | 0.332910000 | 0.359110000 | 0.515950000 | 0.494180000 |
|  | 06 | 0.621250000 | 0.636770000 | 0.541260000 | 0.674190000 | 0.565210000 |
|  | 07 | 0.544580000 | 0.316890000 | 0.674860000 | 0.466710000 | 0.419860000 |
|  | 08 | 0.591070000 | 0.502100000 | 0.644650000 | 0.543610000 | 0.569060000 |
|  | 09 | 0.747080000 | 0.931460000 | 0.878100000 | 0.881590000 | 0.640610000 |
|  | 10 | 1.004300000 | 0.544730000 | 0.213790000 | 0.673290000 | 0.644380000 |
|  | 11 | 0.886770000 | 1.156300000 | 0.905250000 | 0.823800000 | 0.915350000 |
|  | 12 | 0.844640000 | 0.876550000 | 0.620440000 | 0.536130000 | 0.600030000 |
|  | 13 | 0.155433189 | 0.123728104 | 0.109468532 | 0.162538171 | 0.147297461 |
|  | 14 | 0.160107374 | 0.176913495 | 0.159881382 | 0.156667879 | 0.187926492 |
|  | 15 | 0.175199259 | 0.202086847 | 0.181482951 | 0.156133214 | 0.181527047 |
|  | 16 | 0.166236730 | 0.129835411 | 0.167140700 | 0.141179129 | 0.159049068 |
|  | Mean ± SEM | 0.538776781 ± | 0.512879429 ± | 0.487815855 ± | 0.500011563 ± | 0.488319872 ± |
|  |  | 0.075739557 | 0.079788842 | 0.074025960 | 0.064863892 | 0.063103359 |
| Concussion + | 01 | 0.715020000 | 0.530830000 | 0.525510000 | 0.465740000 | 0.587920000 |
| Ketamine | 02 | 0.485490000 | 0.606350000 | 0.491610000 | 0.541190000 | 0.475410000 |
|  | 03 | 0.717370000 | 0.815790000 | 0.702600000 | 0.400660000 | 0.807500000 |
|  | 04 | 0.489445496 | 0.308824804 | 0.327326814 | 0.349638585 | 0.427618291 |
|  | 05 | 0.381528012 | 0.337869561 | 0.344816899 | 0.360607988 | 0.357674717 |
|  | 06 | 0.587797183 | 0.596801696 | 0.611846392 | 0.838995166 | 0.766671396 |
|  | 07 | 0.595262254 | 0.597562897 | 0.625537067 | 0.628861233 | 0.620546578 |
|  | 08 | 0.337710833 | 0.415188960 | 0.353955083 | 0.347055110 | 0.392414540 |
|  | 09 | 0.477045188 | 0.381482818 | 0.322801479 | 0.408009380 | 0.346613151 |
|  | 10 | 0.471011094 | 0.329052043 | 0.465049157 | 0.445440606 | 0.341246505 |
|  | 11 | 0.842876129 | 0.733578582 | 0.797010898 | 0.831588440 | 0.904353147 |
|  | 12 | 0.617944233 | 0.534283470 | 0.547648237 | 0.573274952 | 0.606242445 |
|  | 13 | 0.986490885 | 0.954414115 | 0.869276735 | 0.903689654 | 1.025713510 |
|  | 14 | 0.657653804 | 0.548345724 | 0.625804158 | 0.642515522 | 0.616738524 |
|  | 15 | 0.714750642 | 0.612178095 | 0.602370170 | 0.630230064 | 0.617695959 |
|  | 16 | 0.570001192 | 0.618031079 | 0.623387260 | 0.593232735 | 0.782913742 |
|  | Mean ± SEM | 0.602962309 ± | 0.557536490 ± | 0.552284397 ± | 0.560045590 ± | 0.604829532 ± |
|  |  | 0.042166849 | 0.044827531 | 0.040953646 | 0.045078281 | 0.052328797 |

| **Condition** | **Case** | **Time points** |  |  |  |  |
| --- | --- | --- | --- | --- | --- | --- |
|  |  | **0** | **10** | **20** | **30** | **40** |
| Sham + | 01 | 0.320640000 | 0.878810000 | 1.018500000 | 0.963070000 | 0.659890000 |
| Vehicle | 02 | 1.015900000 | 1.242400000 | 1.147300000 | 1.135900000 | 0.999340000 |
|  | 03 | 0.032707000 | 0.091785000 | 0.044440000 | 0.057496000 | 0.250240000 |
|  | 04 | 0.579680000 | 0.650900000 | 0.707780000 | 0.697000000 | 0.596130000 |
|  | 05 | 1.084000000 | 1.167200000 | 1.637400000 | 1.479100000 | 1.553100000 |
|  | 06 | 1.422400000 | 1.474000000 | 1.413300000 | 1.521300000 | 1.582200000 |
|  | 07 | 1.014500000 | 1.301500000 | 1.184200000 | 1.268400000 | 1.317100000 |
|  | 08 | 0.504090000 | 0.790770000 | 0.826610000 | 0.890810000 | 0.805450000 |
|  | 09 | 1.201500000 | 1.195800000 | 1.210300000 | 0.647520000 | 0.671680000 |
|  | 10 | 0.632490000 | 0.626970000 | 0.648910000 | 0.593960000 | 0.574080000 |
|  | 11 | 0.045321000 | 0.117030000 | 0.344940000 | 0.134670000 | 0.025291000 |
|  | 12 | 0.268834513 | 0.323489543 | 0.303965468 | 0.354767667 | 0.264536477 |
|  | 13 | 0.399912217 | 0.503715083 | 0.292590212 | 0.287777847 | 0.331700473 |
|  | 14 | 0.379322507 | 0.629683042 | 0.250909176 | 0.244090353 | 0.296681506 |
|  | 15 | 0.284493840 | 0.602384237 | 0.168119259 | 0.317670941 | 0.298460670 |
|  | 16 | 0.289368906 | 0.655821864 | 0.310938333 | 0.283804120 | 0.287730821 |
|  | Mean ± SEM | 0.592197499 ± | 0.765766173 ± | 0.719387653 ± | 0.679833558 ± | 0.657100684 ± |
|  |  | 0.106631149 | 0.104171639 | 0.124351299 | 0.120008729 | 0.120136344 |
| Sham + | 01 | 0.260367540 | 0.198026004 | 0.188010769 | 0.231698879 | 0.254144533 |
| MK-801 | 02 | 0.170698533 | 0.202193153 | 0.094663184 | 0.262567997 | 0.219152326 |
|  | 03 | 0.869320000 | 0.653490000 | 0.832640000 | 0.946510000 | 1.160900000 |
|  | 04 | 0.263870000 | 0.279330000 | 0.305780000 | 0.279460000 | 0.364290000 |
|  | 05 | 0.249790000 | 0.379890000 | 0.396970000 | 0.524210000 | 0.484510000 |
|  | 06 | 0.825870000 | 0.019199000 | 0.014877000 | 0.008616300 | 0.021422000 |
|  | 07 | 0.389640000 | 0.447230000 | 0.420410000 | 0.383310000 | 0.430050000 |
|  | 08 | 0.850240000 | 0.884790000 | 0.849950000 | 0.818810000 | 0.763470000 |
|  | 09 | 0.837910000 | 1.136500000 | 1.149400000 | 1.248500000 | 1.343900000 |
|  | 10 | 0.249550000 | 0.235670000 | 0.224070000 | 0.193630000 | 0.211740000 |
|  | 11 | 0.500950000 | 0.695890000 | 0.247980000 | 0.356738660 | 0.006290700 |
|  | 12 | 0.171973046 | 0.025574129 | 0.258079221 | 0.268354819 | 0.026958156 |
|  | 13 | 0.131190922 | 0.215037897 | 0.208667291 | 0.208313368 | 0.163828026 |
|  | 14 | 0.124891101 | 0.179585747 | 0.198986148 | 0.202645163 | 0.181731062 |
|  | 15 | 0.142592673 | 0.212467875 | 0.208607396 | 0.196987847 | 0.160043777 |
|  | 16 | 0.124602518 | 0.178224507 | 0.190143529 | 0.199002483 | 0.168058762 |
|  | Mean ± SEM | 0.385216021 ± | 0.371443644 ± | 0.361827159 ± | 0.395584720 ± | 0.372530584 ± |
|  |  | 0.073051259 | 0.078863970 | 0.077878098 | 0.082750708 | 0.098762717 |
| Concussion + | 01 | 0.404974315 | 0.571762856 | 0.289053217 | 0.320551727 | 0.340916985 |
| Vehicle | 02 | 0.391528491 | 0.538893256 | 0.301146231 | 0.421491380 | 0.388795452 |
|  | 03 | 0.400669092 | 0.372707994 | 0.376090017 | 0.221376209 | 0.373393539 |
|  | 04 | 0.385651085 | 0.552000877 | 0.432286430 | 0.230315716 | 0.360715526 |
|  | 05 | 0.330144450 | 0.507720295 | 0.372954529 | 0.253285425 | 0.323220621 |
|  | 06 | 0.343494316 | 0.411148722 | 0.476739555 | 0.396649410 | 0.254787850 |
|  | 07 | 0.353088118 | 0.354454782 | 0.327085294 | 0.382865108 | 0.318061690 |
|  | 08 | 0.330461227 | 0.440355514 | 0.396242126 | 0.425756643 | 0.358310405 |
|  | 09 | 0.330459307 | 0.542600054 | 0.422455009 | 0.417360552 | 0.385361268 |
|  | 10 | 0.351374340 | 0.475163399 | 0.328776077 | 0.431784403 | 0.332840899 |
|  | 11 | 0.405434685 | 0.365449011 | 0.439726027 | 0.429277464 | 0.304351330 |
|  | 12 | 0.336950488 | 0.457614827 | 0.360256066 | 0.287366819 | 0.263183812 |
|  | 13 | 0.311971522 | 0.410776383 | 0.420842637 | 0.402119404 | 0.320948790 |
|  | 14 | 0.329450199 | 0.428621106 | 0.450400820 | 0.425500567 | 0.377109338 |
|  | 15 | 0.400051246 | 0.507979794 | 0.258766792 | 0.313005600 | 0.345418939 |
|  | 16 | 0.376331491 | 0.399813317 | 0.381382920 | 0.439456056 | 0.413860317 |
|  | Mean ± SEM | 0.361377148 ± | 0.458566387 ± | 0.377137734 ± | 0.362385155 ± | 0.341329798 ± |
|  |  | 0.008174130 | 0.017828582 | 0.015717083 | 0.019574975 | 0.010947267 |
| Concussion + | 01 | 0.136641519 | 0.064863454 | 0.053701881 | 0.038181557 | 0.236824614 |
| MK-801 | 02 | 0.569980000 | 0.412230000 | 0.529400000 | 0.608860000 | 0.814950000 |
|  | 03 | 0.623850000 | 1.104200000 | 1.134900000 | 1.026500000 | 1.121600000 |
|  | 04 | 0.856210000 | 0.846350000 | 0.886090000 | 0.905710000 | 0.863090000 |
|  | 05 | 0.415630000 | 0.664110000 | 0.421620000 | 0.455330000 | 0.381930000 |
|  | 06 | 0.622700000 | 0.575350000 | 0.607640000 | 0.625800000 | 0.652600000 |
|  | 07 | 0.394960000 | 0.465060000 | 0.388360000 | 0.320220000 | 0.499210000 |
|  | 08 | 0.631940000 | 0.663610000 | 0.562530000 | 0.637840000 | 0.646670000 |
|  | 09 | 0.881020000 | 0.707670000 | 0.707850000 | 0.744900000 | 0.770360000 |
|  | 10 | 0.414960000 | 0.610900000 | 0.639180000 | 0.562940000 | 0.599680000 |
|  | 11 | 0.976620000 | 0.732410000 | 0.827780000 | 0.801650000 | 0.656990000 |
|  | 12 | 0.579220000 | 0.797200000 | 0.545180000 | 0.827590000 | 0.741400000 |
|  | 13 | 0.127255790 | 0.205978327 | 0.170299082 | 0.206391728 | 0.194370032 |
|  | 14 | 0.155361533 | 0.206645280 | 0.168959663 | 0.219989858 | 0.140710608 |
|  | 15 | 0.165834353 | 0.222492311 | 0.185650031 | 0.205112941 | 0.157174984 |
|  | 16 | 0.172762950 | 0.184090132 | 0.217035420 | 0.212058075 | 0.198922953 |
|  | Mean ± SEM | 0.482809134 ± | 0.528947469 ± | 0.502886005 ± | 0.524942135 ± | 0.542280200 ± |
|  |  | 0.070337072 | 0.072925133 | 0.075150094 | 0.074241819 | 0.073977452 |
| Concussion + | 01 | 0.669860000 | 0.837500000 | 0.573000000 | 0.517940000 | 0.540900000 |
| Ketamine | 02 | 0.515800000 | 0.533000000 | 0.378100000 | 0.544750000 | 0.579130000 |
|  | 03 | 0.527090000 | 4.060800000 | 0.725530000 | 0.595850000 | 0.586890000 |
|  | 04 | 0.384713164 | 0.564825043 | 0.392812157 | 0.301396012 | 0.269177966 |
|  | 05 | 0.360178350 | 0.434373903 | 0.301097024 | 0.408256519 | 0.327969638 |
|  | 06 | 0.688968029 | 0.782041207 | 0.520374828 | 0.705518224 | 0.727128538 |
|  | 07 | 0.652618922 | 0.830405899 | 0.714951197 | 0.691647721 | 0.778773298 |
|  | 08 | 0.384287905 | 0.493722378 | 0.353657437 | 0.310543880 | 0.383187517 |
|  | 09 | 0.401993326 | 0.362045639 | 0.334806530 | 0.371552268 | 0.268828358 |
|  | 10 | 0.339126905 | 0.371182466 | 0.400441959 | 0.431532425 | 0.376134211 |
|  | 11 | 0.557770358 | 0.751608634 | 0.521877749 | 0.600094829 | 0.442975040 |
|  | 12 | 0.588082304 | 0.504481301 | 0.385330572 | 0.394126684 | 0.426041086 |
|  | 13 | 1.059062919 | 1.075920948 | 0.838910689 | 0.606364354 | 0.701125196 |
|  | 14 | 0.209765261 | 1.024112432 | 0.770010234 | 0.867870019 | 0.797815939 |
|  | 15 | 0.277968587 | 0.646007896 | 0.639841212 | 0.532630698 | 0.378882298 |
|  | 16 | 0.622661015 | 0.765487878 | 0.499326731 | 0.643047720 | 0.228498884 |
|  | Mean ± SEM | 0.514996690 ± | 0.877344727 ± | 0.521879270 ± | 0.532695085 ± | 0.488341123 ± |
|  |  | 0.051606766 | 0.218998328 | 0.042848107 | 0.039142510 | 0.047177085 |

| **Condition** | **Case** | **Time points** |  |
| --- | --- | --- | --- |
|  |  | **50** | **60** |
| Sham + | 01 | 0.693640000 | 0.705590000 |
| Vehicle | 02 | 1.204400000 | 0.974960000 |
|  | 03 | 0.062485000 | 0.035143000 |
|  | 04 | 0.830740000 | 0.704840000 |
|  | 05 | 1.605600000 | 1.518900000 |
|  | 06 | 1.448600000 | 1.522100000 |
|  | 07 | 1.288900000 | 1.289100000 |
|  | 08 | 0.787680000 | 0.709390000 |
|  | 09 | 0.784950000 | 0.659240000 |
|  | 10 | 0.627740000 | 0.620100000 |
|  | 11 | 0.049869000 | 0.130840000 |
|  | 12 | 0.344305650 | 0.405100259 |
|  | 13 | 0.379894661 | 0.302794934 |
|  | 14 | 0.364195692 | 0.300616045 |
|  | 15 | 0.324090040 | 0.291312663 |
|  | 16 | 0.285105182 | 0.236660187 |
|  | Mean ± SEM | 0.692637202 ± | 0.650417943 ± |
|  |  | 0.120803212 | 0.117080348 |
| Sham + | 01 | 0.125839924 | 0.431916057 |
| MK-801 | 02 | 0.230124083 | 0.255526638 |
|  | 03 | 1.036100000 | 0.856100000 |
|  | 04 | 0.388600000 | 0.228330000 |
|  | 05 | 0.242960000 | 0.431690000 |
|  | 06 | 0.043562000 | 0.267713979 |
|  | 07 | 0.444660000 | 0.448980000 |
|  | 08 | 0.762880000 | 0.866150000 |
|  | 09 | 1.317100000 | 1.389200000 |
|  | 10 | 0.216010000 | 0.197370000 |
|  | 11 | 0.035789000 | 0.015288000 |
|  | 12 | 0.241806503 | 0.025049942 |
|  | 13 | 0.169164089 | 0.213769221 |
|  | 14 | 0.136352746 | 0.185885569 |
|  | 15 | 0.188793179 | 0.213382628 |
|  | 16 | 0.200096920 | 0.176275210 |
|  | Mean ± SEM | 0.361239903 ± | 0.387664203 ± |
|  |  | 0.091517017 | 0.090570532 |
| Concussion + | 01 | 0.405851813 | 0.434525877 |
| Vehicle | 02 | 0.360550995 | 0.457304254 |
|  | 03 | 0.362680755 | 0.476417250 |
|  | 04 | 0.264236486 | 0.275022394 |
|  | 05 | 0.364872203 | 0.388612338 |
|  | 06 | 0.410596626 | 0.433268771 |
|  | 07 | 0.341964738 | 0.248226052 |
|  | 08 | 0.394296213 | 0.444763232 |
|  | 09 | 0.410153102 | 0.282093294 |
|  | 10 | 0.415695228 | 0.434613663 |
|  | 11 | 0.339717074 | 0.279863909 |
|  | 12 | 0.410556003 | 0.432455905 |
|  | 13 | 0.388365240 | 0.454720158 |
|  | 14 | 0.276181412 | 0.369074637 |
|  | 15 | 0.294337274 | 0.279878473 |
|  | 16 | 0.315092060 | 0.283776859 |
|  | Mean ± SEM | 0.359696702 ± | 0.373413567 ± |
|  |  | 0.012600822 | 0.020753996 |
| Concussion + | 01 | 0.201124707 | 0.312052850 |
| MK-801 | 02 | 0.630630000 | 0.597420000 |
|  | 03 | 1.039800000 | 1.194700000 |
|  | 04 | 0.904420000 | 0.935100000 |
|  | 05 | 0.455110000 | 0.476860000 |
|  | 06 | 0.597050000 | 0.587610000 |
|  | 07 | 0.574660000 | 0.441730000 |
|  | 08 | 0.677960000 | 0.625000000 |
|  | 09 | 0.697550000 | 0.719430000 |
|  | 10 | 0.571730000 | 0.586820000 |
|  | 11 | 0.742500000 | 0.487510000 |
|  | 12 | 0.710680000 | 0.607870000 |
|  | 13 | 0.153873290 | 0.213309301 |
|  | 14 | 0.147253365 | 0.176676478 |
|  | 15 | 0.161639713 | 0.184062572 |
|  | 16 | 0.167647805 | 0.213722702 |
|  | Mean ± SEM | 0.527101805 ± | 0.522492119 ± |
|  |  | 0.071145357 | 0.069876436 |
| Concussion + | 01 | 0.477230000 | 0.549470000 |
| Ketamine | 02 | 0.534030000 | 0.533410000 |
|  | 03 | 0.675990000 | 0.601670000 |
|  | 04 | 0.329503107 | 0.266252543 |
|  | 05 | 0.284696247 | 0.465786801 |
|  | 06 | 0.736520473 | 0.903229034 |
|  | 07 | 0.739585726 | 0.682679443 |
|  | 08 | 0.347479029 | 0.366122486 |
|  | 09 | 0.347361775 | 0.454018220 |
|  | 10 | 0.377586362 | 0.396608641 |
|  | 11 | 0.600896561 | 0.530257070 |
|  | 12 | 0.156597731 | 0.385194722 |
|  | 13 | 0.761497421 | 0.432070336 |
|  | 14 | 0.637026436 | 0.574650749 |
|  | 15 | 0.628580171 | 0.640988639 |
|  | 16 | 0.595764034 | 0.572724832 |
|  | Mean ± SEM | 0.514396567 ± | 0.522195845 ± |
|  |  | 0.046574901 | 0.037526022 |
